# Identifying Potential *Plasmodium vivax* Sporozoite Stage Vaccine Candidates: An Analysis of Genetic Diversity and Natural Selection

**DOI:** 10.3389/fgene.2018.00010

**Published:** 2018-01-25

**Authors:** Diego Garzón-Ospina, Sindy P. Buitrago, Andrea E. Ramos, Manuel A. Patarroyo

**Affiliations:** ^1^Molecular Biology and Immunology Laboratory, Fundación Instituto de Inmunología de Colombia, Bogotá, Colombia; ^2^PhD Programme in Biomedical and Biological Sciences, School of Medicine and Health Sciences, Universidad del Rosario, Bogotá, Colombia; ^3^Basic Sciences Department, School of Medicine and Health Sciences, Universidad del Rosario, Bogotá, Colombia

**Keywords:** *Plasmodium vivax*, genetic diversity, sporozoite, vaccine, natural selection, hepatocyte invasion

## Abstract

Parasite antigen genetic diversity represents a great obstacle when designing a vaccine against malaria caused by *Plasmodium vivax*. Selecting vaccine candidate antigens has been focused on those fulfilling a role in invasion and which are conserved, thus avoiding specific-allele immune responses. Most antigens described to date belong to the blood stage, thereby blocking parasite development within red blood cells, whilst studying antigens from other stages has been quite restricted. Antigens from different parasite stages are required for developing a completely effective vaccine; thus, pre-erythrocyte stage antigens able to block the first line of infection becoming established should also be taken into account. However, few antigens from this stage have been studied to date. Several *P. falciparum* sporozoite antigens are involved in invasion. Since 77% of genes are orthologous amongst *Plasmodium* parasites, *P. vivax* sporozoite antigen orthologs to those of *P. falciparum* might be present in its genome. Although these genes might have high genetic diversity, conserved functionally-relevant regions (ideal for vaccine development) could be predicted by comparing genetic diversity patterns and evolutionary rates. This study was thus aimed at searching for putative *P. vivax* sporozoite genes so as to analyse their genetic diversity for determining their potential as vaccine candidates. Several DNA sequence polymorphism estimators were computed at each locus. The evolutionary force (drift, selection and recombination) drawing the genetic diversity pattern observed was also determined by using tests based on polymorphism frequency spectrum as well as the type of intra- and inter-species substitutions. Likewise, recombination was assessed both indirectly and directly. The results showed that sporozoite genes were more conserved than merozoite genes evaluated to date. Putative domains implied in cell traversal, gliding motility and hepatocyte interaction had a negative selection signal, being conserved amongst different species in the genus. PvP52, PvP36, PvSPATR, PvPLP1, PvMCP1, PvTLP, PvCelTOS, and PvMB2 antigens or functionally restricted regions within them would thus seem promising vaccine candidates and could be used when designing a pre-erythrocyte and/or multi-stage vaccine against *P. vivax* to avoid allele-specific immune responses that could reduce vaccine efficacy.

## Introduction

*Plasmodium vivax* (Pv) is one of the five *Plasmodium* species causing malaria in human beings [Coatney and National Institute of Allergy and Infectious Diseases (U.S.), 1971; Rich and Ayala, 2003]. Outdoor biting of less-anthropophilic mosquitos (than the main *Plasmodium falciparum* vectors) transmitting it, and endemic regions' social-economic conditions make *P. vivax* an emergent public health problem (Mueller et al., 2015). This parasite exclusively invades reticulocytes and is characterized by relapses from dormant liver stages; it produces early and continuous gametocytes (Price et al., 2009; Patarroyo et al., 2012; Adams and Mueller, 2017) and has great genetic diversity throughout its genome (Neafsey et al., 2012; Winter et al., 2015). All these features make *P. vivax* control and elimination a great challenge.

Vaccine development has been considered as one of the most cost-effective interventions for controlling malaria. Designing a vaccine against this disease has focused on selecting antigens able to induce an effective immune response that block invasion of target cells. The *Plasmodium* life-cycle should be considered when designing an anti-malarial vaccine. Malarial infection begins with an infected female mosquito's bite. *Plasmodium* sporozoites (Spz or pre-erythrocyte stage) in the vertebrate bloodstream must migrate to the host's liver, traversing the endothelial and Kupffer cells that form the sinusoidal barrier. They then migrate through some hepatocytes before infecting one of them (Menard, 2001; Frevert, 2004). Inside hepatocytes, Spz differentiate into thousands of merozoites (Mrz) which after their release proceed to invade red blood cells (RBC), initiating the erythrocyte or blood stage. Within RBC the Mrz could differentiate in new Mrz which will infect new RBC or into gametocytes which can be taken by the mosquito vector to start the sexual stage.

As mentioned above, proteins involved in parasite-host cell interactions are the main targets for vaccine development. However, the genetic diversity found in the parasite has become a challenge for designing a fully-effective vaccine (Patarroyo et al., 2012; Barry and Arnott, 2014). Such polymorphisms are typically found within functionally irrelevant gene/protein regions enabling the evasion of host immune responses, whilst functionally important regions remain conserved due to functional/structural constraints (Garzón-Ospina et al., 2012; Baquero et al., 2017); these regions could therefore be taken into account for vaccine design in order to avoid allele-specific immune responses. Several studies have measured potential vaccine candidates' genetic diversity (Putaporntip et al., 1997, 2009, 2010; Gomez et al., 2006; Garzón-Ospina et al., 2010, 2011, 2012, 2014; Dias et al., 2011; Premaratne et al., 2011; Chenet et al., 2013; Barry and Arnott, 2014; Forero-Rodriguez et al., 2014a,b; Buitrago et al., 2016; Chaurio et al., 2016; Mehrizi et al., 2017). Likewise, the evolutionary forces (mutation, natural selection, genetic drift, recombination, and migration) modulating polymorphism (Casillas and Barbadilla, 2017) have also been determined. This has been used for monitoring anti-malarial vaccine targets (Barry and Arnott, 2014) but might also be used for predicting functional regions which are usually conserved amongst species (Kimura, 1983; Graur et al., 2013). Accordingly, promising vaccine candidates must likely be parasite proteins playing an important role during target cell invasion but displaying limited genetic diversity or, at least, a domain having such pattern. These genes or domains must thus have a negative selection signal (ω < 1 evolutionary rate). Moreover, vaccine candidates should be able to induce an immune response in natural or experimental infection (Patarroyo et al., 2012; Barry and Arnott, 2014; Weiss et al., 2015).

Despite there being three intervention points [pre-erythrocyte, blood and gametocyte stages (Barry and Arnott, 2014)], potential *P. vivax* candidates characterized to date have mainly been described for the blood stage to avoid Mrz entry to RBC, preventing the disease's typical symptomatology (Patarroyo et al., 2012). On the contrary, few pre-erythrocyte phase antigens have been studied, in spite of the fact that blocking Spz interaction with hepatocytes would greatly reduce the possibility of developing the disease. This strategy would also avoid *P. vivax* dormant form formation in the liver and hence infected patients' constant relapses (Price et al., 2009; Hulden, 2011). Several Spz antigens involved in invasion have been characterized in *P. falciparum* and other parasite species (Sultan et al., 1997; Wengelnik et al., 1999; Menard, 2001; Matuschewski et al., 2002; Romero et al., 2004; Kariu et al., 2006; Labaied et al., 2007a,b; Moreira et al., 2008; Rosado et al., 2008; Engelmann et al., 2009; Alba et al., 2011; Curtidor et al., 2011; Aldrich et al., 2012; Annoura et al., 2014; Ferguson et al., 2014; Jimah et al., 2016; Kublin et al., 2017; Manzoni et al., 2017; Yang et al., 2017a). Since around 77% of *Plasmodium* genes are orthologous (Carlton et al., 2008), many being essential for parasite survival (Bushell et al., 2017), orthologous Spz antigens might also be present in the *P. vivax* genome. Given the *P. vivax* genome's high genetic diversity (Neafsey et al., 2012), *P. vivax* Spz genes might also be highly polymorphic; however, as functionally important regions tend to have low polymorphism (Kimura, 1983; Graur et al., 2013), *P. vivax* and related *Plasmodium* parasites' highly conserved regions could be functionally important and should therefore be taken into account for vaccine development (Patarroyo et al., 2012). As few *P. vivax* pre-erythrocyte stage antigens have been described to date (Menard, 2001; Castellanos et al., 2007; Barry and Arnott, 2014), and because characterizing antigens from this stage is laborious, this study involved a search for orthologous genes in *P. vivax* to those previously studied in *P. falciparum* for *in silico* characterization so as to assess their genetic diversity. The evolutionary forces modulating the variation pattern observed were analyzed for identifying conserved and functional regions; then proteins' antigenic potential was predicted for identifying those potentially able to induce an immune response and thus determine which of these antigens should be prioritized and taken into account when designing a pre-erythrocyte and/or multi-stage vaccine against malaria caused by *P. vivax*.

## Methodology

### Sequences data set and *in silico* characterization of *P. vivax* loci

Few genes encoding putative *Plasmodium vivax* Spz stage vaccine antigens have been assessed regarding their genetic diversity. Fifteen *P. falciparum* proteins have been suggested as promising vaccine candidates (Curtidor et al., 2011). Orthologous sequences to these *P. falciparum* vaccine candidates were sought in the *P. vivax* Salvador I (Sal-I) strain from the PlasmoDB database (Release 32). One hundred and seventy-one *P. vivax* natural strain DNA sequences from regions worldwide, analyzed by whole genome sequencing (Chan et al., 2012; Neafsey et al., 2012; Hester et al., 2013; Hupalo et al., 2016) available in the PlasmoDB database, were also obtained for these putative antigens. Sal-I sequences from these genes were then used as query for searching orthologous sequences in closely-related species [*Plasmodium cynomolgi* (GCA_000321355.1), *Plasmodium inui* (GCA_000524495.1), *Plasmodium fragile* (GCA_000956335.1)*, Plasmodium knowlesi* (GCA_000006355.1) and *Plasmodium coatneyi* (GCA_000725905.1)], using the whole genome data available in GenBank. Orthologous sequences from less related *Plasmodium* species (*Plasmodium berghei, Plasmodium yoelii, Plasmodium chabaudi, Plasmodium vinckei, Plasmodium falciparum, Plasmodium reichenowi, Plasmodium gaboni*, and *Plasmodium gallinaceum*) were obtained from the PlasmoDB database (Release 32).

Potential vaccine candidates described in Mrz are characterized by having a signal peptide and some have membrane anchoring structures [i.e., transmembrane helices and/or glycosylphosphatidylinositol (GPI) anchor], whilst some others have binding and/or protein-protein interaction domains (Patarroyo et al., 2012), therefore, the Sal-I Spz protein sequences were used for *in silico* characterization using several bioinformatics tools. SignalP (Nielsen, 2017) and Phobius (Kall et al., 2007) predictors were used for ascertaining signal peptide presence; the BaCelLo algorithm (Pierleoni et al., 2006) was used for predicting antigen location. Transmembrane and/or GPI domains were evaluated with Phobius, TMHMM (Sonnhammer et al., 1998) and GPI-SOM (Fankhauser and Maser, 2005) and the Pfam database was searched for putative domains.

### Alignment and sequence analysis

The 171 sequences from different locations worldwide obtained for each gene were screened to rule out gene sequences having missing data or ambiguous nucleotides; introns were removed from those genes having them. Multiple DNA alignment for each gene was performed based on amino acid (aa) information using the TranslatorX (Abascal et al., 2010) server with the Muscle algorithm (Edgar, 2004).

DnaSP v5 software (Librado and Rozas, 2009) was then used for calculating several genetic diversity estimators for 14 Spz loci. On the other hand, the effective number of codons (ENC) and codon bias index (CBI) were obtained as a measure of selective pressure at translational level (Novembre, 2002). Tajima (1989), Fu and Li (1993), and Fay and Wu (2000) tests were used for evaluating a neutral model of molecular evolution. Repeat regions or those having insertions/deletions were not taken into account for analysis. Tests based on the type of nucleotide substitution were used to infer natural selection signals within genes. The Nei-Gojobori modified method (Zhang et al., 1998) was used for calculating the difference between non-synonymous and synonymous substitution rates at intra-species level (d_N_-d_S_). The difference between non-synonymous and synonymous divergences rates (K_N_-K_S_) was calculated by modified Nei-Gojobori method with Jukes-Cantor correction (Jukes and Cantor, 1969), using *P. vivax* sequences together with orthologous sequences from phylogenetically-closely related species for determining natural selection signals at inter-species level. MEGA v6 software (Tamura et al., 2013) was used for all such analysis. A sliding window for omega rates (ω = d_N_/d_S_ and/or K_N_/K_S_) was used for evaluating the effect of natural selection throughout the gene. Likewise, individual sites (codons) under selection were identified by calculating synonymous and non-synonymous substitution rates per codon using SLAC, FEL, REL (Kosakovsky Pond and Frost, 2005), MEME (Murrell et al., 2012) and FUBAR methods (Murrell et al., 2013) in the Datamonkey online server (Delport et al., 2010). The McDonald-Kreitman (MK) test (McDonald and Kreitman, 1991) with Jukes-Cantor correction was also used for evaluating neutrality deviations by using the http://mkt.uab.es/mkt/MKT.asp web server (Egea et al., 2008).

### Lineage-specific positive selection

Lineages under episodic diversifying selection were assessed for each gene using the random effects likelihood (REL)-branch-site method (Kosakovsky Pond et al., 2011). Orthologous sequences from 13 *Plasmodium* species were aligned using the MUSCLE algorithm; this was then used for inferring the best evolutionary model using JModelTest (Posada, 2008). Phylogeny was then inferred using the Bayesian method (Ronquist et al., 2012) with a corresponding evolutionary model; these were used as reference when analyzing lineage-specific positive selection using the HyPhy package (Pond et al., 2005). CIPRES Science Gateway (Miller et al., 2010) web application was used for choosing the evolutionary model and Bayesian analysis.

### Linkage disequilibrium and recombination

Linkage disequilibrium (LD) was assessed by using the Z_nS_ estimator (Kelly, 1997), followed by linear regression between LD and nucleotide distance to ascertain whether intra-gene recombination could have taken place regarding any gene. Recombination was also evaluated using the ZZ estimator (Rozas et al., 2001), the minimum number of recombination events (Rm) (Hudson and Kaplan, 1985) and the GARD algorithm (Kosakovsky Pond et al., 2006).

### Predicting proteins' antigenic potential

A previous study has shown a correlation between predicting potential B-cell epitopes and antigenic protein regions in a *P. vivax* antigen (Rodrigues-da-Silva et al., 2017). Potential linear B-cell epitopes were therefore predicted by using the immune epitope database (IEDB) server (Kolaskar and Tongaonkar, 1990) for each protein, using Sal-I reference sequences as query.

## Results

### *P. vivax* spz loci *in silico* characterization

**Figure 1:**
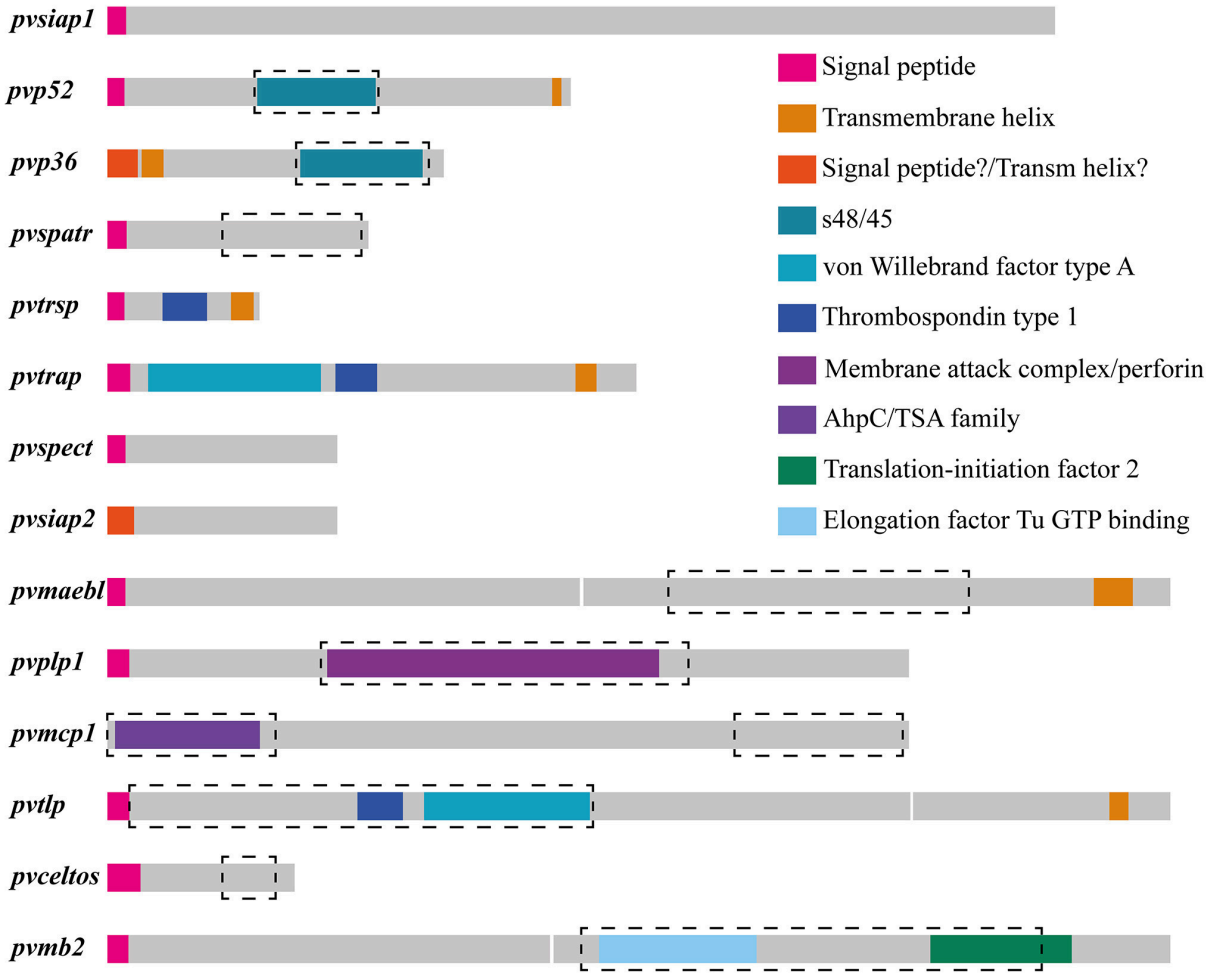
Schematic models for *Plasmodium vivax* sporozoite antigen encoding genes and their predicted functionally constrained regions. The 14 putative *P. vivax* sporozoite antigens were characterized *in silico*. Each antigen's predicted structure is shown in different colors and the predicted regions under functional constraint (highly conserved between species, having a ω < 1 and several negatively selected codons) are enclosed by a black dashed rectangle. In most cases, these regions correspond with regions having a putative domain. A region encoding a transmembrane helix was predicted for *pvp36* and *pvsiap2* at the 5′-end, but this could have been a misleading prediction (i.e., it could actually have been a signal peptide).

The currently available information for the Sal-I strain published in PlasmoDB database was used as the source for obtaining sequences from Spz genes orthologous to those identified as vaccine candidates in *P. falciparum* (Table 1). An ortholog search in other *Plasmodium* species showed that all genes (except for *siap2*, which was only present in species infecting primates) had an ortholog in the *Plasmodium* species analyzed here. The Sal-I protein sequence for each gene was then inferred from searching protein features within them (Figure 1). A positive secretion signal sequence was predicted for all antigens, except PvP36 (6-Cys protein family member), PvSIAP2 (sporozoite invasion-associated protein 2) and PvMCP1 (merozoite capping protein) proteins. In addition to signal peptide, a post-translational modification (consisting of a C-terminal GPI anchor sequence) was predicted for PvP52 (6-Cys protein family member) and PvTRSP (thrombospondin-related sporozoite protein), whilst several proteins seemed to have a transmembrane helix (Figure 1 and Table 1); transmembrane helices were found at the N-terminal end in PvP36, PvPLP1 (perforin-like protein 1) and PvSIAP2. A signal peptide and transmembrane helix were predicted at the same position in PvPLP1. The transmembrane helices in PvP36, PvPLP1 and PvSIAP2 could thus have resulted from misidentification and they could actually have been signal peptide sequences.

**Table 1:** *In silico* characterization of 14 *P. vivax* sporozoite proteins.

**Gene**	**Signal peptide (position)**	**Transm helix (position)**	**GPI**	**Domain (position)**	**Location**
*siap1*	Yes (1–22)	No	No	–	Secretory
*p52*	Yes (1–18)	No	C-ter	Sexual stage antigen s48/45 domain (159–281)	Secretory/membrane
*p36*	No	Yes (10–32 and 37–59)	No	Sexual stage antigen s48/45 domain (204–332)	Secretory/membrane
*spatr*	Yes (1–20)	No	No	–	Secretory
*trsp*	Yes (1–18)	Yes (131–154)	C-ter	Thrombospondin type 1 domain (59–105)	Secretory/membrane
*trap*	Yes (1–24)	Yes (494–515)	No	von Willebrand factor type A domain (44–225) and Thrombospondin type 1 domain (241–284)	Secretory/membrane
*spect*	Yes (1–19)	No	No	–	Secretory
*siap2*	No	Yes (7–28)	No	–	Secretory
*maebl*	Yes (1–19)	Yes (1,799–1,816)	No	–	Secretory/membrane
*plp1*	Yes (1–23)	Yes (7–26)	No	Membrane attack complex/perforin (MACPF) (232–581)	Secretory
*mcp1*	No	No	No	AhpC/TSA family (9–134)	Non cytoplasmic
*tlp*	Yes (1–23)	Yes (1,426–1,445)	No	Thrombospondin type 1 domain (264–311) and von Willebrand factor type A domain (334–508)	Secretory/membrane
*celtos*	Yes (1–35)	No	No	–	Non cytoplasmic
*mb2*	Yes (1–22)	No	No	Elongation factor Tu GTP binding domain (766–931) and Translation-initiation factor 2 (1,151–1,263)	Secretory

*The key structures for a potential vaccine candidate (such as a signal peptide, a transmembrane helix or glycosylphosphatidylinositol (GPI) anchor and functional domains) were described from the P. vivax Sal–I strain aa sequence. A protein's type of cell location is also indicated. The numbers in brackets indicate the position where a determined structure was predicted*.

PvTRSP, PvTRAP (thrombospondin-related anonymous protein) and PvTLP (thioredoxin-like protein) proteins, having a transmembrane region, also had a thrombospondin type 1 putative domain, whilst PvTRAP and PvTLP had a von Willebrand factor type A putative domain. A membrane attack complex/perforin (MACPF) putative domain was found in PvPLP1 and the AhpC/TSA domain in PvMCP1. A single sexual stage antigen s48/45 domain was predicted in PvP52 and PvP36. Both elongation factor Tu GTP (guanosine triphosphate)-binding domain and translation-initiation factor 2 were predicted for PvMB2 (Figure 1 and Table 1).

### Genetic diversity at *P. vivax*-sporozoite loci

Sequences from different regions worldwide were analyzed for quantifying Spz antigen genetic diversity (Table 2); these parasite antigens have limited genetic diversity (π < 0.003). According to the diversity parameters estimated here, *pvtrap, pvsiap2* and *pvceltos* (cell traversal protein for ookinetes and sporozoites) genes had the highest nucleotide and protein diversity values (π > 0.0015; ρ > 0.0039); *pvspect1* (sporozoite protein essential for cell traversal), *pvplp1, pvspatr* (secreted protein with altered thrombospondin repeat domain), *pvtlp, pvsiap1*, and *pvmaebl* (merozoite adhesive erythrocytic binding protein) genes formed part of the most conserved genes/proteins (π < 0.0009; ρ < 0.0020) (Table 2). The haplotype number was low in most loci. However, *pvtrap, pvsiap2, pvplp1, pvmcp1, pvtlp*, and *pvmb2* were the Spz loci with the highest haplotype number.

**Table 2:** Estimating 14 *P. vivax* sporozoite genes' genetic diversity.

***n***	**Gene**	**Sites**	**Ss**	**S**	**Ps**	**H**	**ƙ^DNA^**	**θ_W_ (SD)**	**π^DNA^ (SD)**	**ƌ^AA^**	**ρ^AA^**
15	*siap1*	2,991	12	8	4	8	2.55	0.0012 (0.0005)	0.0008 (0.0002)	2.00	0.0020
86	*p52*	1,461	9	2	7	13	1.98	0.0012 (0.0012)	0.0014 (0.0001)	1.49	0.0031
102	*p36*	1,059	6	3	3	9	1.13	0.0011 (0.0005)	0.0011 (0.0001)	0.67	0.0019
91	*spatr*	822	4	2	2	5	0.21	0.0009 (0.0005)	0.0003 (0.0001)	0.09	0.0003
63	*trsp*	480	2	0	2	3	0.59	0.0009 (0.0006)	0.0012 (0.0002)	0.59	0.0037
81	*trap*	1,668	28	9	19	20	5.11	0.0036 (0.0011)	0.0031 (0.0002)	3.91	0.0070
86	*spect1*	723	1	0	1	2	0.05	0.0003 (0.0003)	0.0001 (0.0000)	0.05	0.0002
79	*siap2*	1,239	25	8	17	25	2.09	0.0041 (0.0013)	0.0017 (0.0002)	1.99	0.0048
12	*maebl*	5,598	25	22	3	6	5.15	0.0015 (0.0006)	0.0009 (0.0005)	2.49	0.0013
73	*plp1*	2,529	13	5	8	14	0.77	0.0011 (0.0004)	0.0003 (0.0001)	0.40	0.0004
92	*mcp1*	1,455	13	4	9	19	1.44	0.0020 (0.0007)	0.0009 (0.0001)	0.84	0.0017
57	*tlp*	4,308	25	11	14	16	2.45	0.0013 (0.0004)	0.0006 (0.0001)	1.68	0.0012
101	*celtos*	588	6	3	3	10	0.89	0.0020 (0.0009)	0.0015 (0.0002)	0.76	0.0039
45	*mb2*	4,044	35	16	19	30	5.08	0.0020 (0.0006)	0.0012 (0.0001)	2.28	0.0017

*The genetic diversity estimators were calculated from a variable amount of sequences for each antigen. n: amount of sequences analyzed. Sites, total of sites analyzed, excluding gaps; Ss, amount of segregating sites; S, amount of singleton sites; Ps, amount of informative-parsimonious sites; H, amount of haplotypes; ƙ^DNA^, The average amount of nucleotide differences; θ_w_, Watterson estimator; π^DNA^, The average amount of nucleotide differences per site between two sequences; ƌ^AA^, The average amount of aa differences; ρ^AA^, The average amount of aa differences per site between two sequences; SD, standard deviation*.

### Assessing neutral evolution in *P. vivax*-sporozoite loci

The ENC and CBI parameters were evaluated to assess whether codon bias had taken place in Spz genes. These parameters gave values higher than 47 for ENC and lower than 0.47 for CBI (Table 3). Tests based on polymorphism frequency spectrum were used with the 14 Spz *P. vivax* genes to assess any departure from neutral expectations. Six of these 14 genes had an overall negative statistically significant value for at least one test (Table 3), suggesting that natural selection could have been acting regarding these genes; *pvp36* and *pvmb2* genes had specific regions within each gene having a statistical significant negative value (Supplementary Material 1).

**Table 3:** Neutrality and codon usage bias tests.

**Gene**	**Tajima**	**Fu & Li**		**Fay & Wu**	**d_N_ - d_S_ (ES)**	**K_N_ - K_S_ (ES)**	**MKT**	**ENC**	**CBI**
	**D**	**D**	**F**	**H**			**NI**		
*siap1*	−1.20	−1.71	−1.84	−0.44	0.000(0.000)	−0.153(0.010)^††^	13.6^††^	52.0	0.27
*p52*	0.27	1.29	0.94	−2.93^*^	0.000(0.000)	−0.023(0.003)^**^	2.87	53.6	0.22
*p36*	−0.36	−1.80	−1.79	0.44	−0.001(0.002)	−0.023(0.003)^††^	3.82	54.2	0.23
*spatr*	−1.42	−1.47	−1.71	−0.22	−0.001(0.001)	−0.034(0.005)^††^	1,56	49.6	0.39
*trsp*	0.64	0.72	0.81	0.46	0.002(0.001)	−0.007(0.005)	Null	50.7	0.45
*trap*	−0.48	−0.24	−0.36	−11.61^**^	0.002(0.001)	−0.014(0.004)^††^	6.46^††^	55.8	0.20
*spect1*	−0.91	0.50	0.09	0.04	0.000(0.000)	−0.014(0.003)^††^	Null	47.5	0.29
*siap2*	−1.80^*^	−0.75	−1.35	−2.07	0.002(0.001)^***^	−0.003(0.003)	Null^†^	47.5	0.36
*maebl*	−1.68	−1.20	−1.64	−10.21^**^	−0.001(0.000)^**^	−0.066(0.007)^††^	0.39^†^	49.1	0.30
*plp1*	−2.11^*^	−0.78	−1.46	−3.16^*^	−0.002(0.001)^*^	−0.033(0.003)^*^	2.09	52.3	0.28
*mcp1*	−1.19	−1.44	−1.64	0.57	−0.001(0.001)	−0.045(0.005)^††^	1.64	47.8	0.28
*tlp*	−1.76	−1.39	−1.95	−5.22^**^	−0.0001(0.000)	−0.051(0.004)^††^	1.81	52.7	0.20
*celtos*	−0.80	−0.82	−0.87	−1.49	0.001(0.001)	−0.036(0.0027)	6.36	53.8	0.35
*mb2*	−1.31	−1.67	−1.84	−3.41	−0.001(0.001)^*^	−0.074(0.004)^††^	1.26	53.4	0.25

Selective pressure on each gene was inferred from neutrality tests and estimating effective codon usage. Non-synonymous substitution rate (d_N_) and synonymous substitution rate (d_S_) in P. vivax. Non-synonymous (K_N_) and synonymous (K_S_) divergence between P. vivax and the phylogenetically closest specie. Neutrality index (NI) estimated by McDonald-Kreitman test, using Jukes-Cantor correction. The preferential use of the synonymous codons was evaluated by estimating the effective amount of codons (ENC) and the codon bias index (CBI). Genes under selection in the tests had

* *p < 0.05*,

** *p < 0.01*,

† *p < 0.03*,

*** p < 0.006, and

†† *p < 0.0001*.

Regarding d_N_-d_S_ rates, negative values were found for *pvmaebl, pvplp1*, and *pvmb2* whilst positive values was observed for the *pvsiap2* gene (Table 3). When species divergence was assessed, there was evidence of inter-specific negative selection for all genes, except *trsp, siap2*, and *celtos*; the sliding window for ω rate (Supplementary Material 2) gave values lower than 1 for these genes. Likewise, codon-based methods identified several codons under negative selection with few codons under positive selection. Many codons under negative selection were located in regions encoding putative domains (e.g., thrombospondin type 1 domain, Supplementary Material 2). It was found that *siap1* and *trap* loci had statistically significant values higher than 1 when polymorphism and divergence were compared by MK test, whilst a value lower than 1 was observed for *maebl*.

### Lineage-specific positive selection

*Plasmodium* species orthologous sequences were used for constructing phylogenies to assess whether positive natural selection had taken place during antigen evolutionary history (Supplementary Material 3). Topologies gave three monophyletic clusters (*siap2* did not, since it is only present in primate-infecting parasites); the first involved monkey-malaria parasites, the second clustered *Plasmodium* species infected rodents and the third was formed by hominid-malaria parasites. The latter cluster and the rodent clade represent *Plasmodium* phylogenetic relationships. However, the monkey-malaria parasite topology had different branch patterns regarding malaria-species relationships which could have resulted from positive selection (Sawai et al., 2010). Nine of these topologies had lineages (branches) where some sites were under positive selection. Most branches having evidence of positive selection lead to a particular species, whilst few of them were ancestral lineages (Supplementary Material 3).

### Linkage disequilibrium and recombination

Linkage disequilibrium (LD) at intra-gene level was assessed by Zns estimator (Table 4). Non-random associations between SNPs were found for *pvmaebl* but not for the remaining genes. However, there was a decreasing linear regression tendency between LD and nucleotide distance in all genes, suggesting recombination action. Likewise, most of these genes had at least one recombination event (RM), while the ZZ estimator just gave statistically significant values for *pvp52* and *pvspatr*. The GARD algorithm gave one recombination breakpoint for *pv52, pvtrap, pvplp1, pvtlp*, and *pvceltos* and two recombination breakpoints for *pvmb2* (Table 4).

**Table 4:** LD and intra-gene recombination estimators.

**Gene**	**Zns**	**ZZ**	**RM**	**GARD**
*siap1*	0.12	−0.00	1	–
*p52*	0.08	−0.06^*^	2	369^†^
*p36*	0.01	−0.00	1	–
*spatr*	0.54	0.18^*^	0	–
*trsp*	0.04	0.00	0	–
*trap*	0.10	0.06	22	1,200^†^
*spect1*	–	–	–	–
*siap2*	0.03	−0.02	2	–
*maebl*	0.73^*^	0.16	0	–
*plp1*	0.03	−0.01	2	1,275^**^
*mcp1*	0.02	−0.02	2	–
*tlp*	0.08	0.12	2	2,551^†^
*celtos*	0.02	0.01	0	355^†^
*mb2*	0.04	0.04	7	1,368^†^ and 2,605^†^

*GARD, recombination breakpoint position*.

* *p < 0.04*,

** *p < 0.005*,

† *p < 0.001*.

### *P. vivax*-spz proteins' antigenic potential

Previous studies have shown a B-epitope and solvent accessibility prediction correlation with antigenic regions in natural infections (Rodrigues-da-Silva et al., 2017). The Sal-I sequences for each Spz protein studied here were thus analyzed by the BepiPred server for determining their antigenic potential (Supplementary Material 4). Large-sized proteins, such as MAEBL, TLP, TRAP, P52, and MCP1, had regions toward the C-terminal which could be recognized as linear B-epitopes; these aa regions were the regions having greater solvent accessibility (Supplementary Material 4). It was seen that the most exposed PLP1 region was located toward the N-terminal. Smaller proteins, such as SPECT1, SPART, and SIAP1/2, did not seem to have clearly-defined recognition regions all along their sequences, whilst 3 regions having antigen potential were observed for CelTOS (Supplementary Material 4). Many P36, TRSP, PLP1, MB2 antigen sequences seemed to be exposed and several regions having potential B-epitopes were found.

## Discussion

A prospective *Plasmodium vivax*-malarial vaccine has been delayed regarding *P. falciparum*; however, knowledge acquired concerning the latter species could be useful for designing a fully-effective anti-*P. vivax* vaccine. Vaccine development involves several challenges; for instance, high antigen diversity has made vaccines not fully-protective since polymorphism provokes allele-specific immune responses; genetic diversity is therefore an immune avoidance mechanism. Consequently, conserved antigens (or regions within them) should be used as vaccines candidates to avoid this kind of response (Richie and Saul, 2002; Patarroyo et al., 2012). Even more, just one antigen might not be enough to produce full protection regarding a particular vaccine, so several antigens would be necessary. Since malaria parasites have multiple stages, a fully-effective vaccine must have several conserved antigens (or regions containing them) from different parasite stages.

Antigen identification is not an easy task due to *P. vivax* having a complex biology. Most antigens described to date regarding this parasite have been from the blood stage; few genes/proteins from others stages have been characterized. Although *P. vivax* biology cannot be assessed directly, some new technologies enable making inferences about it. Whole genome sequences could be used to infer the genes in a particular parasite and thus gene ontology could provide clues about its biology. At least 15 *P. falciparum* Spz stage proteins are involved in parasite invasion and could thus become vaccine candidates. Since many genes are shared amongst *Plasmodium* species (Carlton et al., 2008), and several of them seem to be essential for parasite survival (Bushell et al., 2017), orthologs to the 15 *P. falciparum* Spz antigens might be present in the *P. vivax* genome and could thus be taken into account for vaccine development. Fourteen of the 15 *P. falciparum* Spz genes found in *P. vivax* had similar gene/protein structures, suggesting that they could be involved in a conserved *Plasmodium* Spz invasion pathway. However, gene/protein identification is the first step in vaccine design. As *P. vivax* genetic diversity represents an even greater challenge than in *P. falciparum* concerning vaccine design, the next step in this work was to assess the genetic diversity of these Spz loci using available whole genome sequences to find out which of them could be promising vaccine candidates against *P. vivax*.

### *P. vivax* spz loci genetic diversity

It has been suggested that the *P. vivax* genome's large genetic diversity would hinder *P. vivax* control and elimination. Several *P. vivax* blood-stage antigens have been considered as vaccine candidates; however, they have high genetic diversity (Putaporntip et al., 1997, 2010; Gomez et al., 2006; Dias et al., 2011; Premaratne et al., 2011; Garzón-Ospina et al., 2012, 2014, 2015), representing one of the challenges to be overcome when designing a completely-effective anti-malarial vaccine. Contrasting with the aforementioned antigens, the Spz loci analyzed here had low genetic diversity (π < 0.003) which is a desirable feature when designing a fully-effective antimalarial vaccine. Few segregating sites were found at each locus; some were singleton sites. This could have resulted from genetic differentiation amongst *P. vivax* populations worldwide, as has been shown previously (Taylor et al., 2013; Hupalo et al., 2016). Nevertheless, fully-conserved regions were also identifiable in all loci. The observed polymorphism in Spz genes was comparable to that found in the most of the conserved Mrz genes described to date (Putaporntip et al., 2009; Garzón-Ospina et al., 2010, 2011, 2015; Pacheco et al., 2012; Chenet et al., 2013; Forero-Rodriguez et al., 2014a,b; Buitrago et al., 2016) being *pvspect, pvspart, pvsiap1, pvplp1*, and *pvtlp* the loci with the lowest diversity. The most polymorphic gene was *pvtrap*; its diversity pattern has previously been reported for the *P. falciparum* ortholog (Ohashi et al., 2014).

### Evolutionary forces modulating *P. vivax* sporozoite diversity

Contrasting with Mrz proteins (Putaporntip et al., 1997, 2010; Gomez et al., 2006; Dias et al., 2011; Garzón-Ospina et al., 2012, 2014, 2015), Spz antigens evaluated here displayed low diversity. This could have resulted from various evolutionary forces. Taking into account that a low amount of synonymous and non-synonymous polymorphism was found, low diversity could have been a consequence of codon usage bias. ENC and CBI parameters were thus estimated for evaluating such hypothesis. ENC values close to 61 indicated that all synonymous codons for each aa were used equitably (values close to 0 suggest bias or preferential codon use). CBI values range from 0, meaning uniform synonymous codon usage, to 1 (i.e., maximum codon bias) (Morton, 1993). Spz genes' ENC and CBI values suggested that all of them had random synonymous codon usage. Such values were similar to those previously described for genes participating in Mrz invasion and the value reported for the complete genome (Cornejo et al., 2014). This meant continuous transcription related to these proteins' level of expression during invasion (Gajbhiye et al., 2017; Uddin, 2017) which was not affected by any type of selective pressure or preferential codon usage. The low genetic diversity found in Spz genes was therefore not a consequence of selection at translation level and thus other evolutionary forces must be causing low genetic diversity in theses antigens.

Test of neutral molecular evolution (e.i. Tajima, Fu & Li, Fay & Wu) gave negative values, just a few being statistically significant; the neutrality could not thus be ruled out in antigens lacking significant values. Since these genes were highly conserved, a functional/structural constraint was likely (Kimura, 1983; Graur et al., 2013). Although some genes had no statistically significant values, some of them had specific regions where neutrality could be ruled out, suggesting that negative selection was acting in such regions. Likewise, genes having an overall statistically significant negative value in these tests suggested that negative selection was responsible for low protein diversity and consequently functional/structural constrains would also be expected under this kind of selection. This was confirmed when non-synonymous and synonymous rates, as well as evolutionary rate (ω) sliding windows, were computed. Synonymous mutations seemed to fix at a higher rate than non-synonymous ones after speciation involving monkey-malaria parasites; this thus agreed with a hypothesis of functional/structural constraint. Furthermore, several negative selected sites (codons) were found; many were located in putative functional domains, suggesting that negative selection is an important force for maintaining protein domain integrity. These regions had statistically significant negative values in the tests based on polymorphism frequency spectrum (e.i. Tajima, Fu & Li, Fay & Wu). These patterns could thus be used to predict functionally important regions. A recent report regarding *P. vivax* showed that regions having low ω rates having several negatively selected sites are regions used by the parasite to recognize host cells (Baquero et al., 2017). Consequently, the regions from the 14 antigens considered here having low ω rates and several codons under negative selection could be those used to recognize hepatocytes. However, these regions might not necessarily be involved in host-parasite interaction and they could have other functions. However, they could be taken into account for *P. vivax* vaccine development since they had low diversity and were under functional/structural constraint. The aforementioned results could then also be used for elucidating aspects of *P. vivax* sporozoite invasion of target cells by assessing the domains having functional constraint in invasion assays.

Few positively selected sites were found; they could have been fixed to adapt to different selective pressures. Molecular adaptations could play an important role during parasite evolutionary history. Monkey-malaria clades diversify three to four times more rapidly than those infecting other mammalians (Muehlenbein et al., 2015). Taking host species' rapid diversification into account (Ziegler et al., 2007), adaptive radiation in monkey-malaria parasites could explain this accelerated cladogenesis and therefore several molecular adaptations could have arisen during such radiation. Phylogenies inferred for Spz antigens showed that monkey-malaria parasite topology did not agree with malaria-species relationships which could have resulted from positive selection (Sawai et al., 2010). There was evidence of selective sweep in five *P. vivax* genes; this pattern has already been observed for some of these genes in previous studies (Shen et al., 2017). Some mutations fixed in this parasite would thus allow it to adapt to a new host after host-switch decreasing genetic diversity.

Additionally, phylogenetically-based analysis could provide greater insight into the role of selection (at these loci) during a parasite's evolutionary history if this was the result of adaptations to new hosts and/or environments (Muehlenbein et al., 2015). There was evidence of episodic positive selection for nine of these 14 antigens. Throughout phylogenies, several lineages had some sites under positive selection; however, few of them were ancestral lineages. Since most branches under selection lead to particular species, episodic selection could have resulted from adaptation to different host, for instance, to avoid immune response to a particular host during sympatric speciation or to recognize a new host receptor. However, this behavior seems to be common in the *Plasmodium* genus and not just for monkey-malaria parasites.

Recombination is an evolutionary force which can increase genetic diversity. It could be acting on Mrz blood-stage antigens leading to new haplotypes to arise, being maintained in the parasite population to evade the host's immune responses (Garzón-Ospina et al., 2012). Even though low genetic diversity was observed, some Spz genes had a large amount of haplotypes. Nevertheless, larger-sized genes accumulated a greater amount of single nucleotide polymorphisms in their sequences; these characteristics were not related to the amount of haplotypes. Some haplotypes could thus have arisen by recombination. Evidence of intra-gene recombination was found for *pvp52, pvplp1, pvtlp, pvceltos*, and *pvmb2* genes. Recombination is thus an evolutionary force in these loci increasing genetic diversity.

### *P. vivax* spz antigens' putative roles

The mechanism by which these Spz proteins act in *P. vivax* is not certain; however, their possible role could be elucidated from them having putative domains. SPECT, PLP1, and TLP seem to be essential in different *Plasmodium* species for traversing host cells (Ishino et al., 2004; Yang et al., 2017a) since deleting them has reduced parasite capability to traverse the sinusoidal barrier and thus gain access to hepatocytes (Ishino et al., 2004; Kaiser et al., 2004). No described domains were identified for PvSPECT and PvSPART and their role thus requires further investigation. Nevertheless, orthologs to these proteins have been implicated in parasite interaction with epithelial or hepatic host cells in *P. falciparum* (Curtidor et al., 2011). Even though putative protein-protein interaction domains were not identified, the regions involved in such interaction can be predicted regarding their degree of conservation between species, as can their evolutionary rates (ω) (Graur et al., 2013; Baquero et al., 2017). According to the sliding window for the gene encoding to SPART in *P. vivax*, the C-terminal region seemed to be functionally restricted as low genetic diversity (intra- e inter-species) was observed, as well as lower than 1 ω rate and various sites under negative selection (at inter-specific level); this region could be implicated in interaction with hepatocytes. Additionally, PfSPART is antigenic in natural infections and antibodies against it have blocked interaction with hepatocytes *in vitro* (Palaeya et al., 2013). Similar to PfSPATR, PvSPATR has antigenic potential given the 4 regions along its sequence having high solvent accessibility values and potential linear B-epitopes, suggesting that PvSPART is a promising antigen when designing an anti-*P. vivax* vaccine.

Similar to the aforementioned antigens, particular domains were not found in the whole PvSIAP1 sequence. Orthologs for this protein in *Plasmodium* spp, are involved in Spz exit from oocysts, as well as their colonization in a mosquito's salivary glands (Engelmann et al., 2009). SIAP1 in a vertebrate host seems to be mediated by pathogen-host interaction (Curtidor et al., 2011). This protein's ortholog in *P. vivax* has been shown to have low diversity (ω < 1) throughout its sequence, as well as several negatively-selected sites. However, putative functional regions have not been clearly defined due to a large amount of sites under positive selection all along the sequence of the gene encoding this protein. Nevertheless, the C-terminal region was the region where most codons were found to be under negative selection and could thus be the region used for *P. vivax* interaction with a particular host.

PLP1 is not required for entry to hepatocytes, but does play an important role in exit from transitory vacuoles during cell traversal (Risco-Castillo et al., 2015). A putative MAC/perforin domain was identified in *P. vivax* which had a high sequence conservation, several codons under negative selection and a ω < 1; this functionally restricted region could thus be mediating membrane destabilization and pore formation in *P. vivax*, as has been suggested in other species (Rosado et al., 2008; Patarroyo et al., 2016; Yang et al., 2017a).

It is well known that the TLP (Moreira et al., 2008) and TRAP (Sultan et al., 1997) are essential for Spz gliding motility (Sultan et al., 1997). *P. falciparum* TLP is the most conserved member of the TRAP/MIC2 family and the first to be seen to play a role traversing cells (Moreira et al., 2008). *P. vivax* TLP was one of the most conserved proteins of those evaluated here; the N-terminal region was highly conserved within and amongst species. Thrombospondin type 1 (TSP1) and von Willebrand factor type A (vWa) domains were observed in this region. The *P. falciparum*, TSP1 domain mediates glycosaminoglycan binding whilst the vWa domain is involved in cell-cell, cell-matrix, matrix-matrix interactions and includes a metal-ion dependent adhesion site. Proteins containing such domains might be associated with parasite invasion ability (Wengelnik et al., 1999; Matuschewski et al., 2002; Mongui et al., 2010); the PvTLP N-terminal region could thus be mediating *P. vivax* Spz invasion of hepatocytes.

Unlike a PvTLP, PvTRAP (having the same domains toward the N-terminal region) was the protein having the greatest diversity of those evaluated here. This gene is highly polymorphic in *P. falciparum* and is under positive selection (Ohashi et al., 2014); the region encoding the vWA domain is where *pftrap* diversity is concentrated (Moreira et al., 2008). Such pattern seems to be similar in *P. vivax* and its related species; the sliding window for this gene showed that this region evolved rapidly, having several sites under positive selection amongst species. This could have resulted from lineage-specific adaptations for the recognition of particular receptors for each host or is the target region for immune responses, similar to that which occurs in PvDBP (VanBuskirk et al., 2004; Chootong et al., 2014). A prediction of B-epitopes and solvent accessibility showed that TRAP should have a potentially antigenic region toward the N-terminal where the vWa domain would be located. However, the C-terminal region seemed to have greater antigenic potential. According to previous studies PvTRAP can induce an IgG1 and IgG3 response following natural infection; producing this type of antibody is usually correlated with protection against disease in a hyper-endemic region (Nazeri et al., 2017). This protein has thus been proposed as being an important immune response target regarding pre-erythrocyte stages of malaria caused by both *P. falciparum* and *P. vivax* (Ohashi et al., 2014). Nevertheless, the diversity observed in this protein could produce allele-specific immune responses and would thus not be a good vaccine candidate.

Unlike TRAP and TLP, PvTRSP only has the TSP1 domain, this protein is involved in *P. berghei* invasion where the deletion of *pbtrsp* reduces mutant parasite capability to enter hepatocytes (Labaied et al., 2007a). This gene was highly conserved in *P. vivax*; however, when the sequences from *P. vivax* and related species were compared, the TSP1 domain was seen to have a ω > 1 and few sites under negative selection. On the other hand, the C-terminal region seemed to be this protein's antigenic region.

MAEBL has been described as being a type I membrane protein in *P. falciparum*, having erythrocyte-binding activity and seeming to fulfill a function similar to that of AMA1, given the high sequence similarity (Yang et al., 2017b). This protein is necessary for Spz invasion of a mosquito's salivary glands and for them to traverse vertebrate host cells (Yang et al., 2017b). MAEBL has 4 tandem repeats in *P. vivax*, located toward the gene's 3′ region, between positions 3,427 and 4,756. Each repeat has 90% similarity; 9 imperfect copies of GCTAGAAGGGCTGAGGAGT residues, 3 copies of AGAAAGGCGGAAGAGGCA, 17 copies of GCAAGGAAGGCAGAGGATGCTAGAAAGGCAGAGGCGGCTA and 6 copies of GCTAAAAAGGCTGAAGCAGCAAGGAAGGCAGAGGCA residues were found. In spite of an accumulation of repeats being responsible for size polymorphism, they did not show such polymorphism when sequences from different isolates were analyzed. Calculating the omega rate between *P. vivax* isolates and related species revealed that such repeats were highly conserved in *Plasmodium*, as has been suggested previously (Leite et al., 2015). The fact that this repeat region was found to be under negative selection suggested an important role, similar to that already reported for CSP (Aldrich et al., 2012; Ferguson et al., 2014). A prediction of linear epitopes suggested that the C-terminal region where these repeats were located could be antigenic. Even though the repeats have been suggested as targets distracting an immune response, this is still not clear because they are highly conserved, even between species. Conversely, the *maebl* 5′-end was highly divergent amongst species, having several sites under positive selection, possibly resulting from species-specific adaptations.

MB2 (just like MAEBL) is one of the largest proteins expressed in *Plasmodium* spp Spz and is involved in hepatocyte invasion (Nguyen et al., 2009). It contains a characteristic GTP-binding domain (Nguyen et al., 2001; Romero et al., 2004), that is also present in its *P. vivax* counterpart; in addition, a translation-initiation factor domain was predicted for PvMB2. Both were functionally restricted, being conserved in *P. vivax* as well as between phylogenetically-related species. Even though it is not clear whether these domains are mediated by pathogen-host interaction, they do seem to be important for this protein's function. This protein is antigenic in *P. falciparum* and has been recognized by patients showing protection against *Plasmodium* infection following experimental immunization (Nguyen et al., 2009). The region so recognized is the N-terminal region, which had antigenic potential in *P. vivax*, given its solvent accessibility and the prediction of B-linear epitopes. Given low PvMB2 diversity and its antigenic potential, it could also be taken into account when designing a vaccine against *P. vivax*.

Members of the 6-cys family are expressed during different *Plasmodium* spp. stages. Two members of this family (P36 and P52) are expressed during the pre-erythrocyte stage and seem to play an important role in invasion (Labaied et al., 2007b; Annoura et al., 2014; Kublin et al., 2017; Manzoni et al., 2017). Like 6-Cys members expressed in *P. vivax* Mrz (Forero-Rodriguez et al., 2014a,b), *pvp36* and *pvp52* displayed low genetic diversity, accompanied by low evolutionary rates. The s48/45 domains, characteristic of this family, seemed to be functionally restricted (limited diversity, ω < 1 and various sites under negative selection) and thus might have been responsible for the interaction between *P. vivax* Spz and hepatocytes. The proteins encoded by these genes also have antigenic potential; the PvP52 C-terminal region seemed to be exposed, having potential linear B-epitopes, whilst the PvP38 central region and C-terminal could be antigenic regions. Thus, the same as other 6-Cys family members (Forero-Rodriguez et al., 2014a,b), PvP36 and PvP52 would seem to be promising antigens when designing a vaccine.

In addition to TRAP, SIAP2, and CELTOS were the antigens having the highest diversity values amongst those evaluated here. These proteins are predominantly expressed during the Spz stage in other species where they cover parasite surface (Siau et al., 2008). Unlike the other proteins evaluated here, SIAP2 only had orthologs in *Plasmodium* species infecting primates. This is a potential vaccine candidate as it seems to interact specifically with heparin sulfate and chondroitin sulfate-type membrane receptors (Siau et al., 2008; Alba et al., 2011). Potential functional regions could not be defined for SIAP2 (like TRAP) since it had many sites under positive selection amongst species throughout its sequence. Predicted results regarding PvSIAP2 antigenicity suggested that this could be exposed to the immune system, having various potential B-epitopes along its sequence. Its use as vaccine candidate is limited due to its diversity and the large amount of haplotypes found.

CelTOS has been considered a potential vaccine candidate given its association with clinical protection regarding naturally-acquired immunity (Kanoi et al., 2017). This protein has a sole specificity for phosphatidic acid (a lipid found predominantly on plasma membrane inner face) and breaks liposomes consisting of phosphatidic acid through pore formation. It has been shown that parasites lacking CelTOS can enter target cells but remain trapped inside. The foregoing supposes that CelTOS targets cell membrane inner leaflet which can burst due to pore formation inside infected cells and favors parasite exit (Kariu et al., 2006; Jimah et al., 2016). Even though its function seems to be intracellular, predictive analysis suggested that PvCelTOS could be exposed to the immune system, having potential B-epitopes toward the C-terminal region.

A recent study has shown a correlation between the prediction and this protein's antigenic regions. Naturally-infected patients generate immune responses against PvCelTOS, predominantly toward the protein's C-terminal region (Rodrigues-da-Silva et al., 2017), coinciding with the most exposed regions and having potential B-epitopes. This would suggest that PvCelTOS is a potential antigen for inclusion in a vaccine against *P. vivax*. The antigenic region coincided with the gene's region where various sites under negative selection were found, suggesting that the CelTOS functional region could be located in the C-terminal region; however, some sites under positive inter-species selection were found toward this region. If this region is functionally restricted, but is also the region toward which the immune response is directed in different species, then some sites could have been positively selected in a species-specific manner fixing specific mutations in each species, resulting in a positive selection signal. In addition to its antigenic potential, PvCelTOS has been shown to have limited diversity within *P. vivax*. The gene encoding this protein in Iran had, on average, less than one mutation in paired comparisons (Mehrizi et al., 2017); such results were similar to those reported here. PvCelTOS might thus be considered a vaccine candidate, given its antigenic characteristics and limited diversity.

## Conclusions

Designing a completely effective vaccine against *P. vivax* must include antigens or highly conserved regions containing them to abolish allele-specific immune responses; antigens from different stages must also be included. As most research has been focused on the blood stage, new strategies must be implemented for identifying potential vaccine candidates from other parasite stages. As genetic diversity is a characteristic to be born in mind when designing a vaccine, the present work could be taken as a basis for selecting Spz antigens which might become potential vaccine candidates.

*P. vivax* genetic diversity would be expected to be high as it has been shown in whole genome analysis (Neafsey et al., 2012) and in several Mrz antigens (Gomez et al., 2006; Putaporntip et al., 2010; Dias et al., 2011; Premaratne et al., 2011; Garzón-Ospina et al., 2012). However, this study's results showed that some Spz antigens had limited genetic diversity and, consequently, they could be good vaccine candidates, since low genetic diversity is ideal for avoiding an allele-specific immune response. The analysis described above enabled determining which regions in these proteins could be functionally important. Recent studies have shown that regions predicted to have functional restriction coincide with regions used by the parasite to bind to a host cell (Baquero et al., 2017). Therefore, regions in Spz antigens which were conserved between species having low ω values and codons under negative selection in the parts of a protein might be thus implicated in these antigens' function. This could help to elucidate aspects of *P. vivax* Spz invasion. Further assays regarding interaction with hepatocytes are thus required for determining whether such regions are involved in pathogen-host interaction.

Promising antigens to be taken into account for designing a fully effective vaccine are those having a limited genetic diversity or at least one domain with such pattern. These genes or domains must have a negative selection signal, as well as a ω < 1 (Garzón-Ospina et al., 2015). Thus, the results reported here suggest that the PvP52, PvP36, PvSPATR, PvPLP1, PvMCP1, PvTLP, PvCelTOS, and PvMB2 antigens (or functionally restricted regions of them) are promising vaccine candidates and thus should be prioritized in future studies aimed at developing a completely effective vaccine against *P. vivax*.

## Author contributions

DG-O, SB, and AR acquired the genomic data and carried out the genetic diversity analyses. DG-O performed the general interpretation of the data and together with SB and AR participated in writing the paper. MP coordinated the study and helped to write the manuscript. All the authors have read and approved the final version of the manuscript.

### Conflict of interest statement

The authors declare that the research was conducted in the absence of any commercial or financial relationships that could be construed as a potential conflict of interest.

## Abbreviations

BepiPred: B-cell epitope prediction
CBI: codon bias index
ENC: effective number of codons
FEL: fixed effects likelihood
FUBAR: fast, unconstrained Bayesian approximation for inferring selection
GARD: genetic algorithm recombination detection
GPI: glycosylphosphatidylinositol membrane anchor
GTP: guanosine triphosphate
IEDB: immune epitope database and analysis resource
IFEL: internal fixed effects likelihood
MEME: mixed effects model of evolution
Mrz: merozoite
*pvspect1*: gene encoding *Plasmodium vivax* sporozoite protein essential for cell traversal
*pvsiap1*: gene encoding *Plasmodium vivax* sporozoite invasion-associated protein-1
*pvp52*: gene encoding *Plasmodium vivax* 6-cysteine protein P52
*pvp36*: gene encoding *Plasmodium vivax* 6-cysteine protein P36
*pvspatr*: gene encoding *Plasmodium vivax* secreted protein with altered thrombospondin repeat domain
*pvtrsp*: gene encoding *Plasmodium vivax* thrombospondin-related sporozoite protein
*pvtrap*: gene encoding *Plasmodium vivax* thrombospondin-related anonymous protein
*pvsiap2*: gene encoding *Plasmodium vivax* sporozoite invasion-associated protein-2
*pvmaebl*: gene encoding *Plasmodium vivax* merozoite adhesive erythrocytic binding protein
*pvplp1*: gene encoding *Plasmodium vivax* perforin-like protein 1
*pvmcp1*: gene encoding *Plasmodium vivax* merozoite capping protein 1
*pvtlp*: gene encoding *Plasmodium vivax* thioredoxin-like protein
*pvceltos*: gene encoding *Plasmodium vivax* cell traversal protein for ookinetes and sporozoites
*pvmb2*: gene encoding *Plasmodium vivax* MB2 protein
REL: random effects likelihood
SLAC: single likelihood ancestor counting
SNP: single nucleotide polymorphisms
Spz: sporozoite.
