# Supplementary material for: Identifying Potential Plasmodium vivax Sporozoite Stage Vaccine Candidates: An Analysis of Genetic Diversity and Natural Selection
Source: Front Genet. 2018 Jan 25;9:10. doi: 10.3389/fgene.2018.00010 (PMC5788960; doi:10.3389/fgene.2018.00010)
Supplement: Supplementary file 1 [file Presentation1.PDF]

Supplementary Material 1. Sliding window analysis of neutral tests based on polymorphism frequency spectrum

Identifying potential *P. vivax* sporozoite stage vaccine candidates: an analysis of genetic diversity and natural selection

Diego Garzón-Ospina, Sindy Paola Buitrago, Andrea Estefania Ramos, Manuel A. Patarroyo*

*** Correspondence:** mapatarr.fidic@gmail.com

A

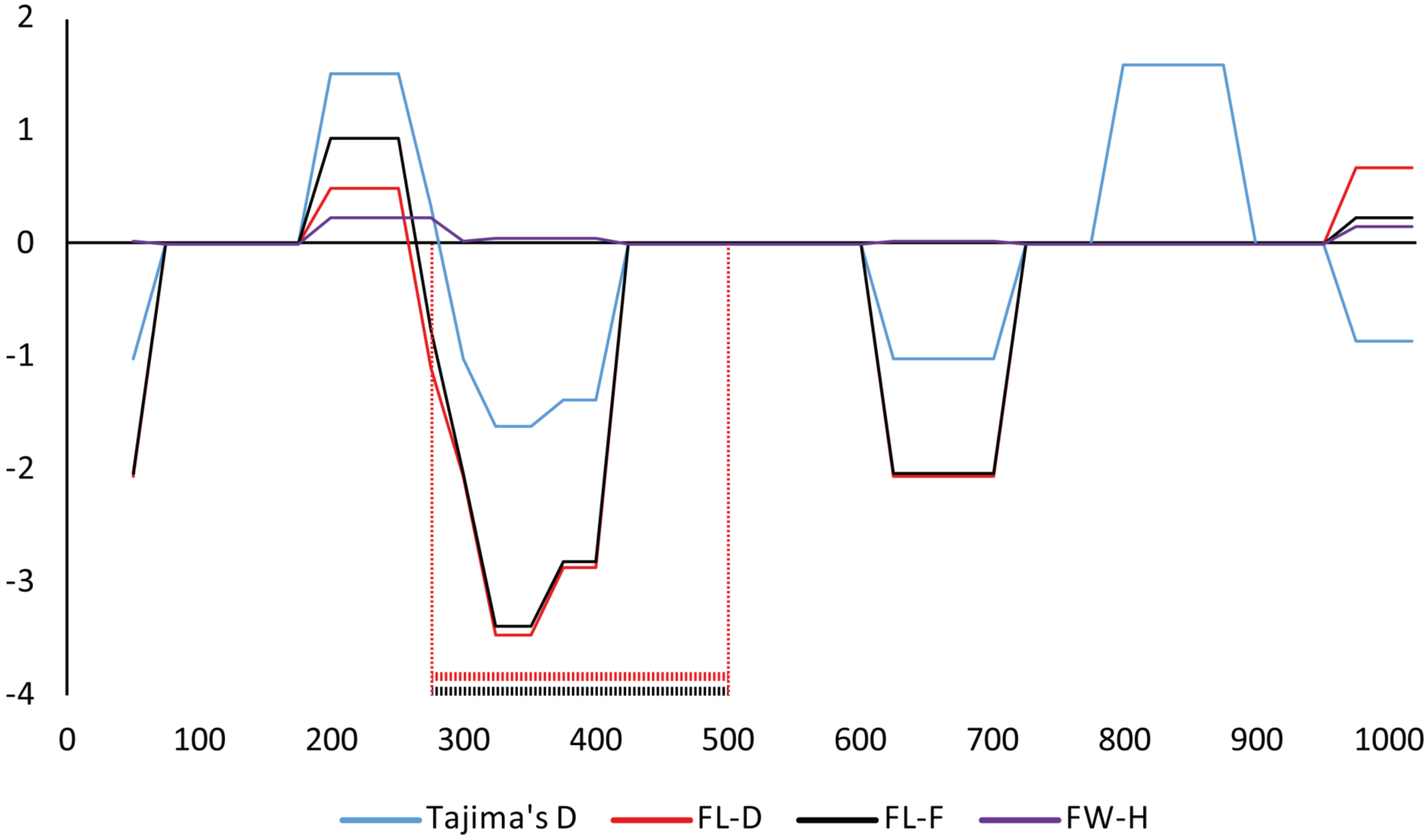
2
1
0
-1
-2
-3
-4
0
100
200
300
400
500
600
700
800
900
1000
Tajima's D
FL-D
FL-F
FW-H

B

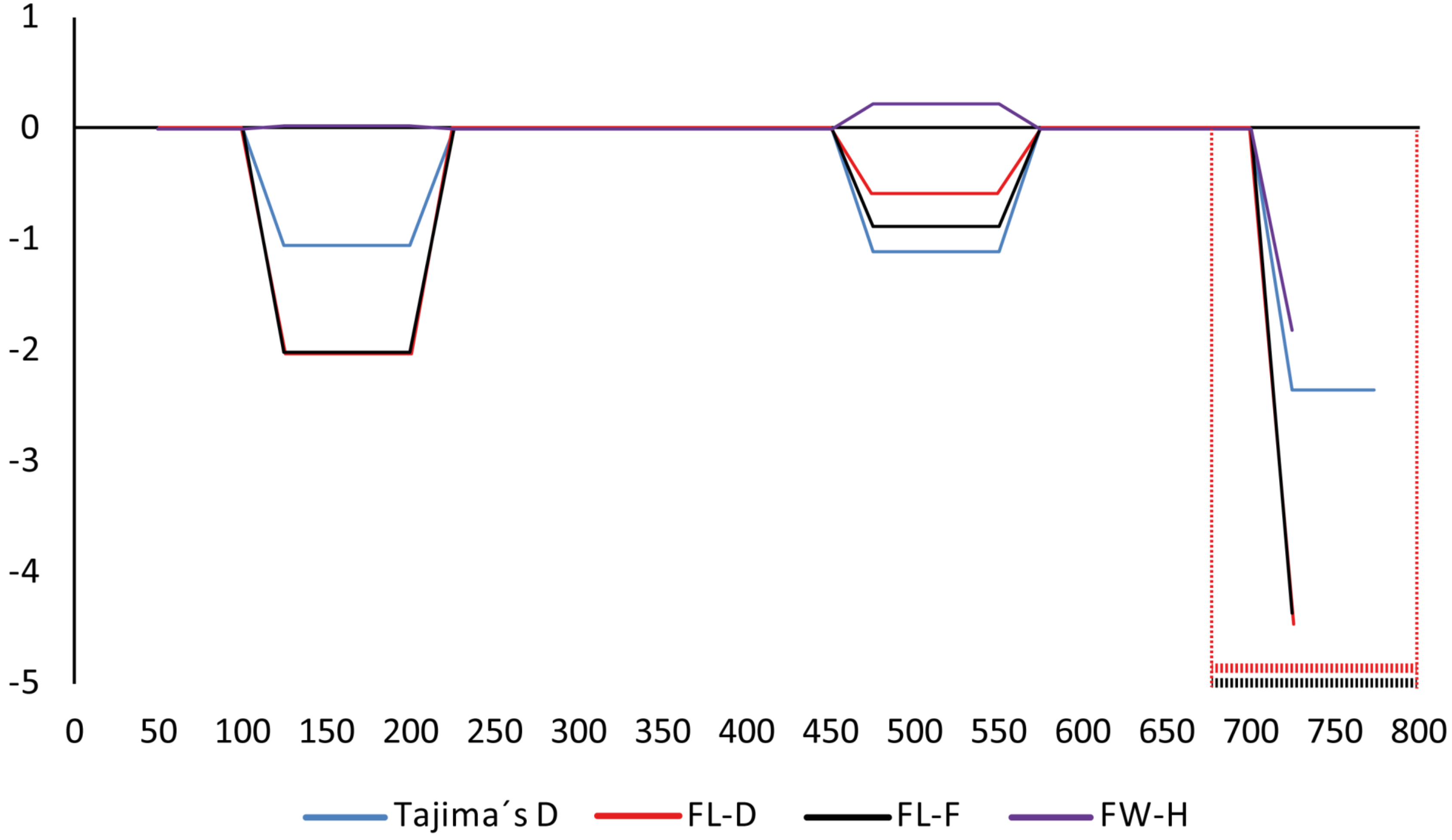
1
0
-1
-2
-3
-4
-5
0
50
100
150
200
250
300
350
400
450
500
550
600
650
700
750
800
Tajima´s D
FL-D
FL-F
FW-H

C

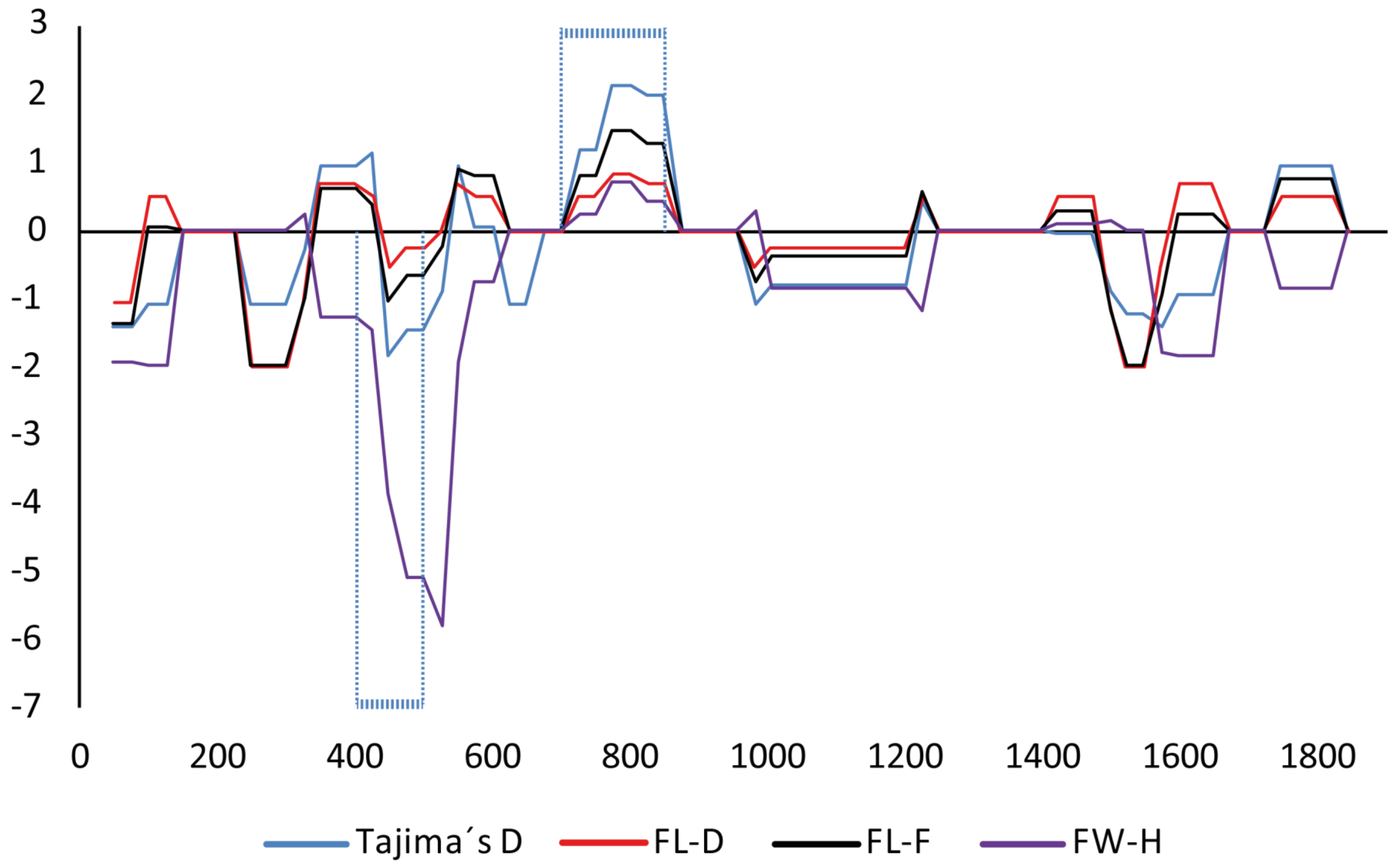
3
2
1
0
-1
-2
-3
-4
-5
-6
-7
0
200
400
600
800
1000
1200
1400
1600
1800
Tajima´s D
FL-D
FL-F
FW-H

D

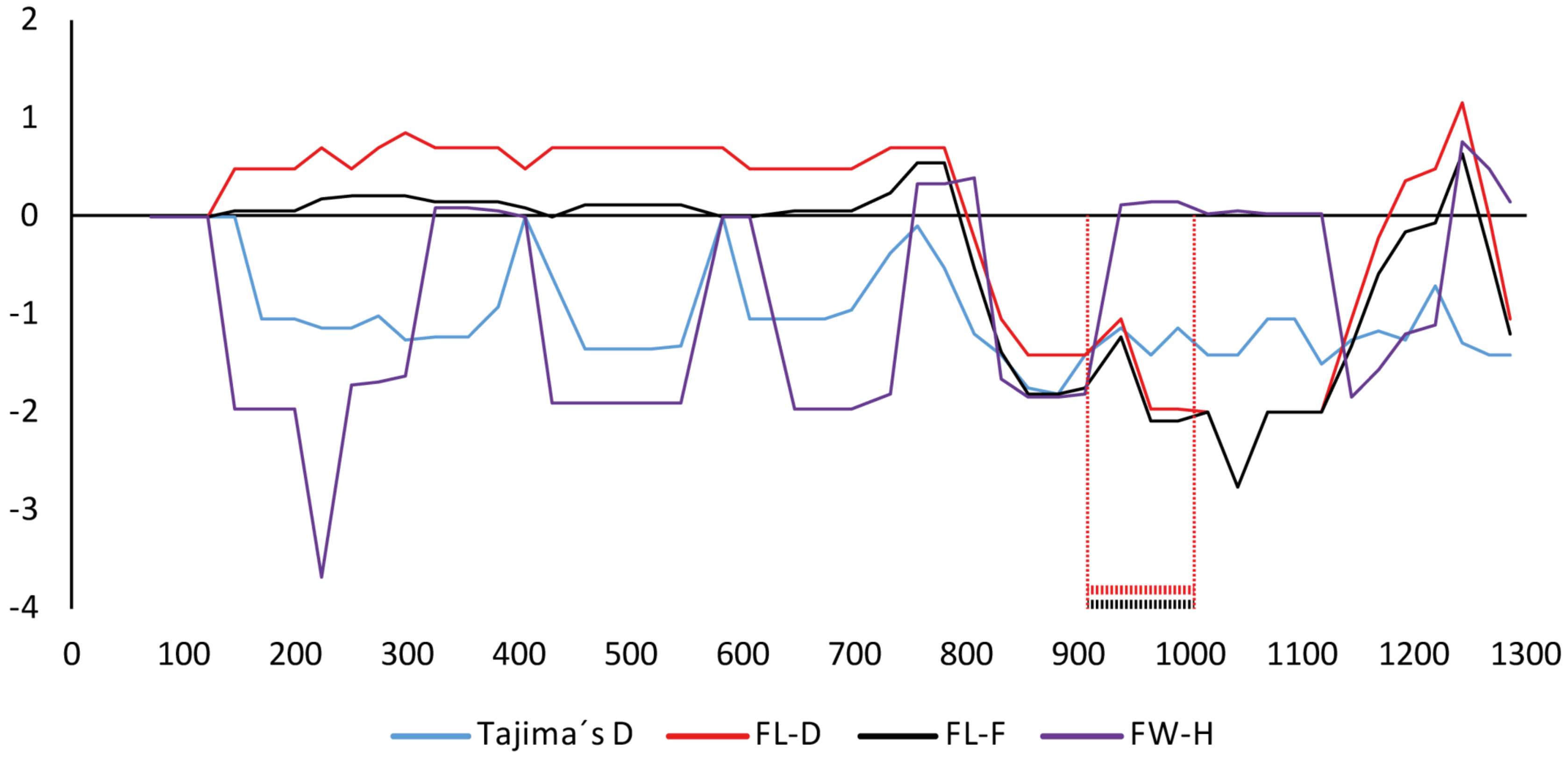
2
1
0
-1
-2
-3
-4
0
100
200
300
400
500
600
700
800
900
1000
1100
1200
1300
Tajima´s D
FL-D
FL-F
FW-H

E

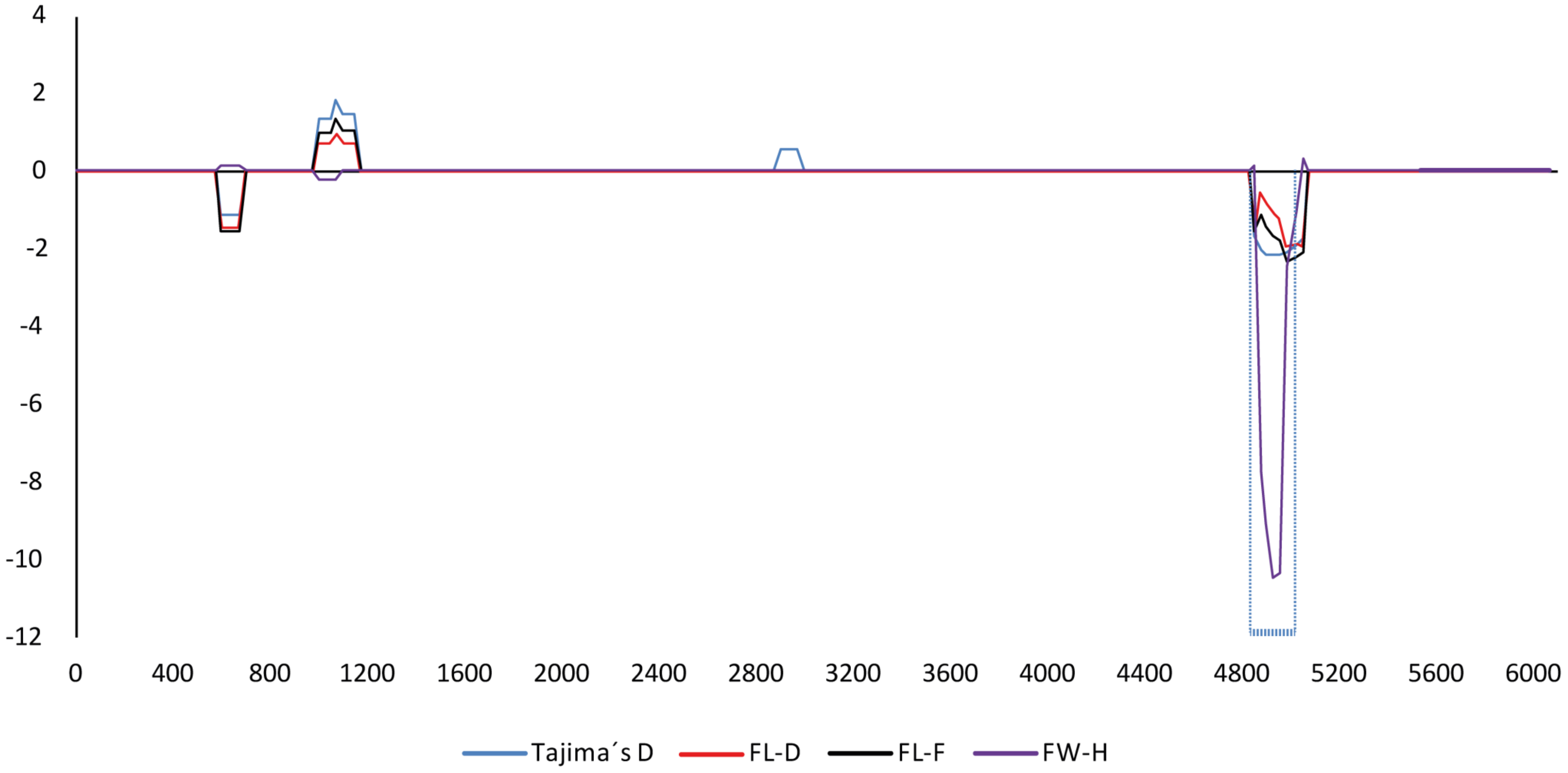
4
2
0
-2
-4
-6
-8
-10
-12
0
400
800
1200
1600
2000
2400
2800
3200
3600
4000
4400
4800
5200
5600
6000
Tajima´s D
FL-D
FL-F
FW-H

F

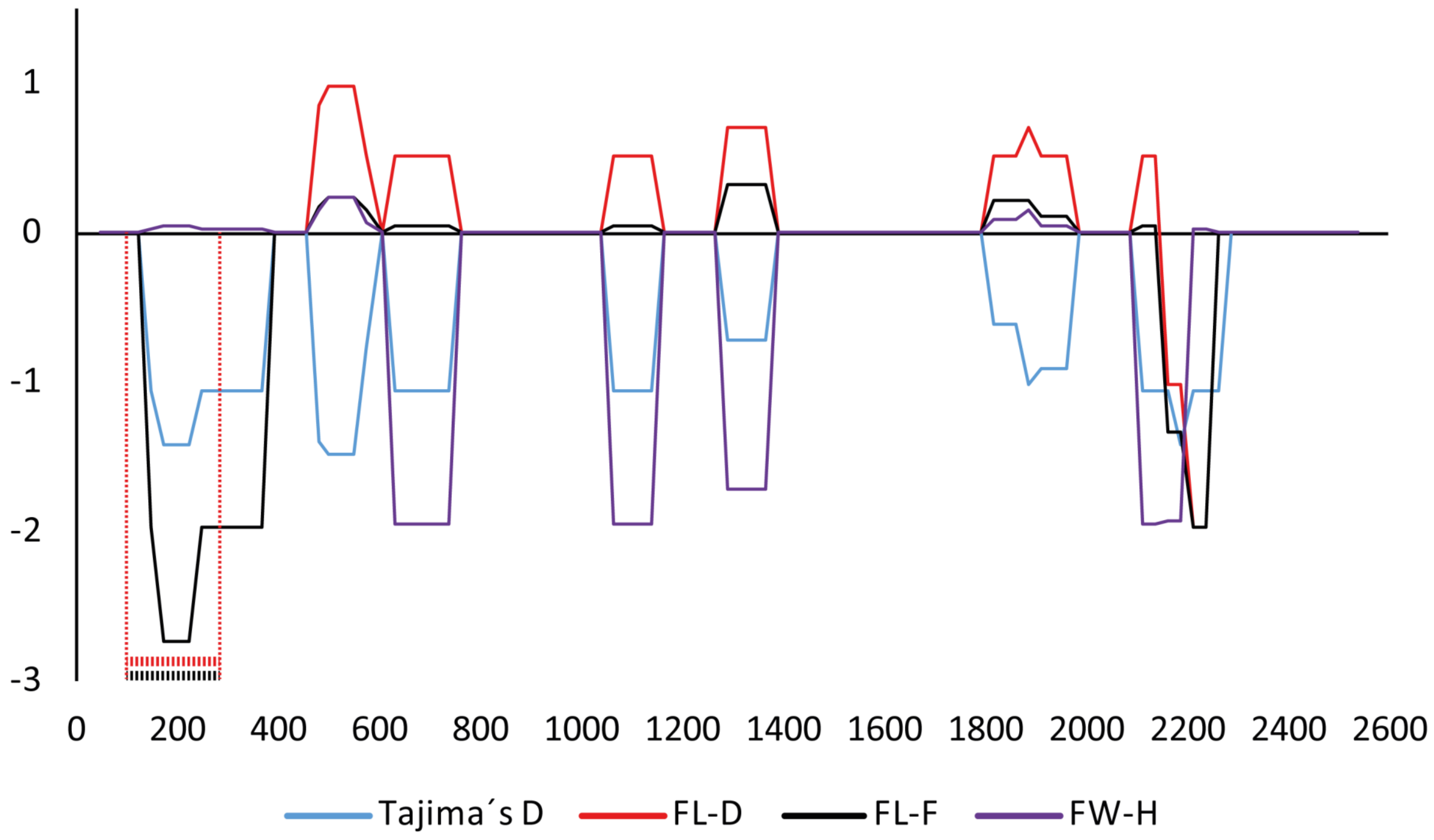
1
0
-1
-2
-3
0
200
400
600
800
1000
1200
1400
1600
1800
2000
2200
2400
2600
Tajima´s D
FL-D
FL-F
FW-H

G

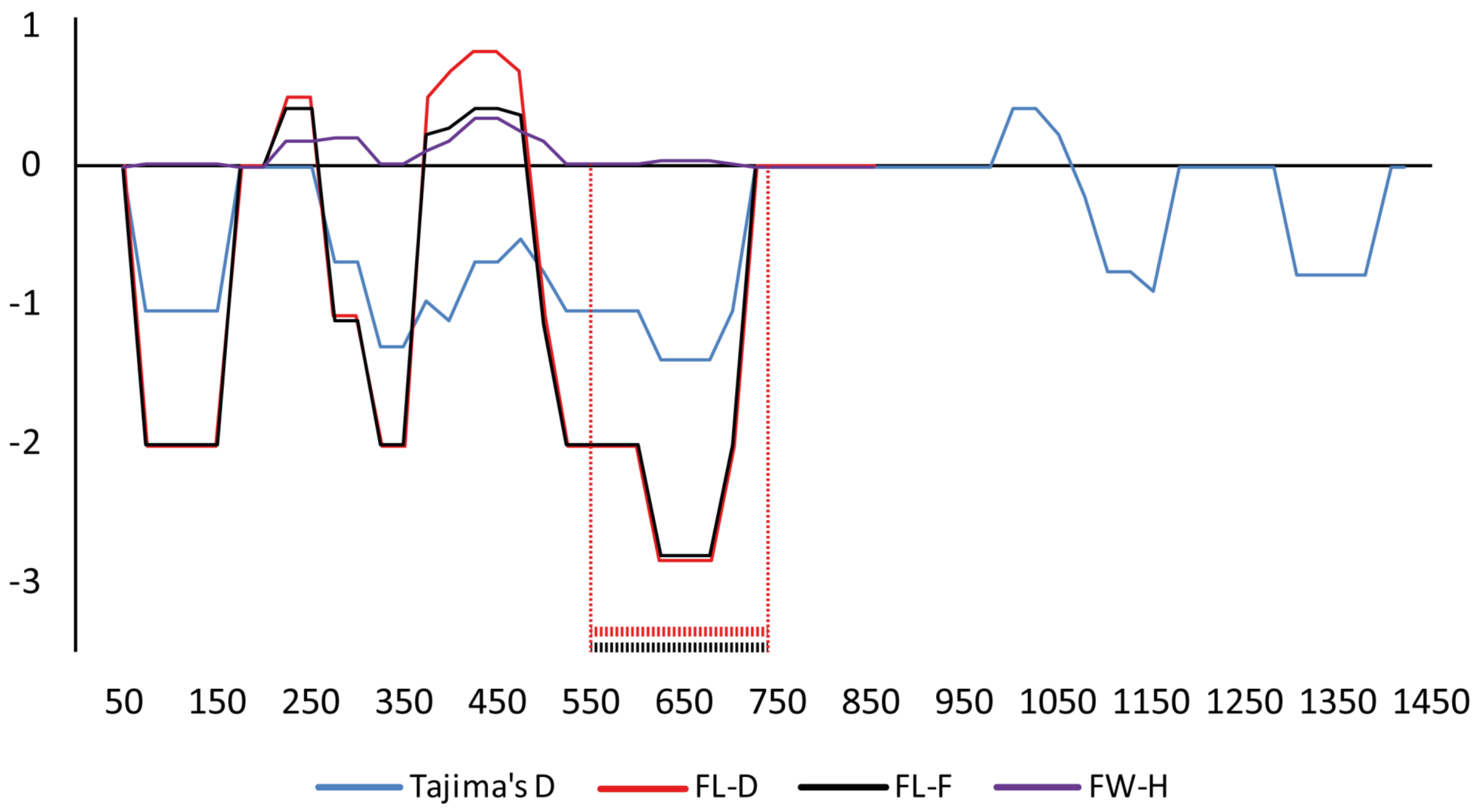
1
0
-1
-2
-3
50
150
250
350
450
550
650
750
850
950
1050
1150
1250
1350
1450
Tajima's D
FL-D
FL-F
FW-H

H

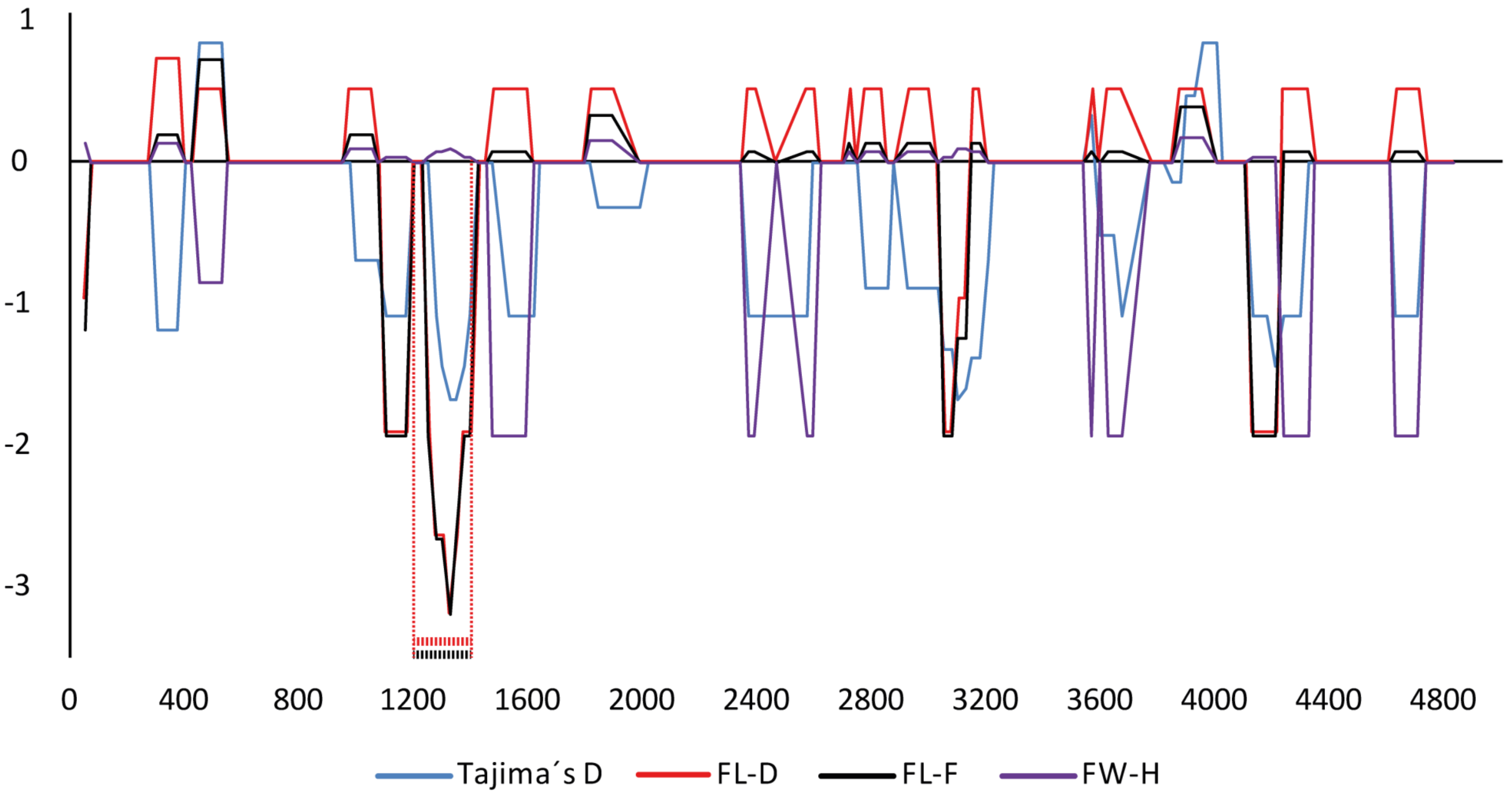

1
0
-1
-2
-3
0
400
800
1200
1600
2000
2400
2800
3200
3600
4000
4400
4800
Tajima´s D
FL-D
FL-F
FW-H

I

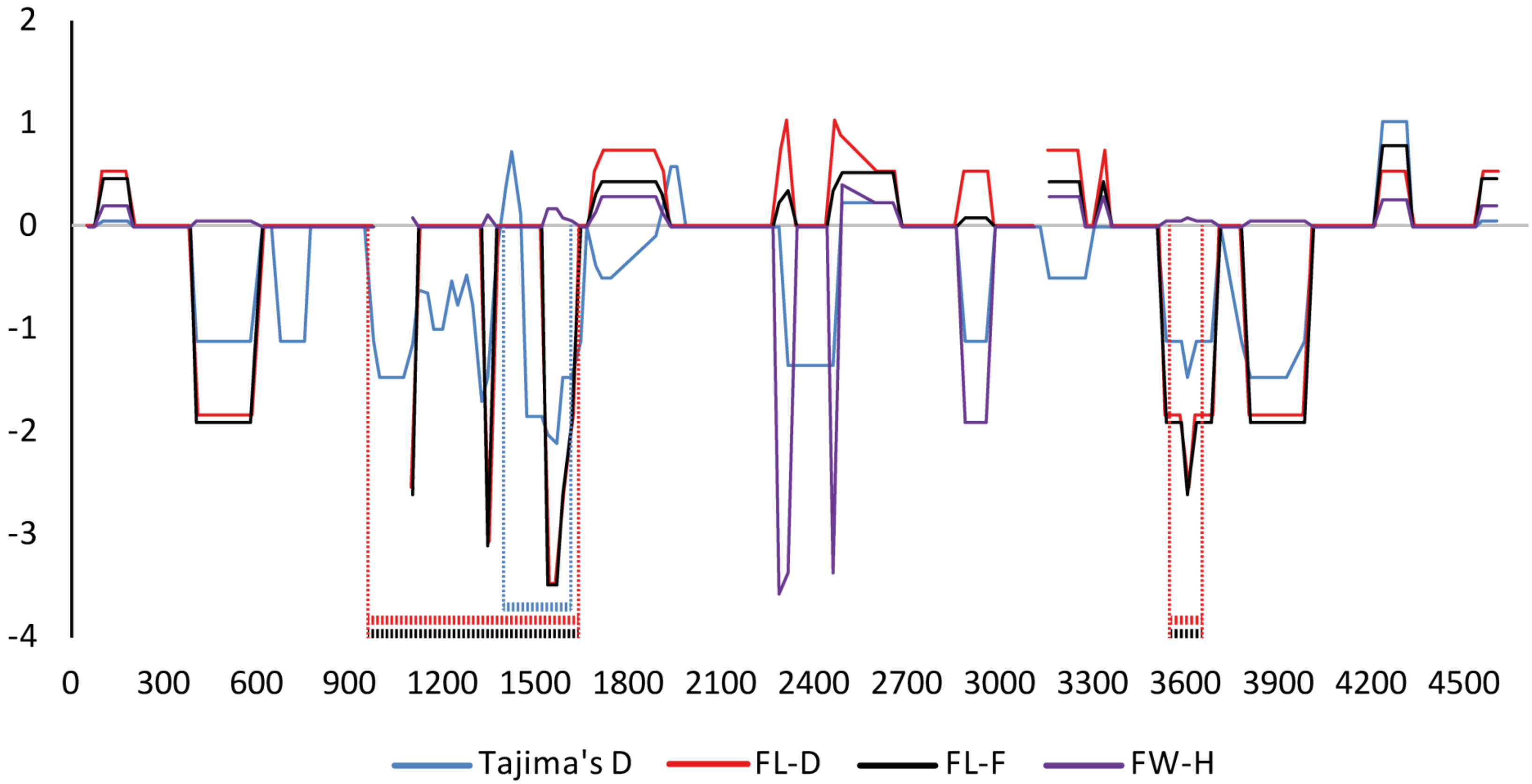
2
1
0
-1
-2
-3
-4
0
300
600
900
1200
1500
1800
2100
2400
2700
3000
3300
3600
3900
4200
4500
Tajima's D
FL-D
FL-F
FW-H

Supplementary Material 1. Sliding window analysis of neutral tests based on polymorphism frequency spectrum. Bars [Tajima's D (blue), Fu & Li's D (red) and F (black) and Fay & Wu (purple)] below each figure represent the regions where the tests revealed significant deviation from neutral expectation. A. *p36* (PVX_001025), B. *spatr* (PVX_002900), C. *trap* (PVX_082735), D. *siap2* (PVX_088860), E. *maebl* (PVX_092975), F. *plp1* (PVX_000810), G. *mcp1* (PVX_111355), H. *tlp* (PVX_113965), I. *mb2* (PVX_080420).
