# Supplementary material for: Identifying Potential Plasmodium vivax Sporozoite Stage Vaccine Candidates: An Analysis of Genetic Diversity and Natural Selection
Source: Front Genet. 2018 Jan 25;9:10. doi: 10.3389/fgene.2018.00010 (PMC5788960; doi:10.3389/fgene.2018.00010)
Supplement: Supplementary file 2 [file Presentation2.PDF]

Supplementary Material 2. Sliding window analysis for ω rates (d_N/d_S and/or K_N/K_S) regarding Plasmodium vivax sporozoite genes

Identifying potential *P. vivax* sporozoite stage vaccine candidates: an analysis of genetic diversity and natural selection

Diego Garzón-Ospina, Sindy Paola Buitrago, Andrea Estefania Ramos, Manuel A. Patarroyo*

*** Correspondence:** mapatarr.fidic@gmail.com

A

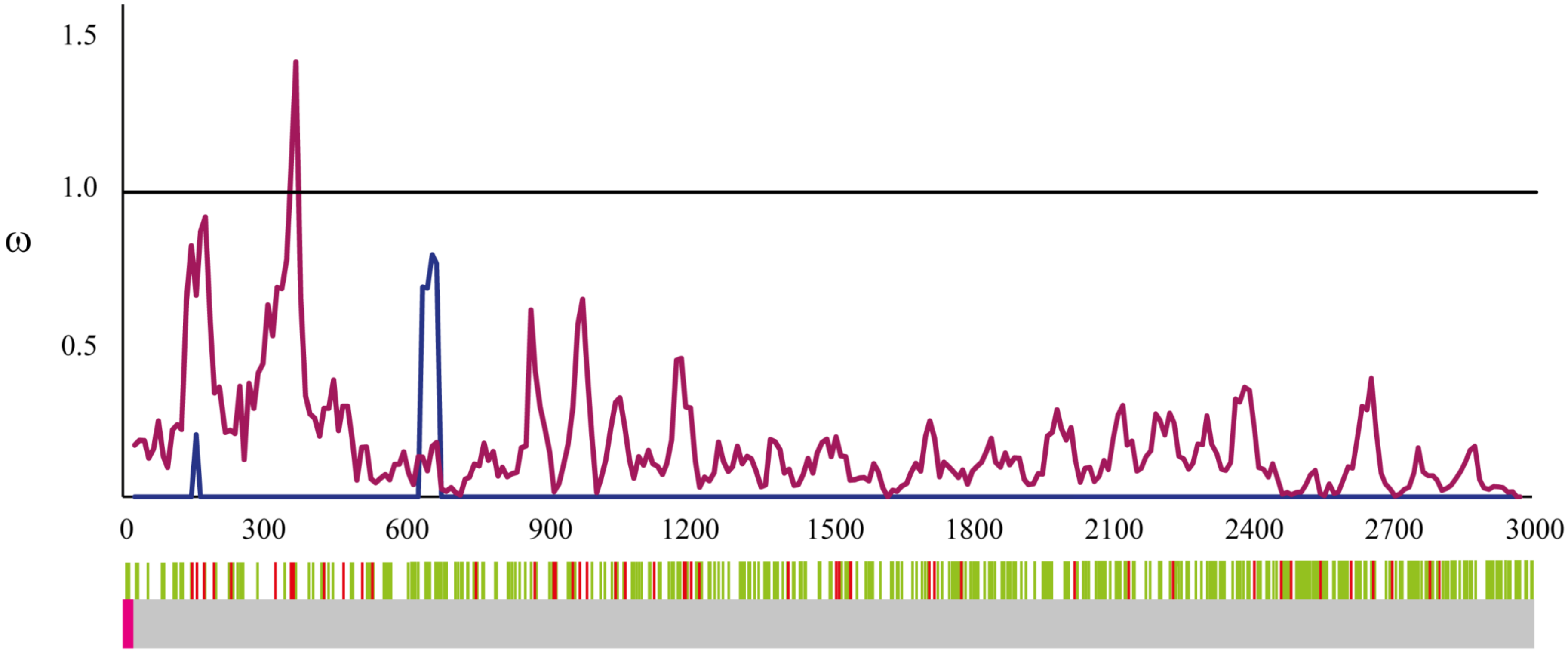

B

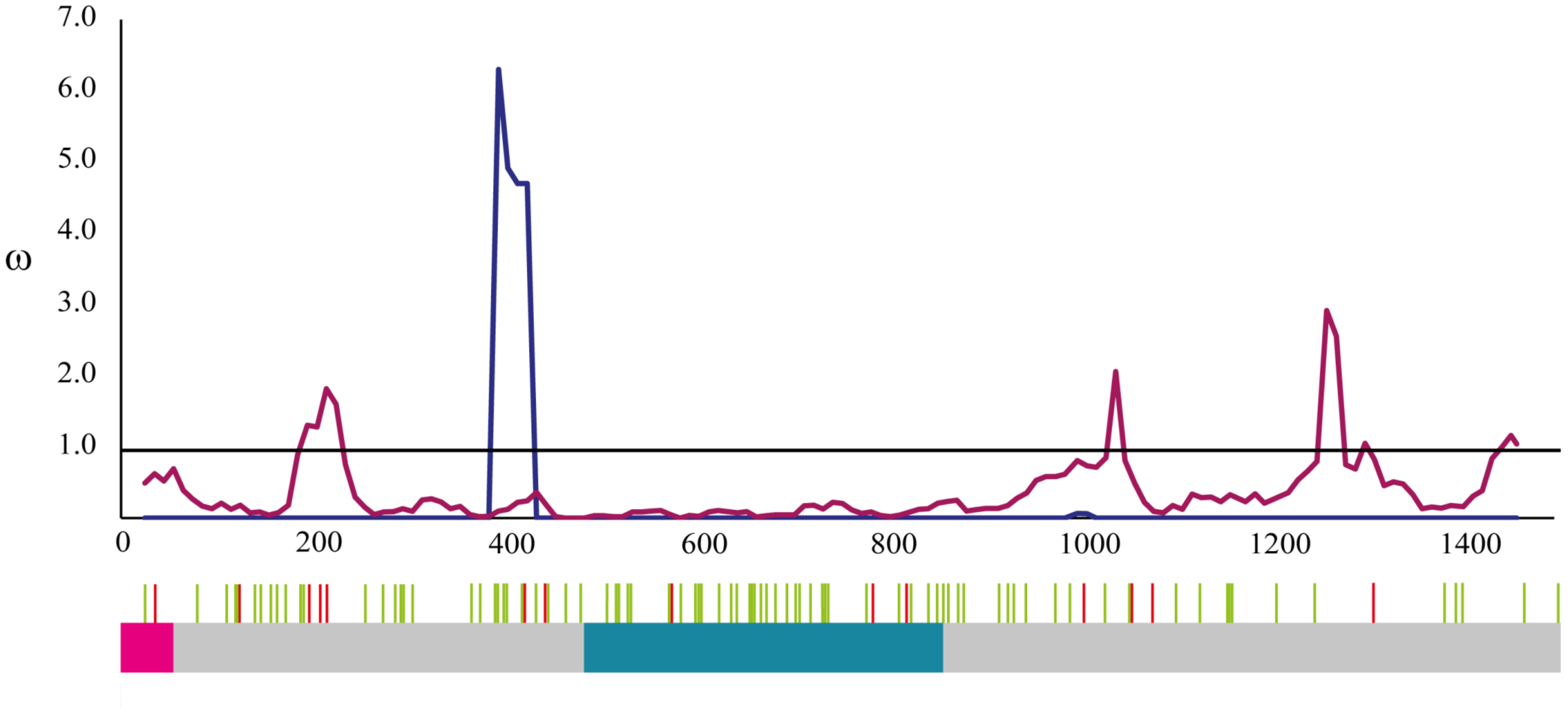

C

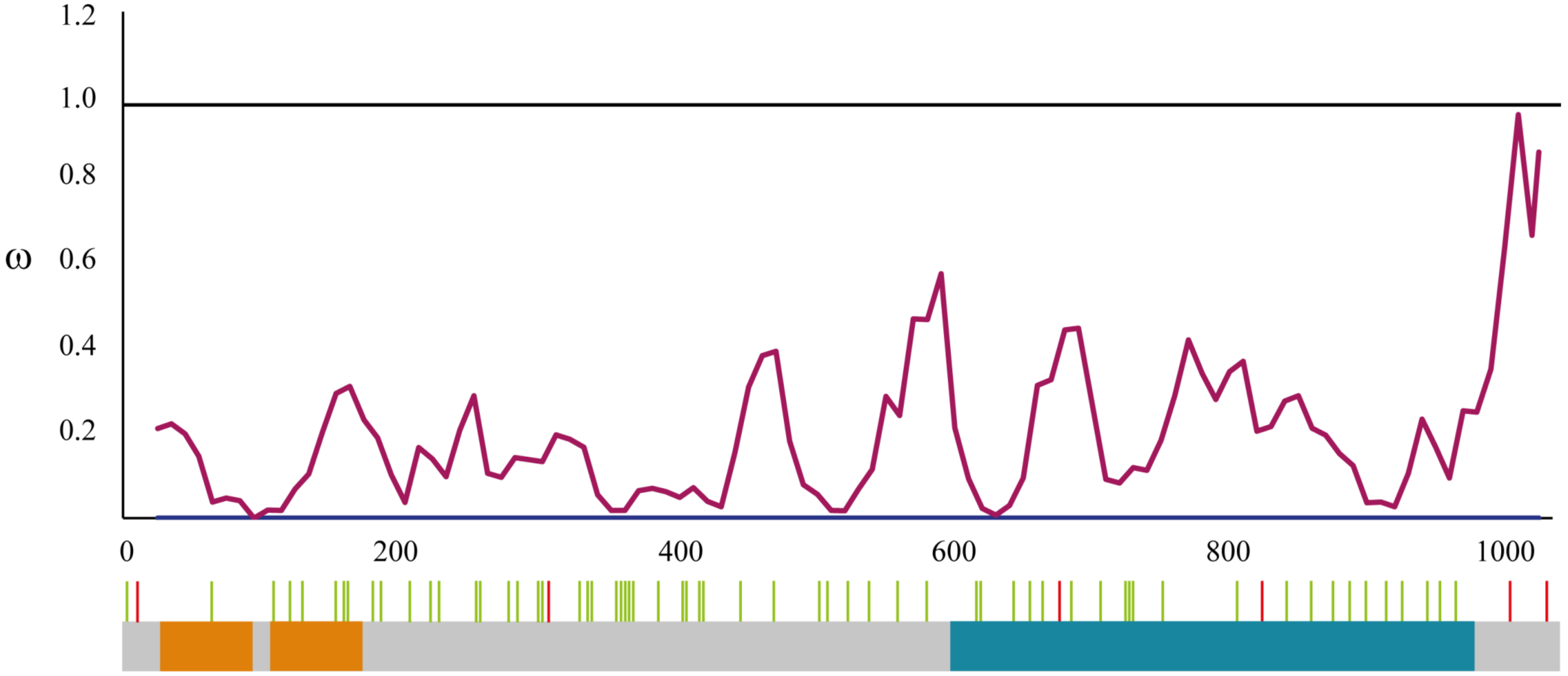

D

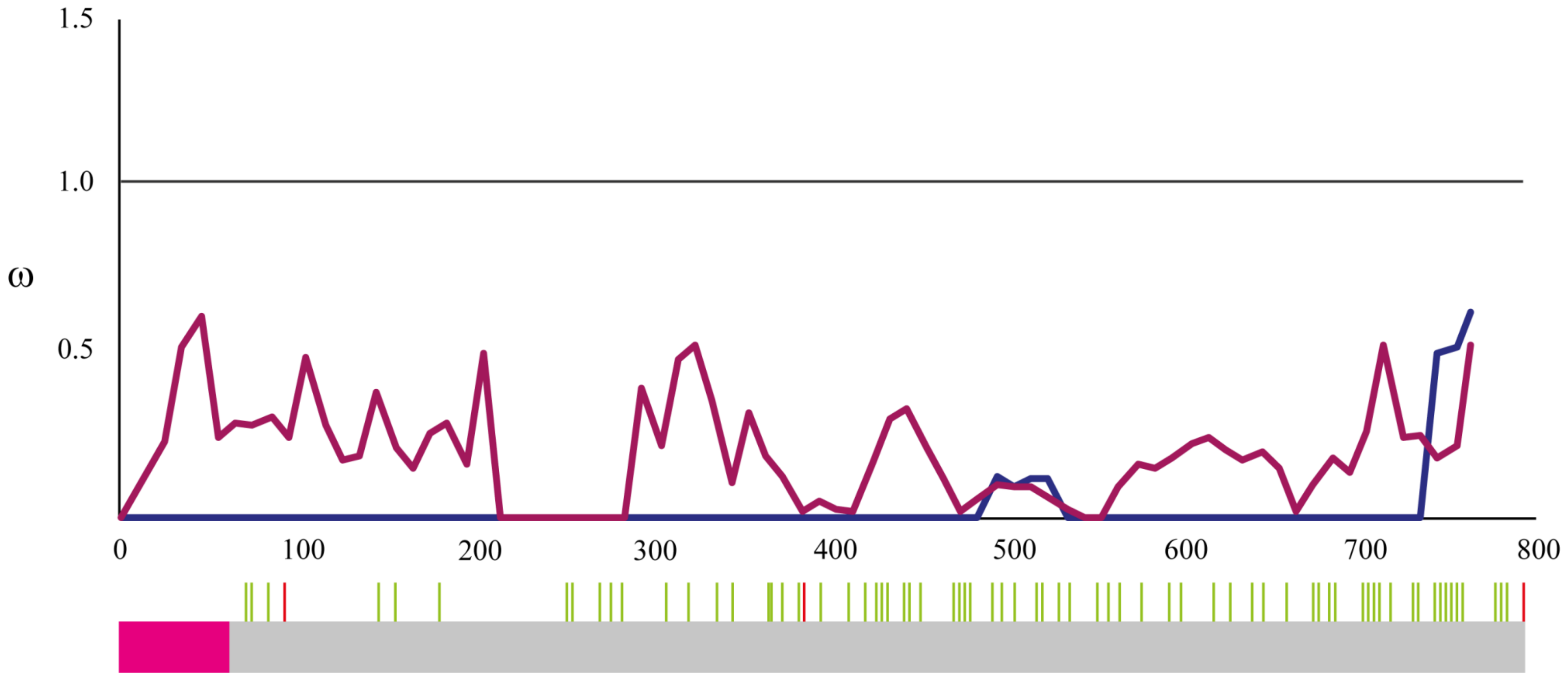

E

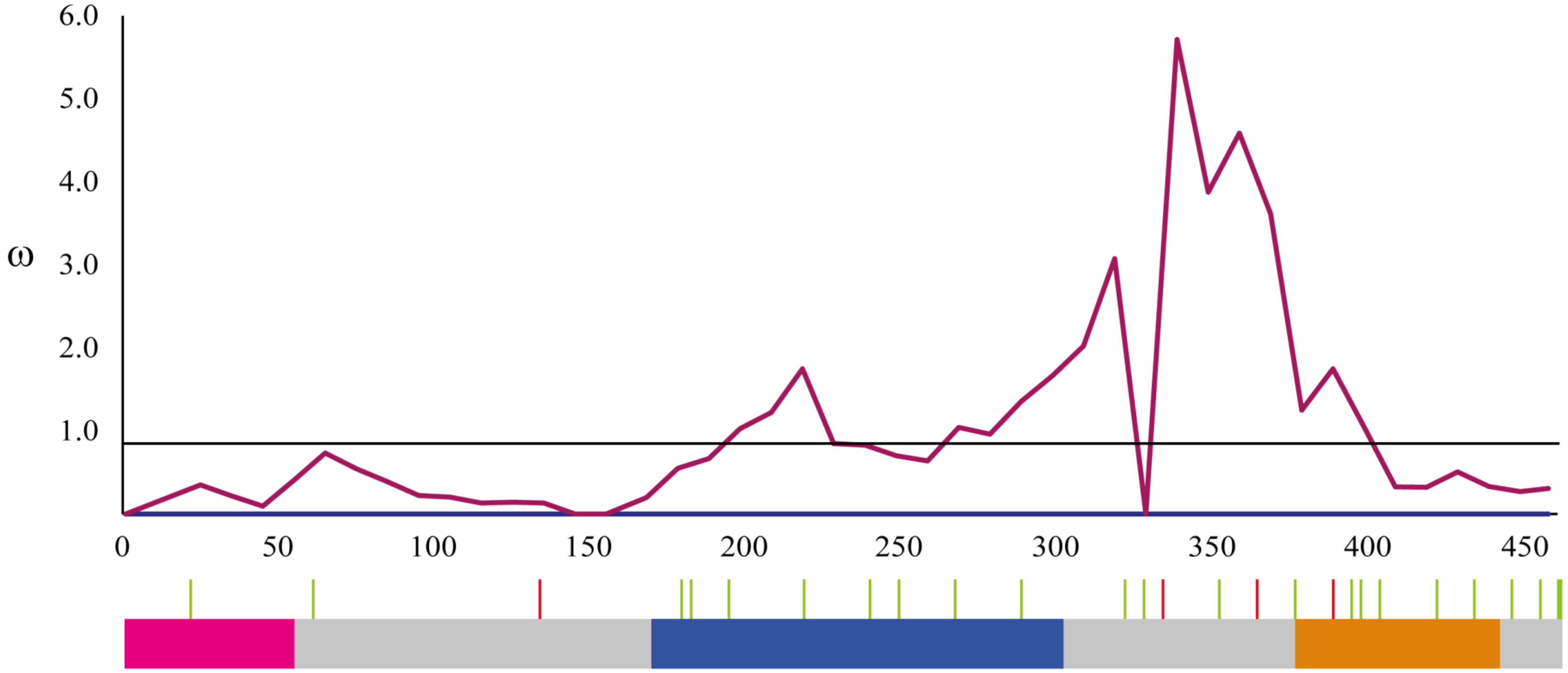

F

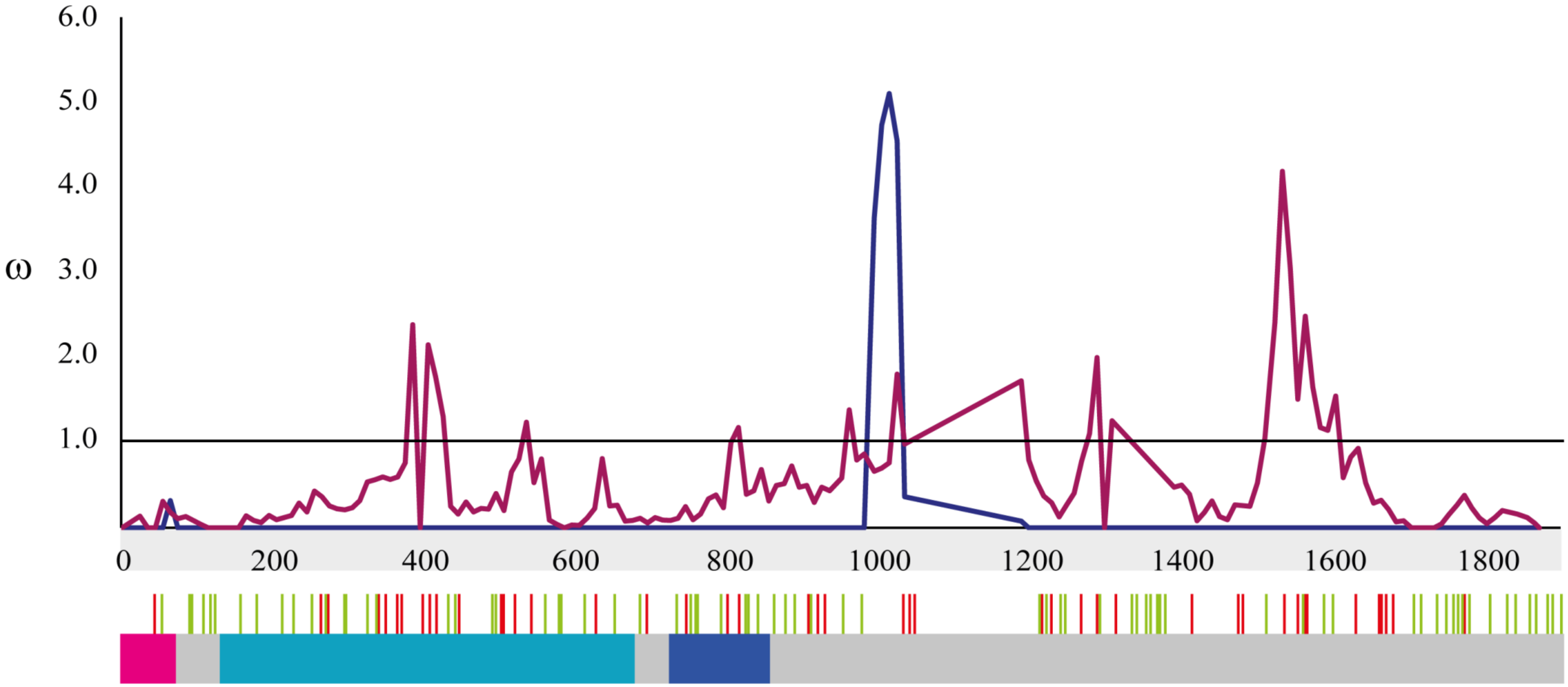

G

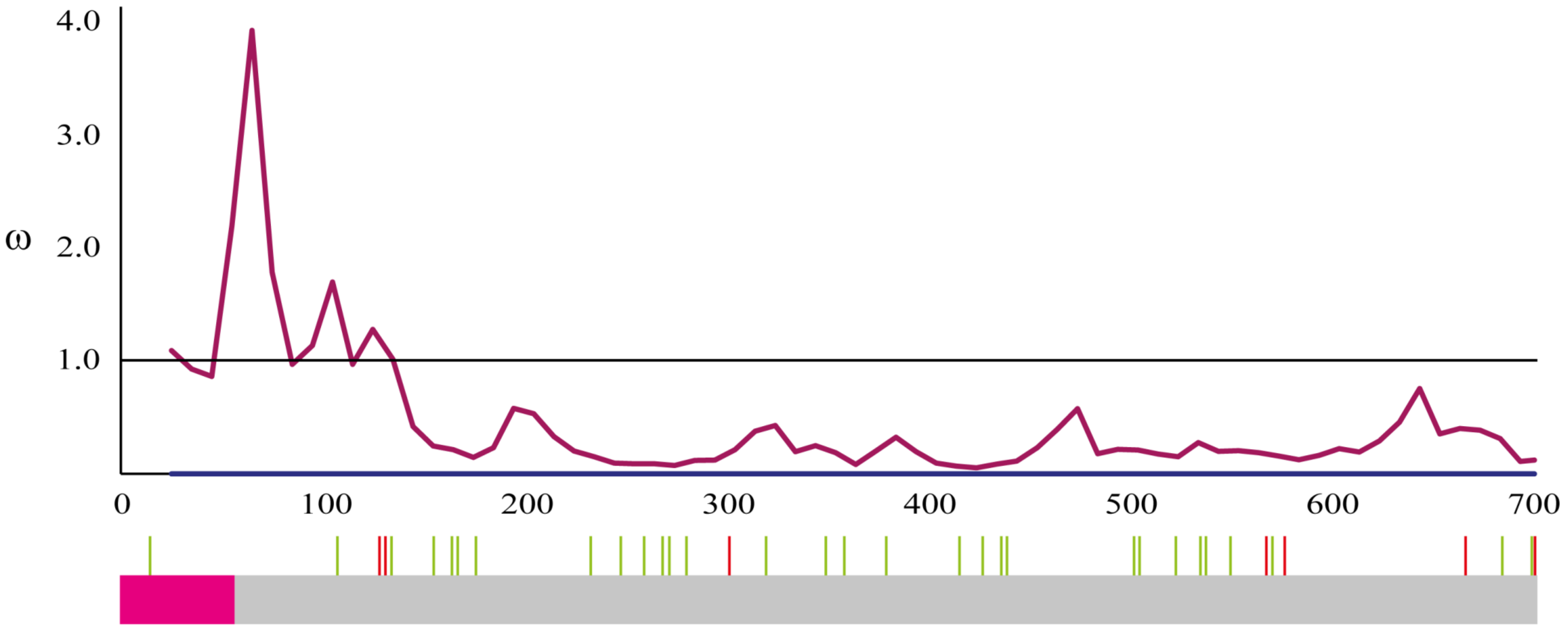

H

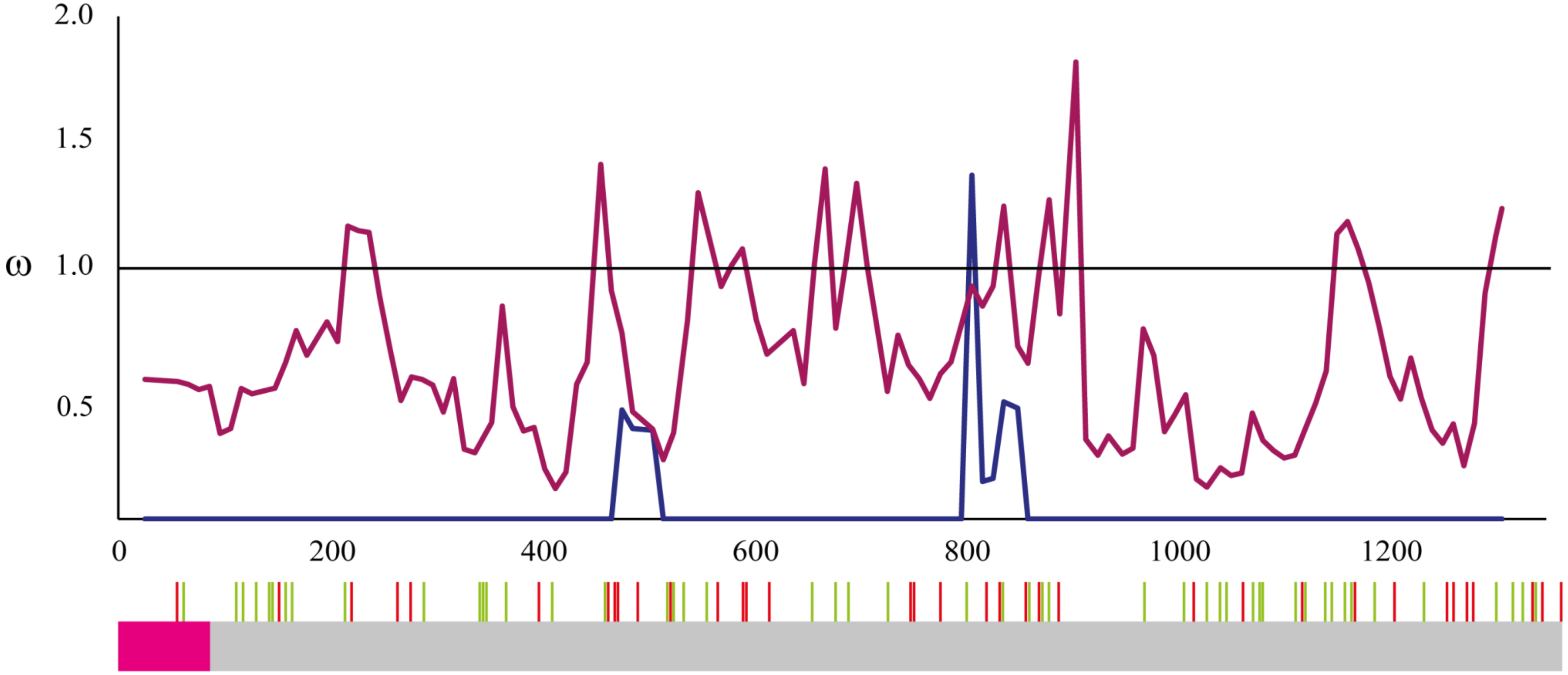

I

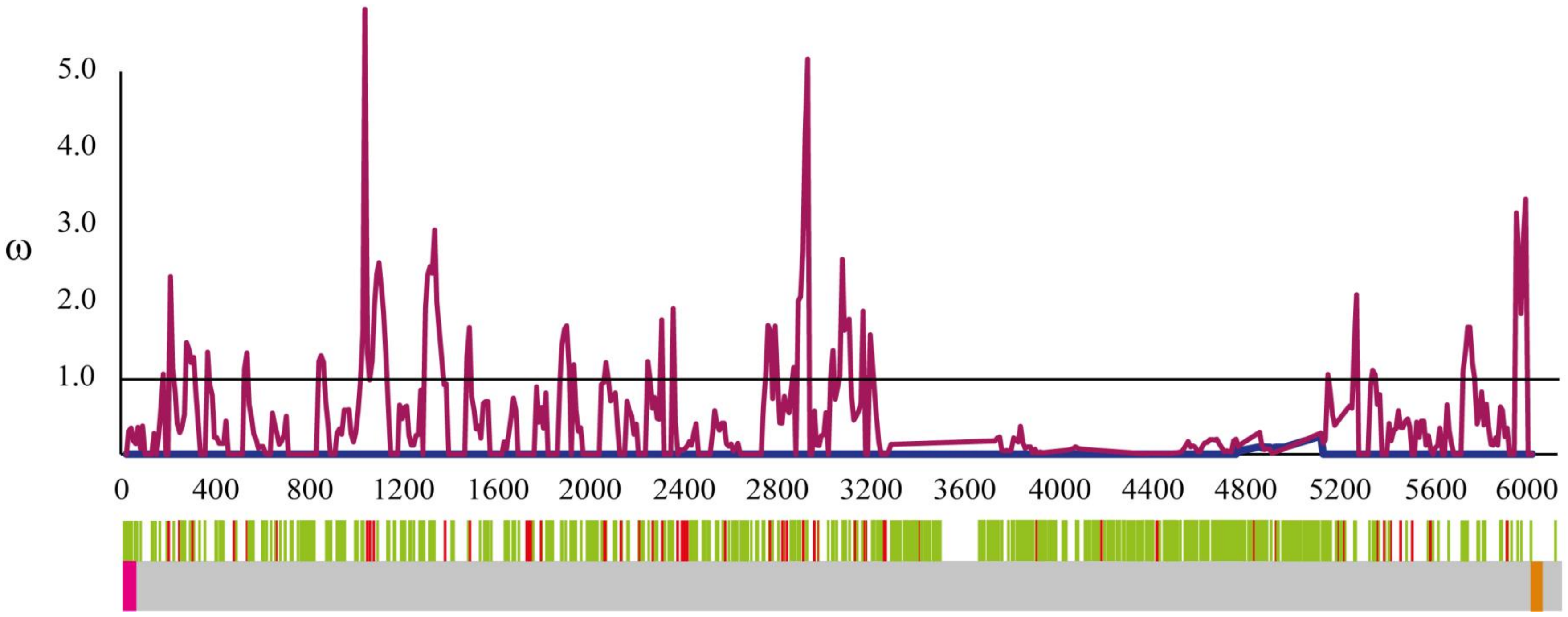
5.0
4.0
3.0
2.0
1.0
ω
0
400
800
1200
1600
2000
2400
2800
3200
3600
4000
4400
4800
5200
5600
6000

J

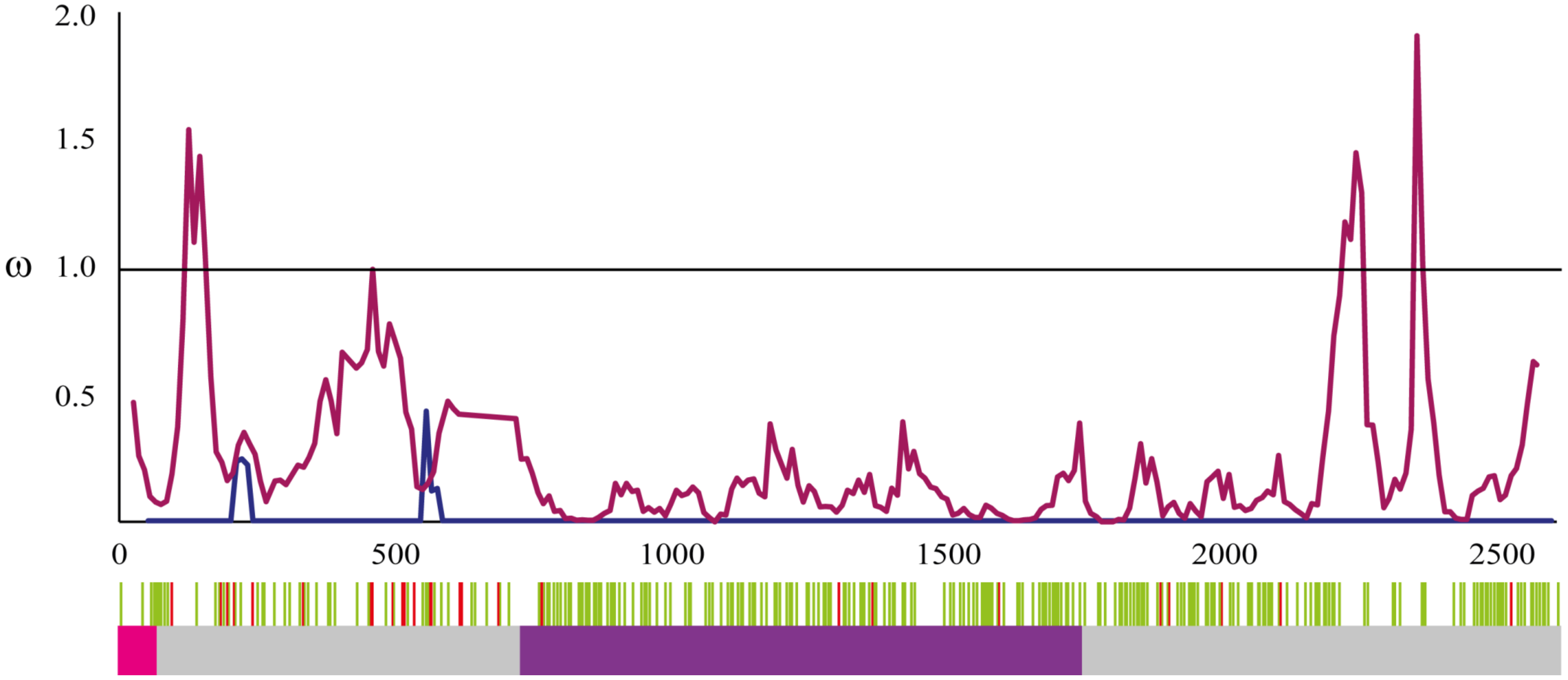

K

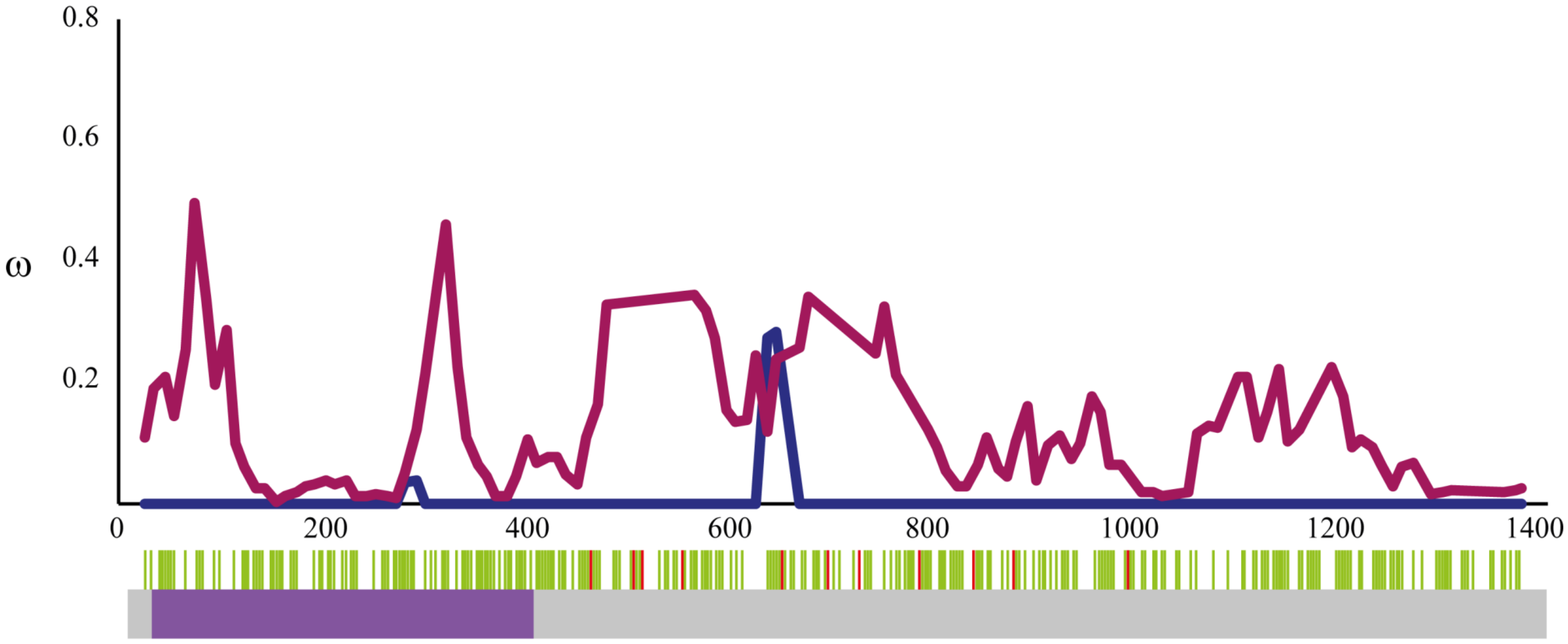

L

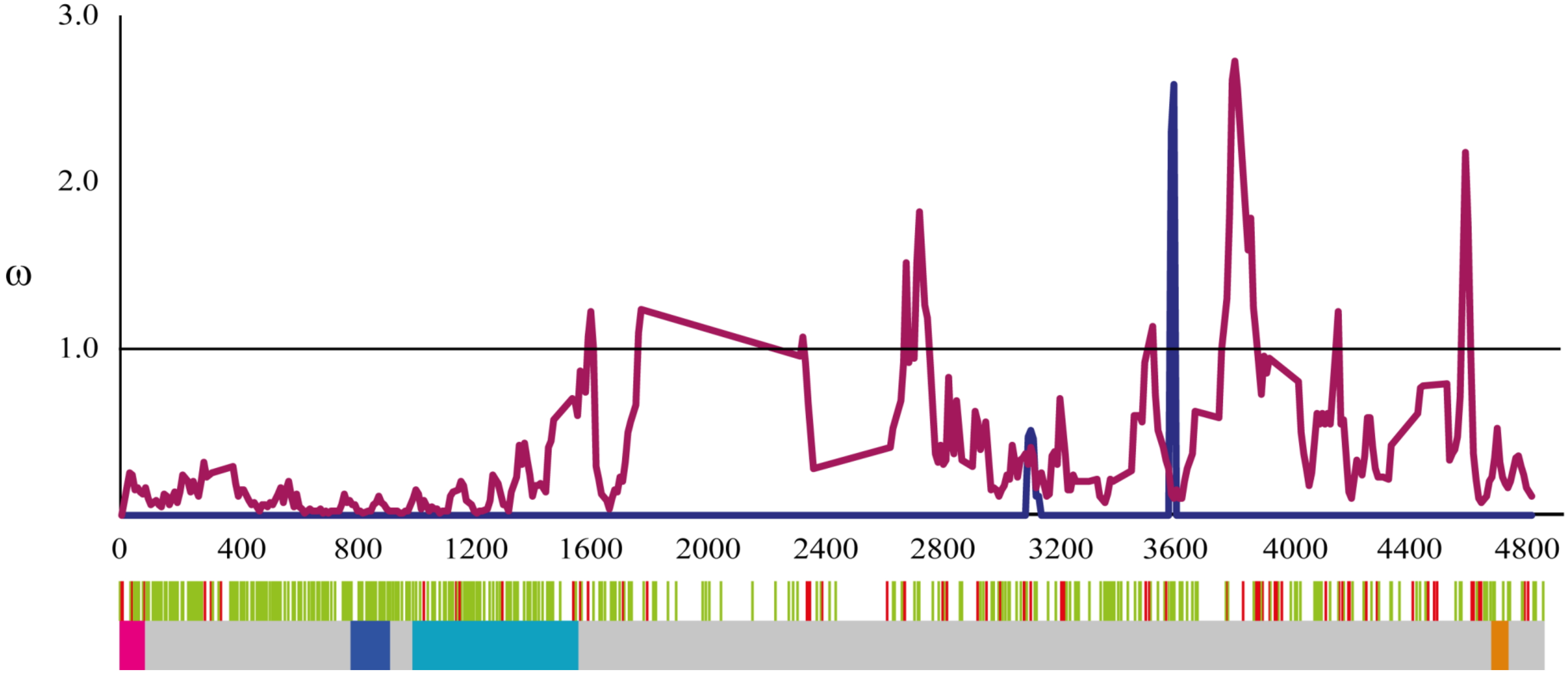

3.0
2.0
ω
1.0
0
400
800
1200
1600
2000
2400
2800
3200
3600
4000
4400
4800

M

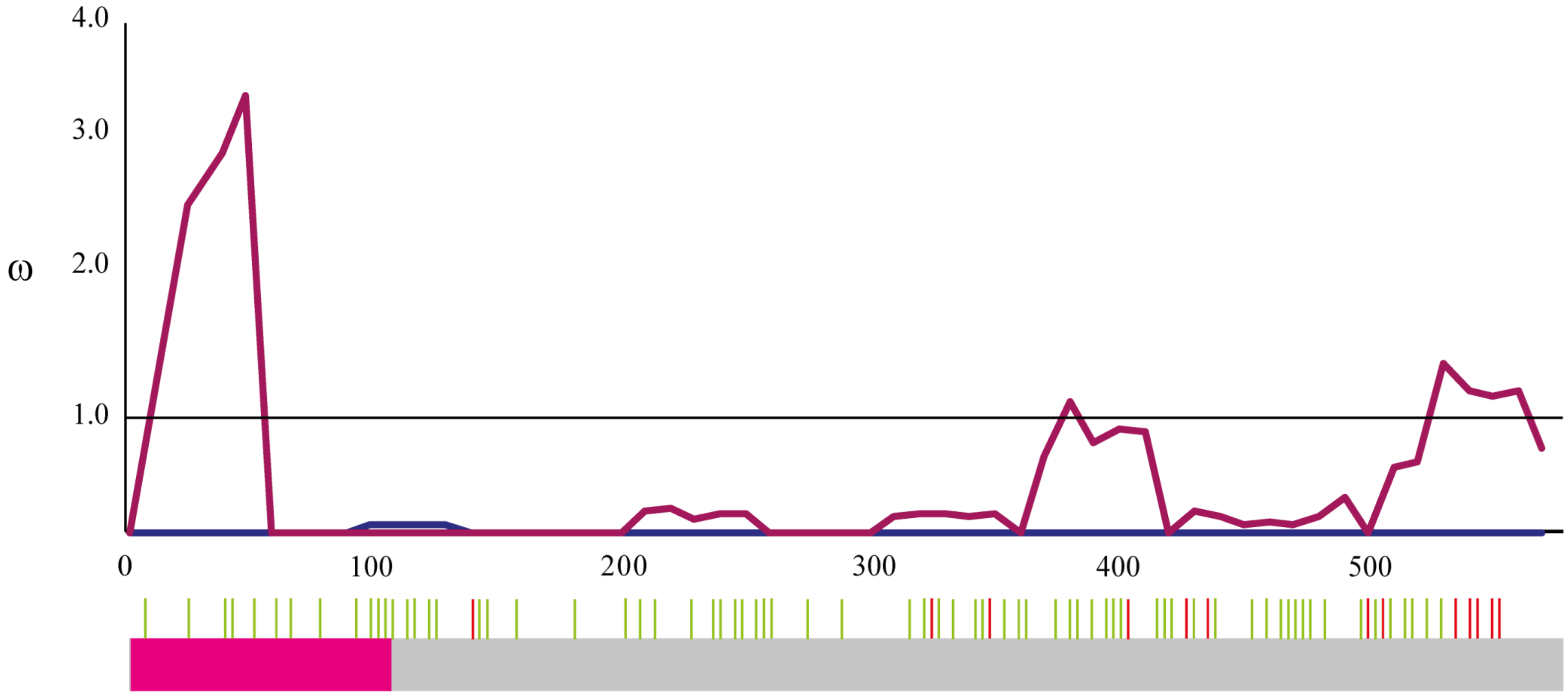

N

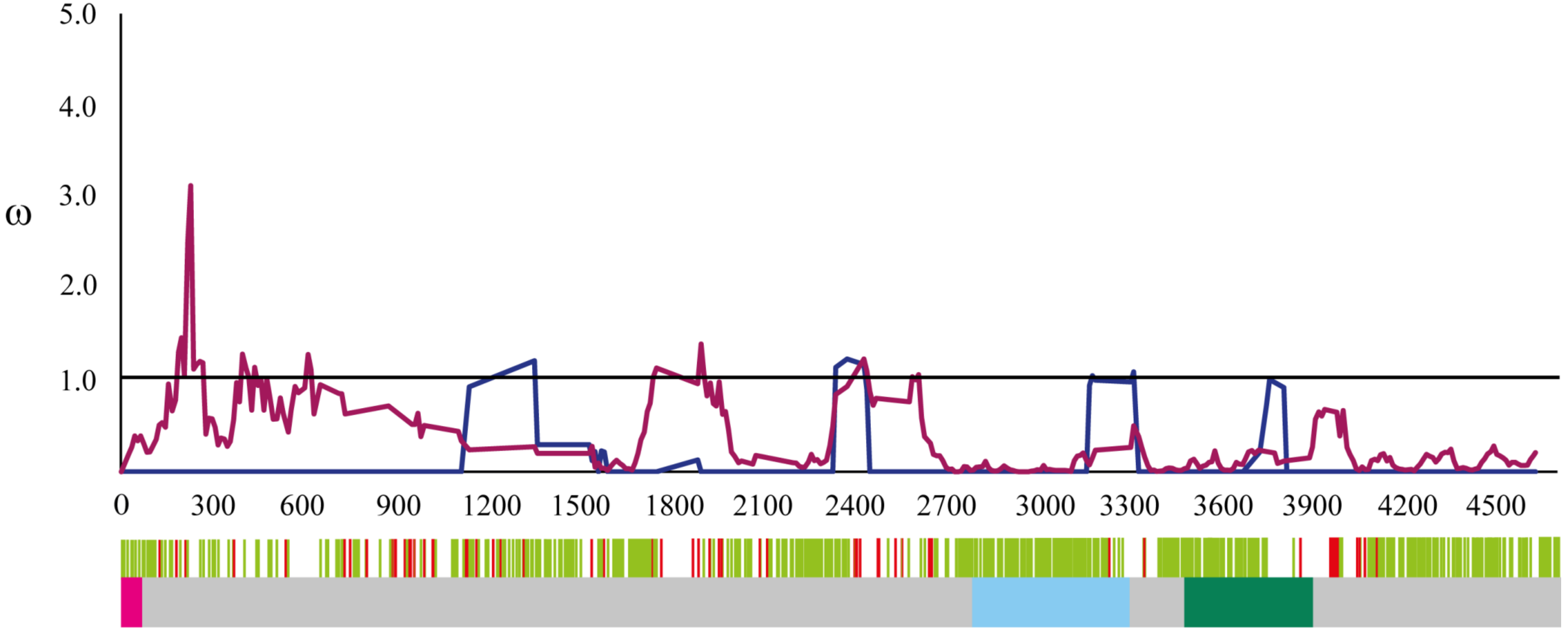

ω
5.0
4.0
3.0
2.0
1.0
0
300
600
900
1200
1500
1800
2100
2400
2700
3000
3300
3600
3900
4200
4500

Supplementary Material 2. Sliding window analysis for ω rates (d_N/d_S and/or K_N/K_S) regarding *Plasmodium vivax* sporozoite genes. *P. vivax* ω values (d_N/d_S) are shown in blue throughout; ω divergence rates (K_N/K_S) between *P. vivax* and phylogenetic-related species are shown in purple. A representation of each gene is given below the sliding window indicating signal peptide (fuchsia), GPI or transmembrane helix (orange) and putative domains (see the Fig. 1 in the main text for colour conventions). The sites under purifying selection between species are represented by light green lines while positively selected sites between species are shown by red lines. A. *siap1* (PVX_000815), B. *p52* (PVX_001020), C. *p36* (PVX_001025), D. *spatr* (PVX_002900), E. *trsp* (PVX_081560), F. *trap* (PVX_082735), G. *spect1* (PVX_083025), H. *siap2* (PVX_088860), I. *maebl* (PVX_092975), J. *plp1* (PVX_000810), K. *mcp1* (PVX_111355), L. *tlp* (PVX_113965), M. *celtos* (PVX_123510), L. *mb2* PVX_080420).
