# Supplementary material for: Identifying Potential Plasmodium vivax Sporozoite Stage Vaccine Candidates: An Analysis of Genetic Diversity and Natural Selection
Source: Front Genet. 2018 Jan 25;9:10. doi: 10.3389/fgene.2018.00010 (PMC5788960; doi:10.3389/fgene.2018.00010)
Supplement: Supplementary file 3 [file Presentation3.PDF]

Supplementary Material 3. Phylogenies analysed for episodic selection.

Identifying potential *P. vivax* sporozoite stage vaccine candidates: an analysis of genetic diversity and natural selection

Diego Garzón-Ospina, Sindy Paola Buitrago, Andrea Estefania Ramos, Manuel A. Patarroyo*

*** Correspondence:** mapatarr.fidic@gmail.com

A

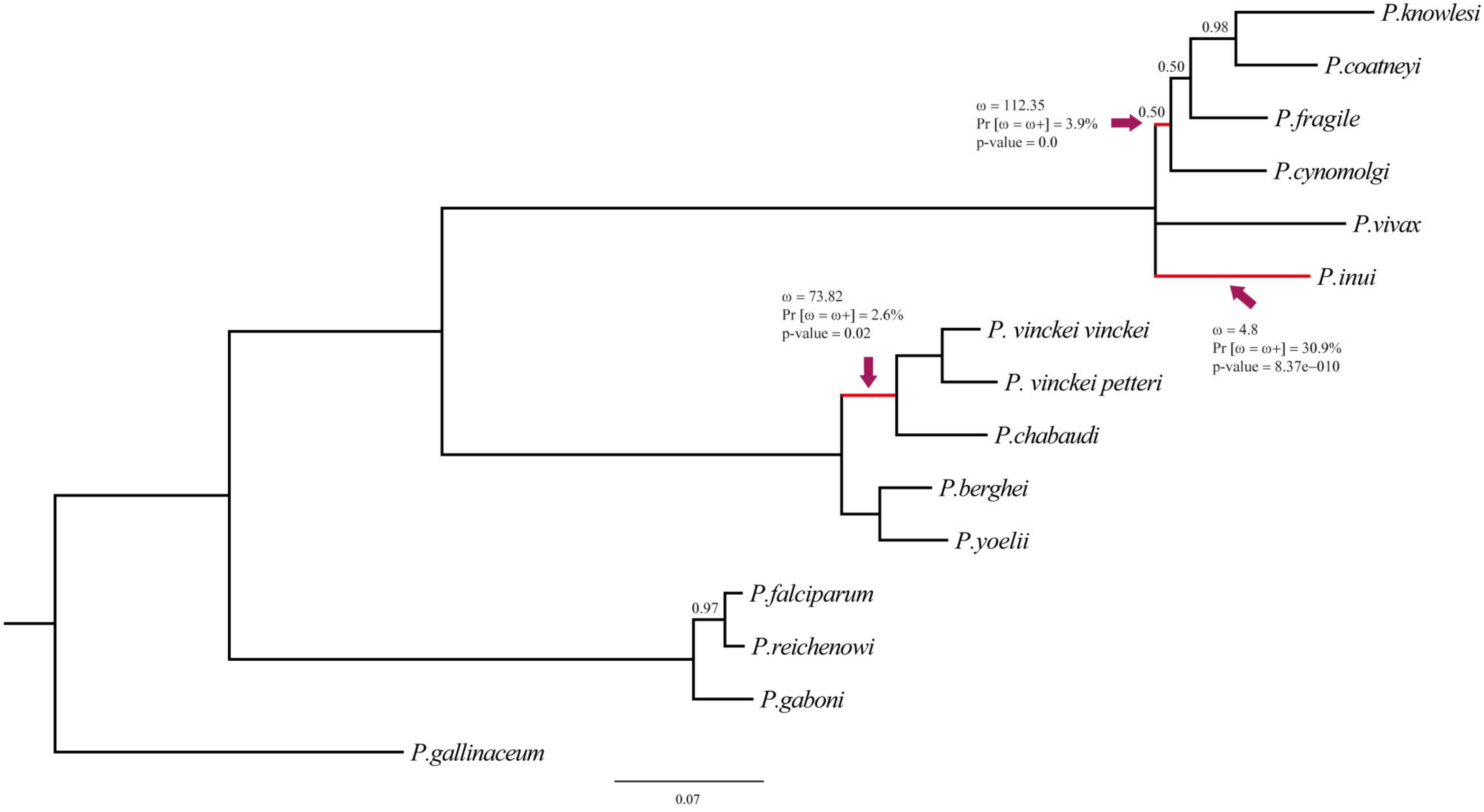
P.knowlesi
0.98
P.coatneyi
0.50
P.fragile
ω = 112.35
Pr [ω = ω+] = 3.9%
p-value = 0.0
0.50
P.cynomolgi
P.vivax
P.inui
ω = 4.8
Pr [ω = ω+] = 30.9%
p-value = 8.37e–010
ω = 73.82
Pr [ω = ω+] = 2.6%
p-value = 0.02
P. vinckei vinckei
P. vinckei petteri
P.chabaudi
P.berghei
P.yoelii
0.97
P.falciparum
P.reichenowi
P.gaboni
P.gallinaceum
0.07

B

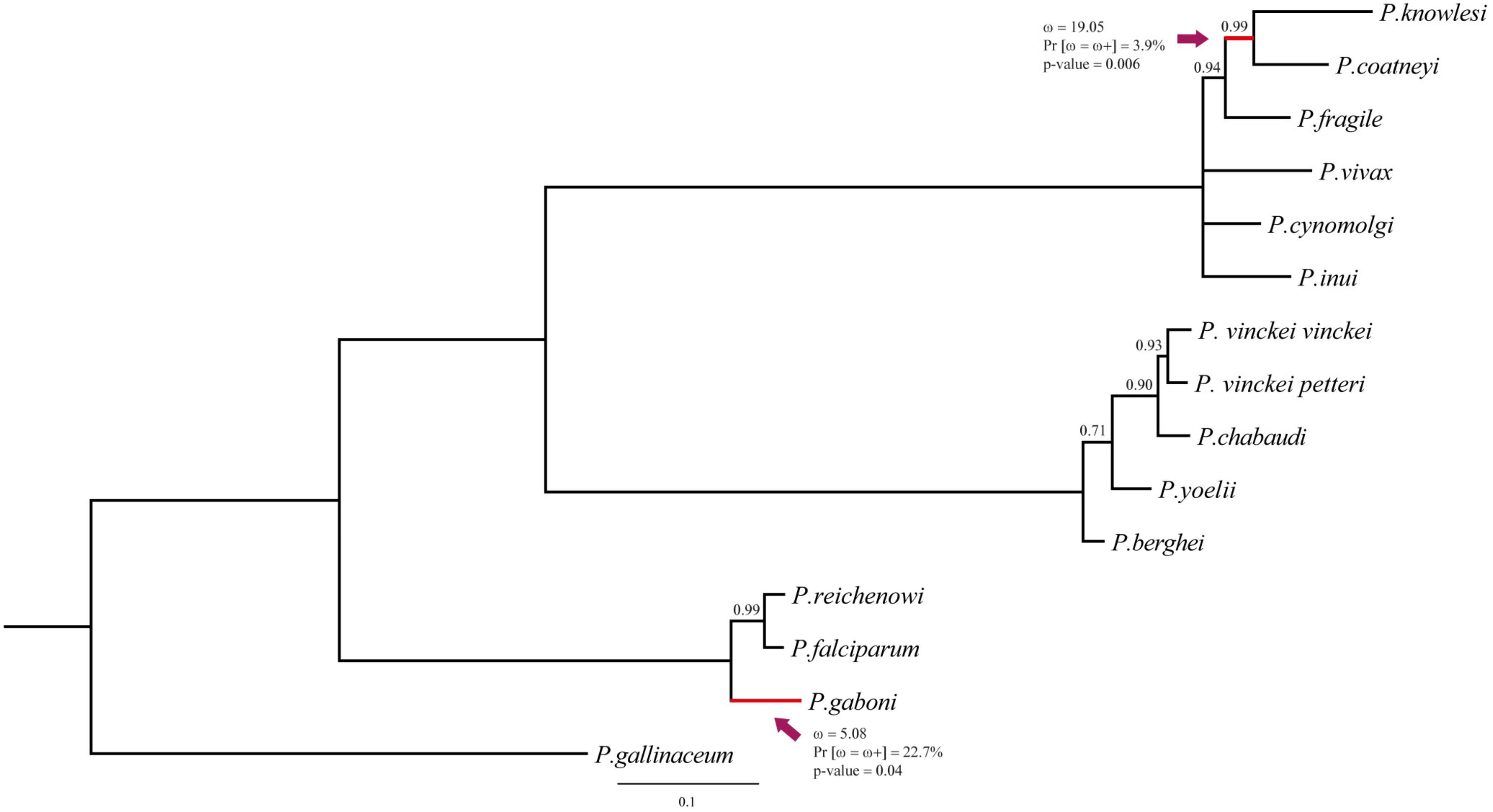
P.knowlesi
P.coatneyi
P.fragile
P.vivax
P.cynomolgi
P.inui
P. vinckei vinckei
P. vinckei petteri
P.chabaudi
P.yoelii
P.berghei
P.reichenowi
P.falciparum
P.gaboni
P.gallinaceum
0.99
0.94
0.93
0.90
0.71
0.99
ω = 19.05
Pr [ω = ω+] = 3.9%
p-value = 0.006
ω = 5.08
Pr [ω = ω+] = 22.7%
p-value = 0.04
0.1

C

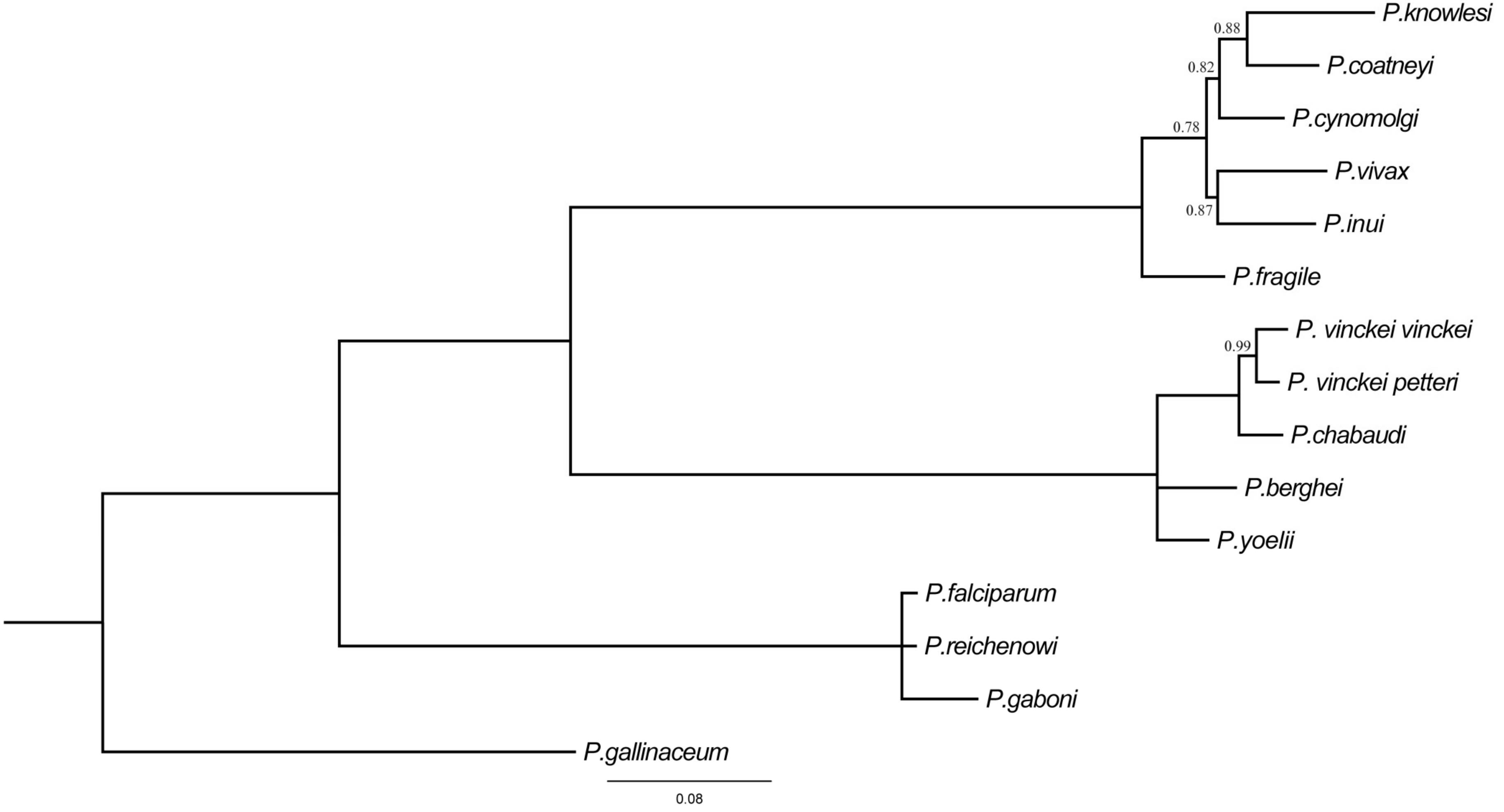
P.knowlesi
0.88
P.coatneyi
0.82
P.cynomolgi
0.78
P.vivax
0.87
P.inui
P.fragile
P. vinckei vinckei
0.99
P. vinckei petteri
P.chabaudi
P.berghei
P.yoelii
P.falciparum
P.reichenowi
P.gaboni
P.gallinaceum
0.08

D

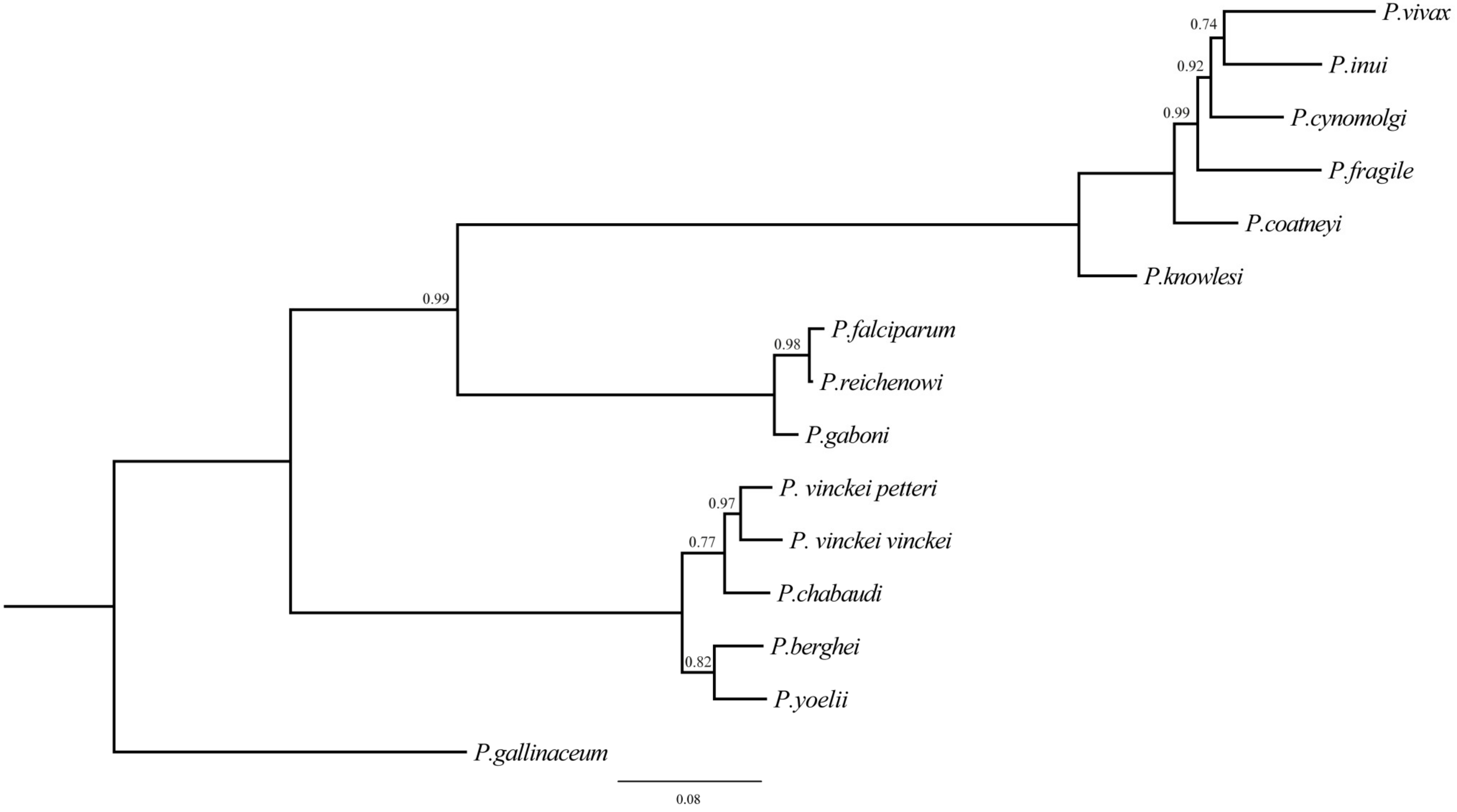
P.vivax
0.74
P.inui
0.92
P.cynomolgi
0.99
P.fragile
P.coatneyi
P.knowlesi
0.99
P.falciparum
0.98
P.reichenowi
P.gaboni
P. vinckei petteri
0.97
P. vinckei vinckei
0.77
P.chabaudi
P.berghei
0.82
P.yoelii
P.gallinaceum
0.08

E

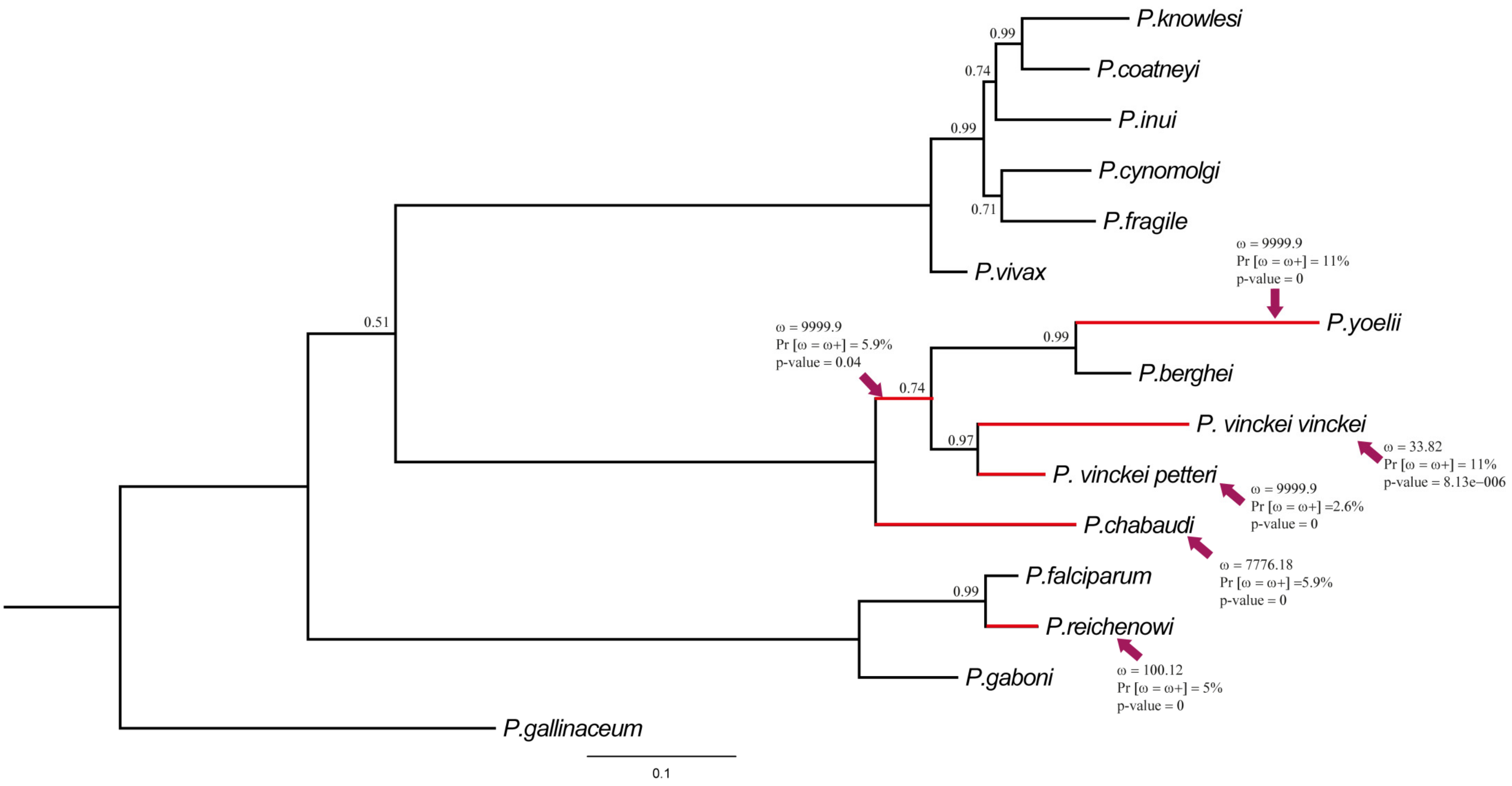
P.knowlesi
0.99
P.coatneyi
0.74
P.inui
0.99
P.cynomolgi
0.71
P.fragile
P.vivax
ω = 9999.9
Pr [ω = ω+] = 11%
p-value = 0
P.yoelii
0.51
ω = 9999.9
Pr [ω = ω+] = 5.9%
p-value = 0.04
0.99
P.berghei
0.74
P. vinckei vinckei
0.97
ω = 33.82
Pr [ω = ω+] = 11%
p-value = 8.13e–006
P. vinckei petteri
ω = 9999.9
Pr [ω = ω+] =2.6%
p-value = 0
P.chabaudi
ω = 7776.18
Pr [ω = ω+] =5.9%
p-value = 0
P.falciparum
0.99
P.reichenowi
ω = 100.12
Pr [ω = ω+] = 5%
p-value = 0
P.gaboni
P.gallinaceum
0.1

F

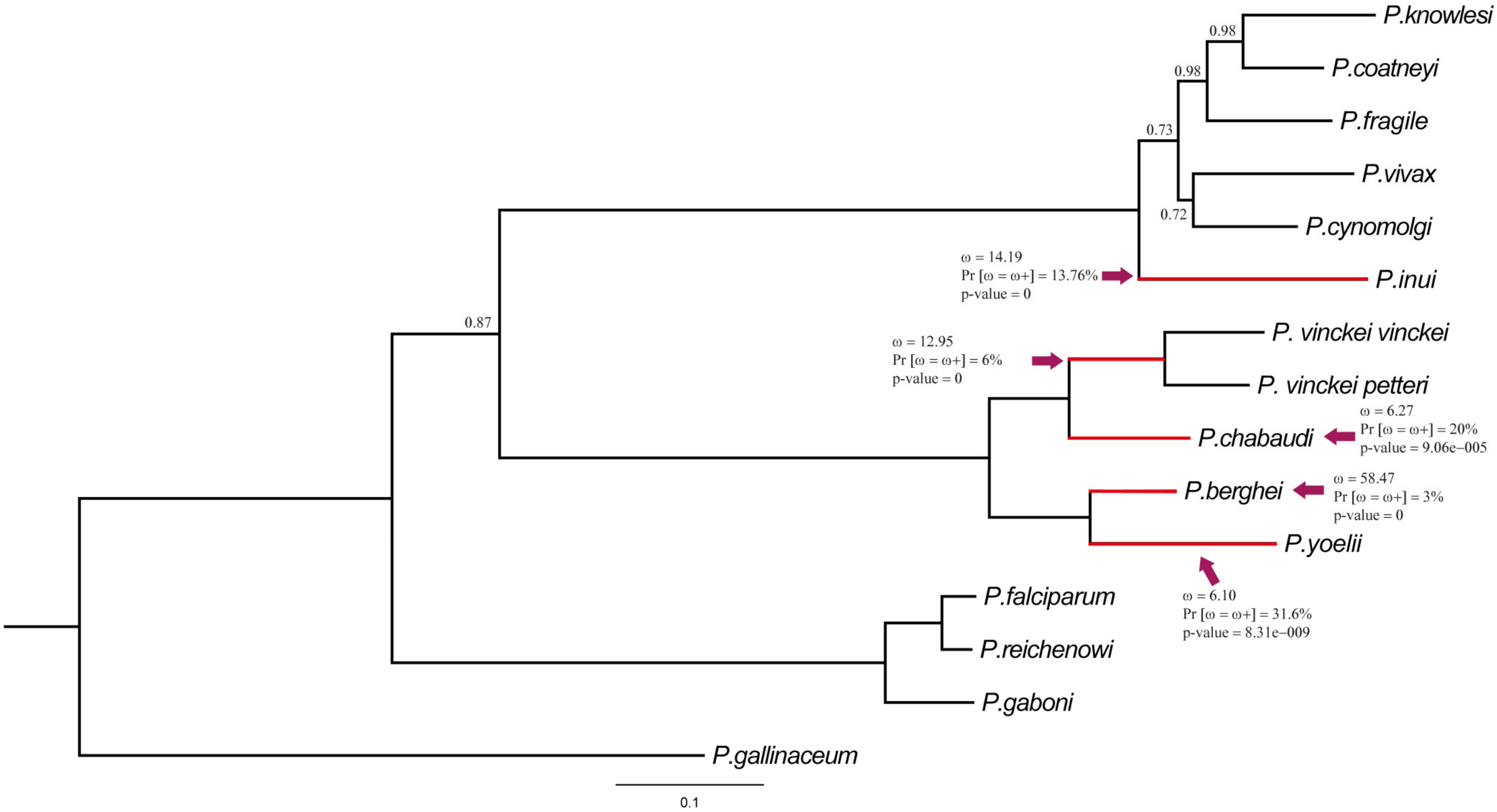
P.knowlesi
P.coatneyi
P.fragile
P.vivax
P.cynomolgi
P.inui
P. vinckei vinckei
P. vinckei petteri
P.chabaudi
P.berghei
P.yoelii
P.falciparum
P.reichenowi
P.gaboni
P.gallinaceum
0.98
0.98
0.73
0.72
0.87
ω = 14.19
Pr [ω = ω+] = 13.76%
p-value = 0
ω = 12.95
Pr [ω = ω+] = 6%
p-value = 0
ω = 6.27
Pr [ω = ω+] = 20%
p-value = 9.06e−005
ω = 58.47
Pr [ω = ω+] = 3%
p-value = 0
ω = 6.10
Pr [ω = ω+] = 31.6%
p-value = 8.31e−009
0.1

G

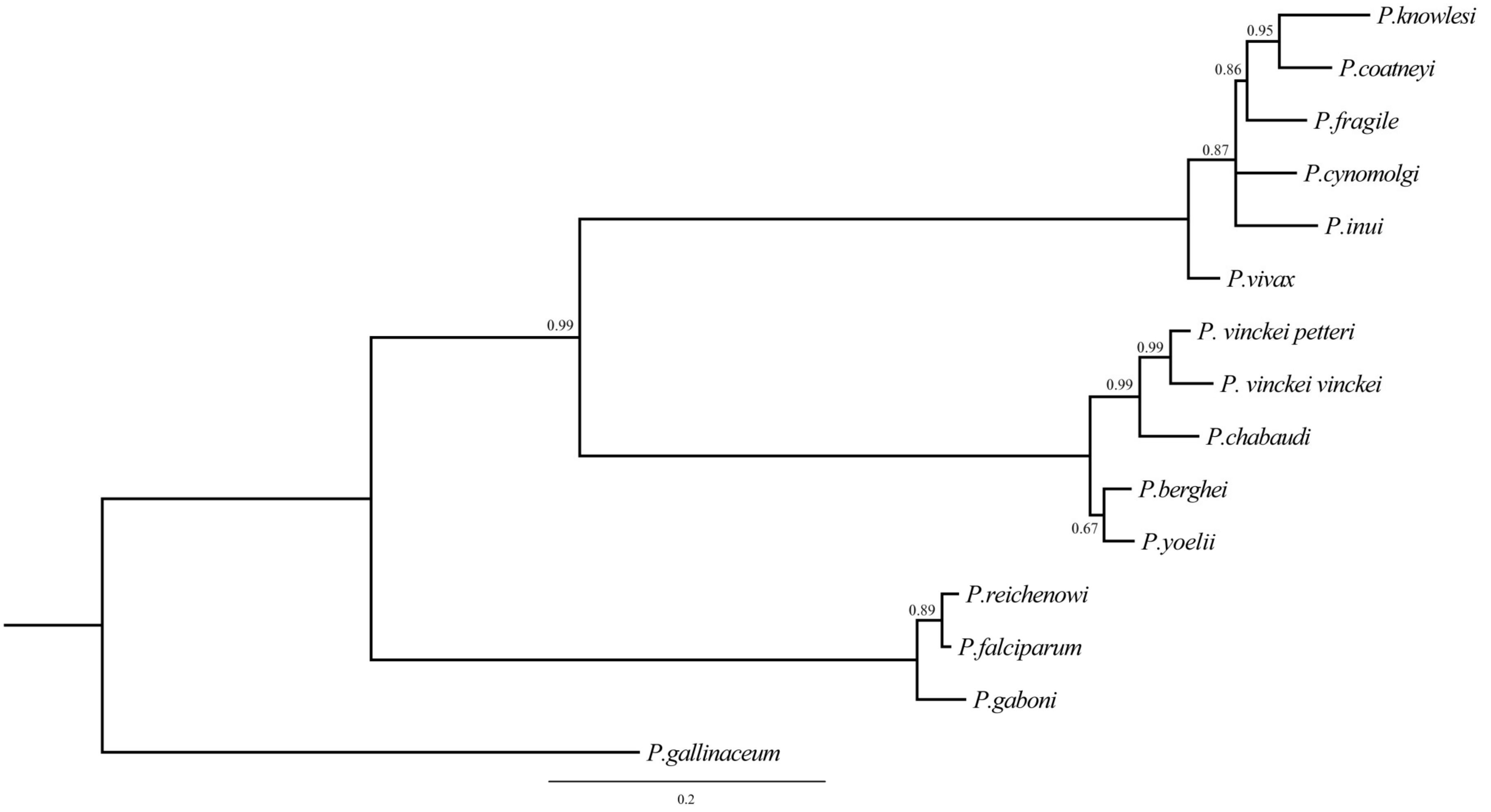
P.knowlesi
0.95
P.coatneyi
0.86
P.fragile
0.87
P.cynomolgi
P.inui
P.vivax
0.99
P. vinckei petteri
0.99
P. vinckei vinckei
0.99
P.chabaudi
P.berghei
0.67
P.yoelii
P.reichenowi
0.89
P.falciparum
P.gaboni
P.gallinaceum
0.2

H

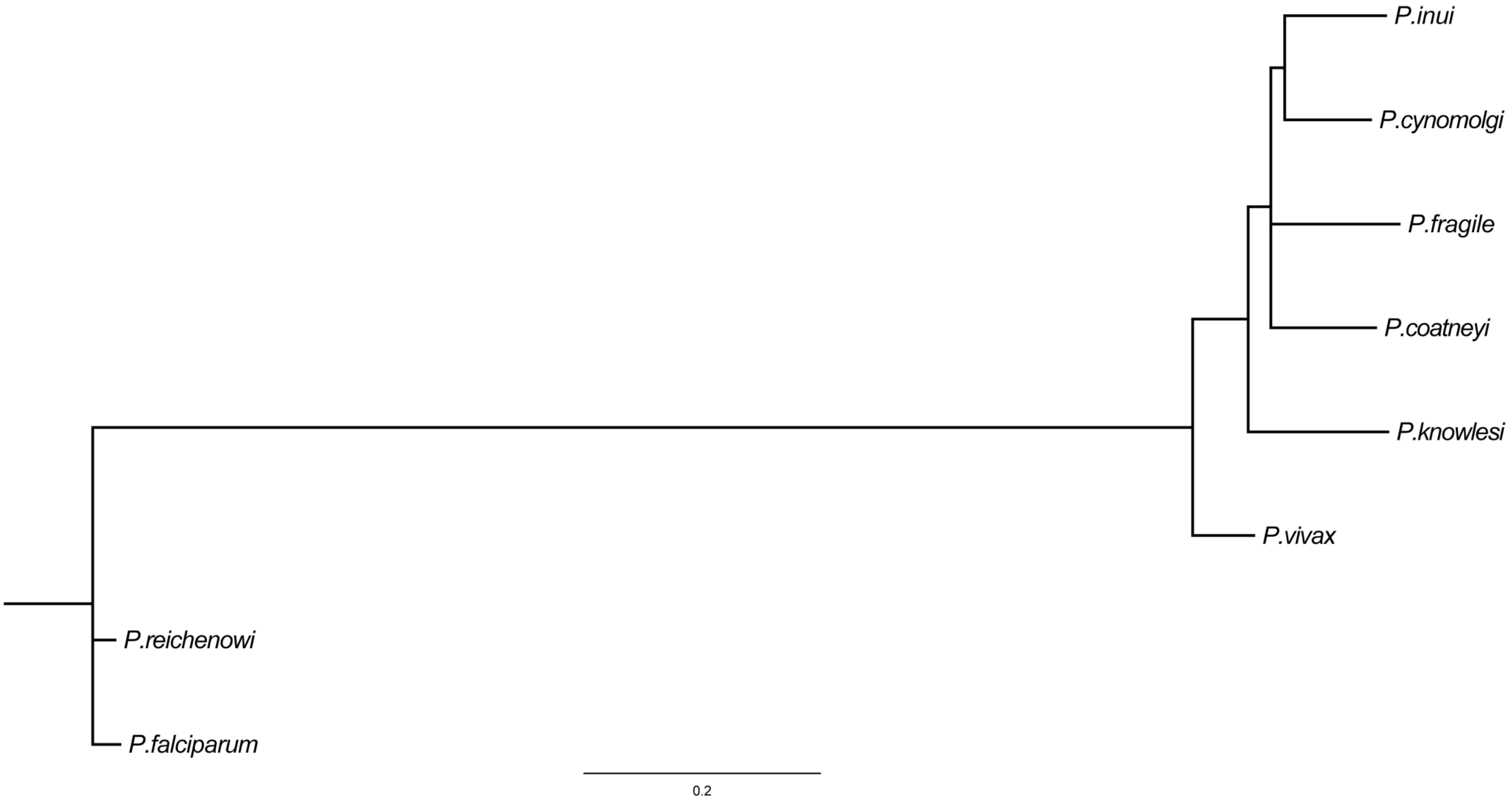

P.inui
P.cynomolgi
P.fragile
P.coatneyi
P.knowlesi
P.vivax
P.reichenowi
P.falciparum
0.2

I

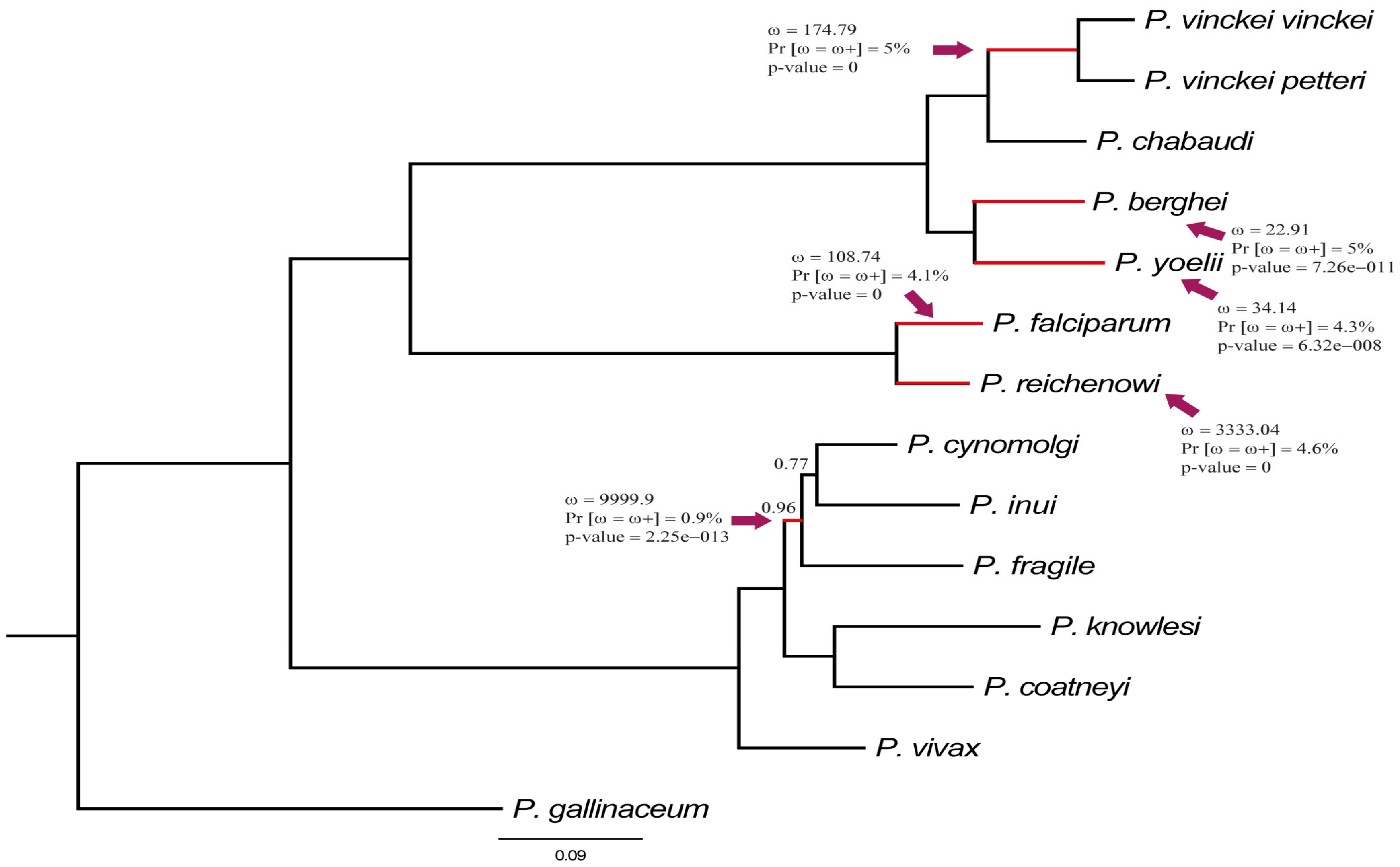
ω = 174.79
Pr [ω = ω+] = 5%
p-value = 0
P. vinckei vinckei
P. vinckei petteri
P. chabaudi
P. berghei
ω = 22.91
Pr [ω = ω+] = 5%
p-value = 7.26e–011
P. yoelii
ω = 108.74
Pr [ω = ω+] = 4.1%
p-value = 0
ω = 34.14
Pr [ω = ω+] = 4.3%
p-value = 6.32e–008
P. falciparum
P. reichenowi
ω = 3333.04
Pr [ω = ω+] = 4.6%
p-value = 0
P. cynomolgi
0.77
P. inui
ω = 9999.9
Pr [ω = ω+] = 0.9%
p-value = 2.25e–013
0.96
P. fragile
P. knowlesi
P. coatneyi
P. vivax
P. gallinaceum
0.09

J

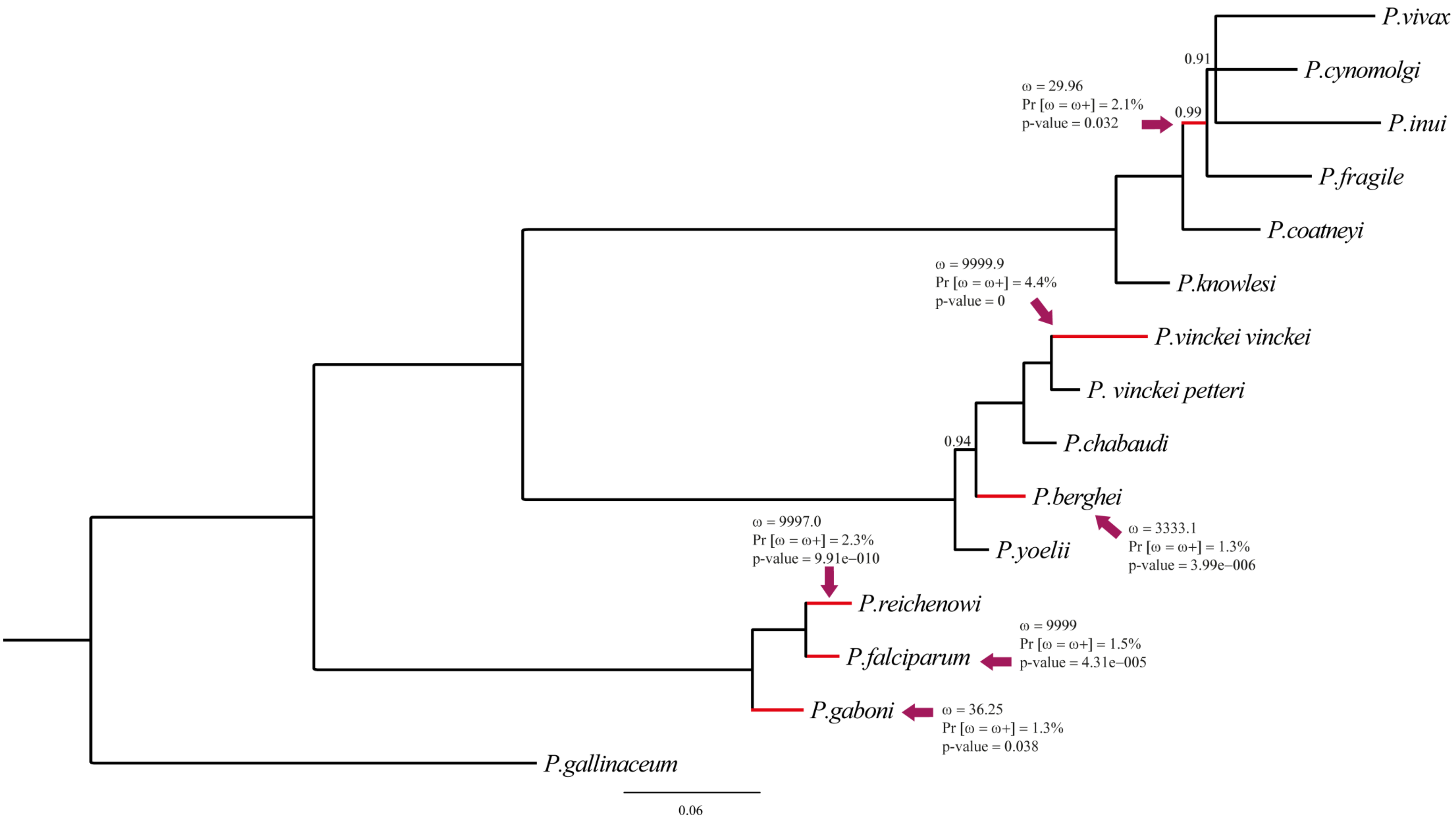
P.vivax
0.91
P.cynomolgi
ω = 29.96
Pr [ω = ω+] = 2.1%
p-value = 0.032
0.99
P.inui
P.fragile
P.coatneyi
P.knowlesi
ω = 9999.9
Pr [ω = ω+] = 4.4%
p-value = 0
P.vinckei vinckei
P. vinckei petteri
0.94
P.chabaudi
P.berghei
ω = 3333.1
Pr [ω = ω+] = 1.3%
p-value = 3.99e–006
ω = 9997.0
Pr [ω = ω+] = 2.3%
p-value = 9.91e–010
P.yoelii
P.reichenowi
ω = 9999
Pr [ω = ω+] = 1.5%
p-value = 4.31e–005
P.falciparum
P.gaboni
ω = 36.25
Pr [ω = ω+] = 1.3%
p-value = 0.038
P.gallinaceum
0.06

K

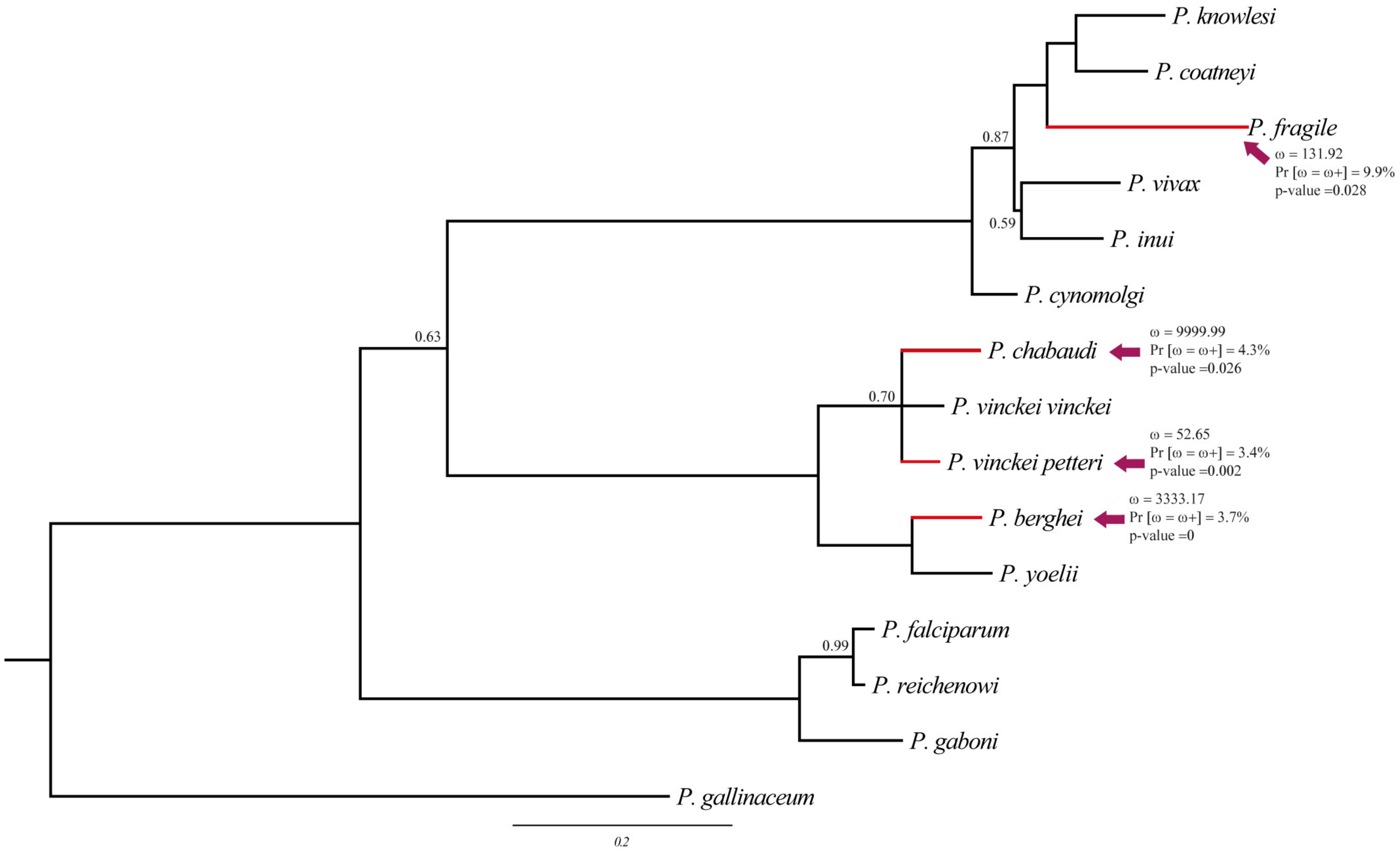
P. knowlesi
P. coatneyi
P. fragile
ω = 131.92
Pr [ω = ω+] = 9.9%
p-value =0.028
0.87
P. vivax
0.59
P. inui
P. cynomolgi
0.63
P. chabaudi
ω = 9999.99
Pr [ω = ω+] = 4.3%
p-value =0.026
0.70
P. vinckei vinckei
P. vinckei petteri
ω = 52.65
Pr [ω = ω+] = 3.4%
p-value =0.002
P. berghei
ω = 3333.17
Pr [ω = ω+] = 3.7%
p-value =0
P. yoelii
0.99
P. falciparum
P. reichenowi
P. gaboni
P. gallinaceum
0.2

L

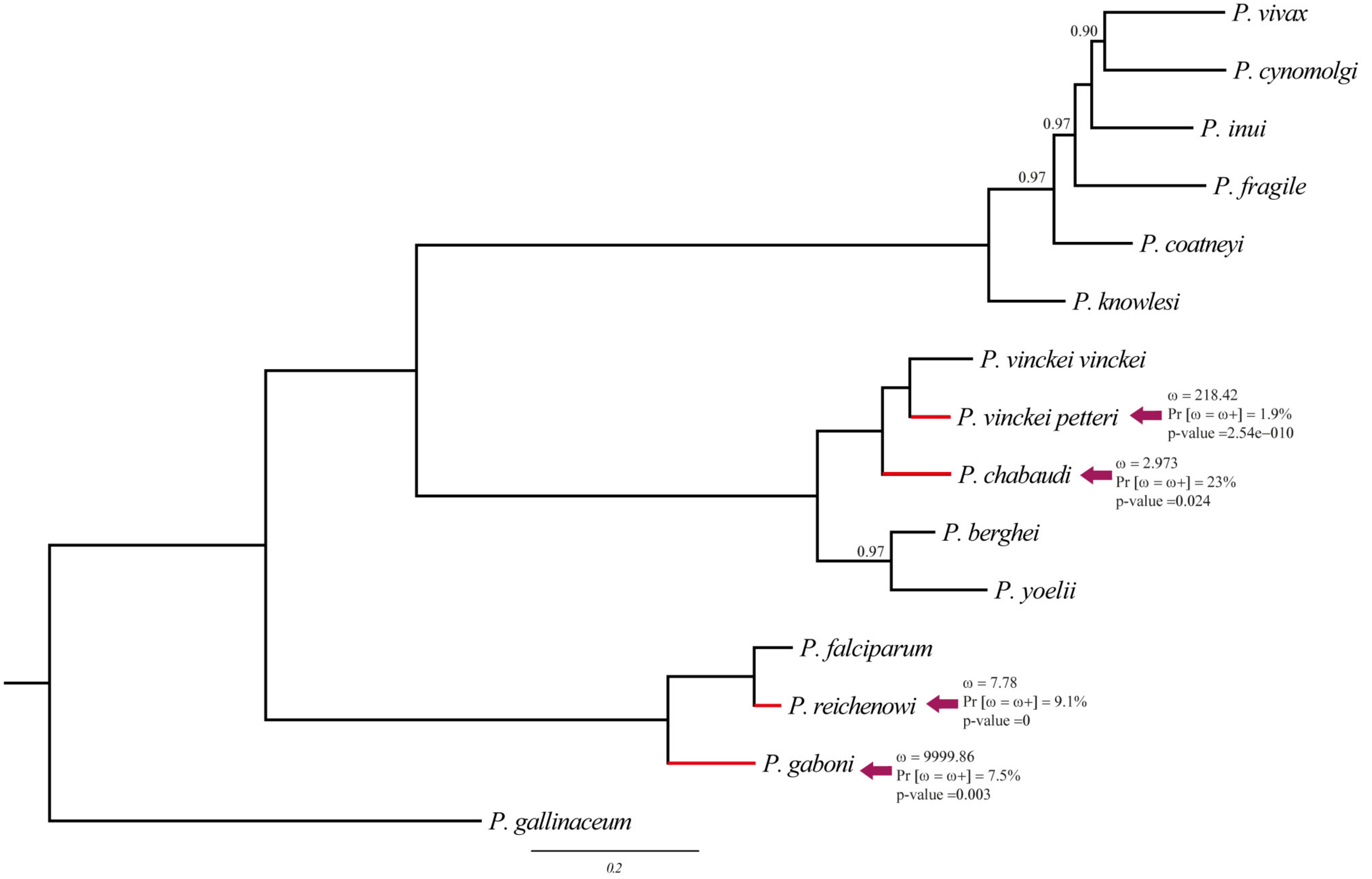
P. vivax
P. cynomolgi
P. inui
P. fragile
P. coatneyi
P. knowlesi
P. vinckei vinckei
P. vinckei petteri
P. chabaudi
P. berghei
P. yoelii
P. falciparum
P. reichenowi
P. gaboni
P. gallinaceum
0.90
0.97
0.97
0.97
ω = 218.42
Pr [ω = ω+] = 1.9%
p-value =2.54e−010
ω = 2.973
Pr [ω = ω+] = 23%
p-value =0.024
ω = 7.78
Pr [ω = ω+] = 9.1%
p-value =0
ω = 9999.86
Pr [ω = ω+] = 7.5%
p-value =0.003
0.2

M

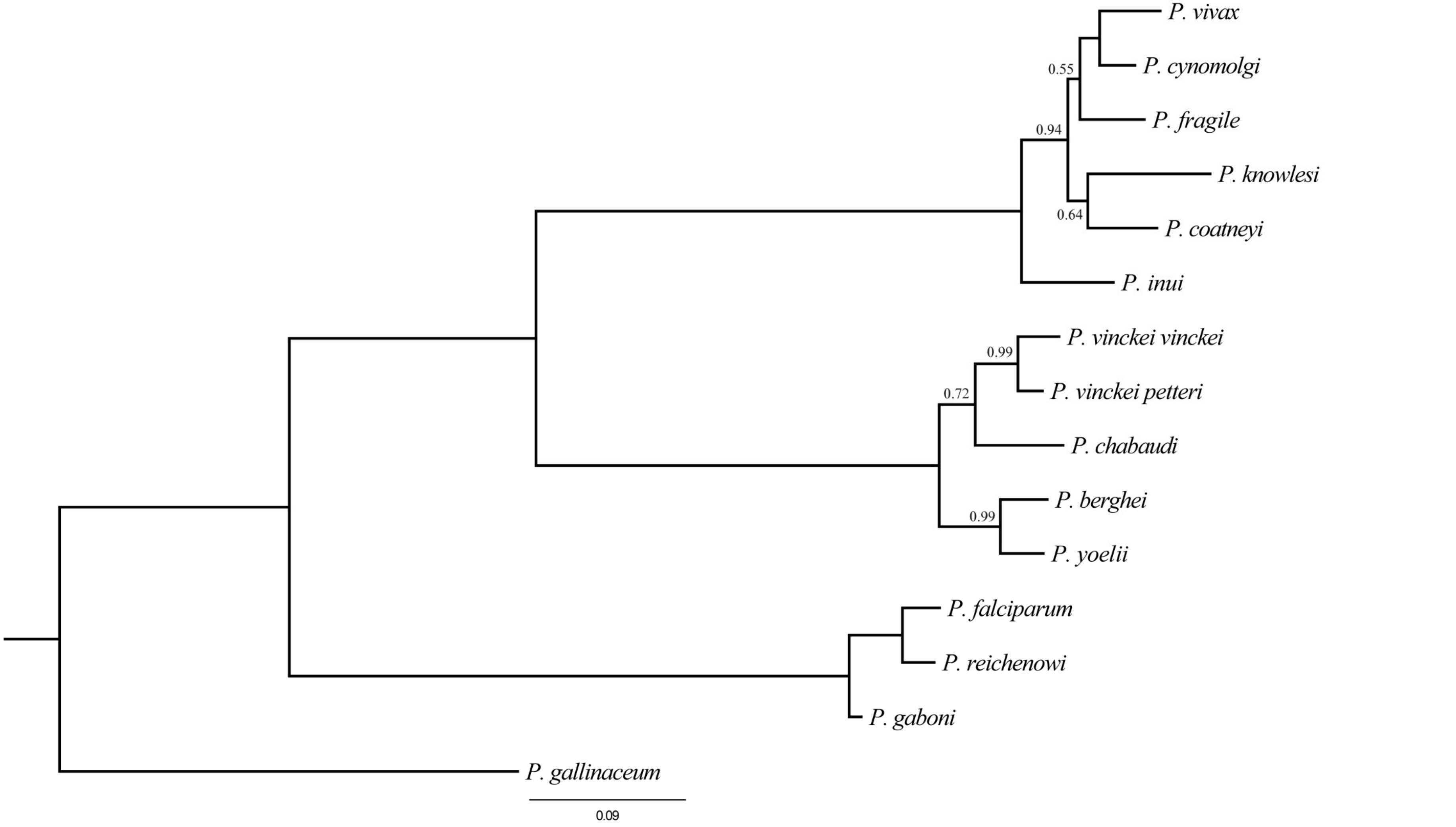
P. vivax
P. cynomolgi
0.55
P. fragile
0.94
P. knowlesi
0.64
P. coatneyi
P. inui
P. vinckei vinckei
0.99
P. vinckei petteri
0.72
P. chabaudi
P. berghei
0.99
P. yoelii
P. falciparum
P. reichenowi
P. gaboni
P. gallinaceum
0.09

N

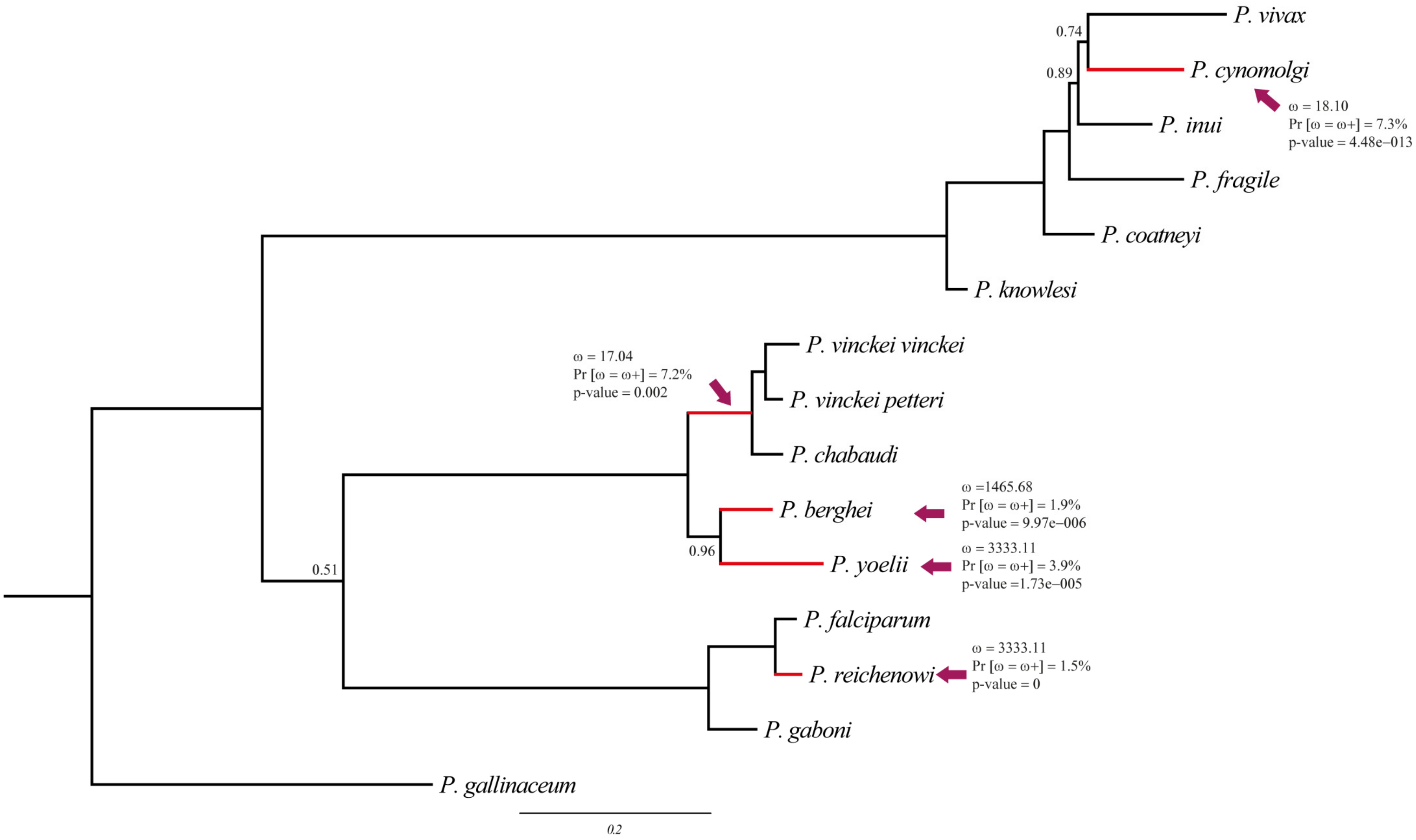
P. vivax
0.74
P. cynomolgi
0.89
ω = 18.10
Pr [ω = ω+] = 7.3%
p-value = 4.48e–013
P. inui
P. fragile
P. coatneyi
P. knowlesi
ω = 17.04
Pr [ω = ω+] = 7.2%
p-value = 0.002
P. vinckei vinckei
P. vinckei petteri
P. chabaudi
P. berghei
ω =1465.68
Pr [ω = ω+] = 1.9%
p-value = 9.97e–006
0.96
P. yoelii
ω = 3333.11
Pr [ω = ω+] = 3.9%
p-value =1.73e–005
0.51
P. falciparum
P. reichenowi
ω = 3333.11
Pr [ω = ω+] = 1.5%
p-value = 0
P. gaboni
P. gallinaceum
0.2

Supplementary Material 3. Phylogenies analysed for episodic selection. The Branch-site REL method was used for analysing orthologous loci. Red shading indicates branches under episodic selection. The selection stretch, the percentage of sites under selection and the p-values are indicated on each selected branch. Branches have been classified as undergoing episodic diversifying selection by the p-value corrected for multiple testing using the Holm-Bonferroni method at $p < 0.05$. Just the posterior Bayesian probability values lower than 1 are shown on branches. A. *siap1* (PVX_000815), B. *p52* (PVX_001020), C. *p36* (PVX_001025), D. *spatr* (PVX_002900), E. *trsp* (PVX_081560), F. *trap* (PVX_082735), G. *spect1* (PVX_083025), H. *siap2* (PVX_088860), I. *maebl* (PVX_092975), J. *plp1* (PVX_000810), K. *mcp1* (PVX_111355), L. *tlp* (PVX_113965), M. *celtos* (PVX_123510), L. *mb2* (PVX_080420).
