# Supplementary material for: Identifying Potential Plasmodium vivax Sporozoite Stage Vaccine Candidates: An Analysis of Genetic Diversity and Natural Selection
Source: Front Genet. 2018 Jan 25;9:10. doi: 10.3389/fgene.2018.00010 (PMC5788960; doi:10.3389/fgene.2018.00010)
Supplement: Supplementary file 4 [file Presentation4.PDF]

Supplementary Material 4. Linear B-epitopes and surface accessibility scores.

Identifying potential *P. vivax* sporozoite stage vaccine candidates: an analysis of genetic diversity and natural selection

Diego Garzón-Ospina, Sindy Paola Buitrago, Andrea Estefania Ramos, Manuel A. Patarroyo*

*** Correspondence:** mapatarr.fidic@gmail.com

A

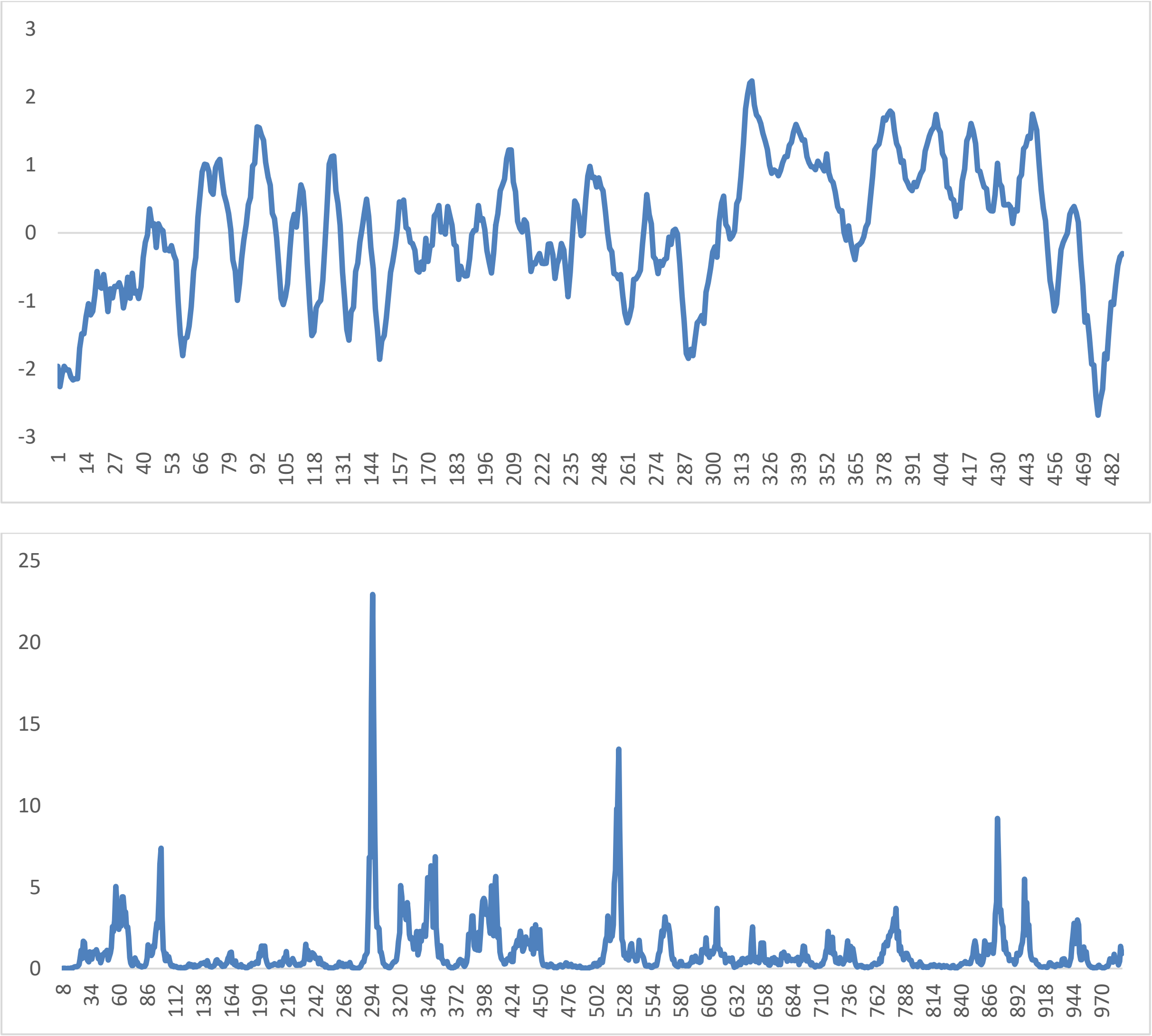
3
2
1
0
-1
-2
-3
1 14 27 40 53 66 79 92 105 118 131 144 157 170 183 196 209 222 235 248 261 274 287 300 313 326 339 352 365 378 391 404 417 430 443 456 469 482
25
20
15
10
5
0
8 34 60 86 112 138 164 190 216 242 268 294 320 346 372 398 424 450 476 502 528 554 580 606 632 658 684 710 736 762 788 814 840 866 892 918 944 970

B

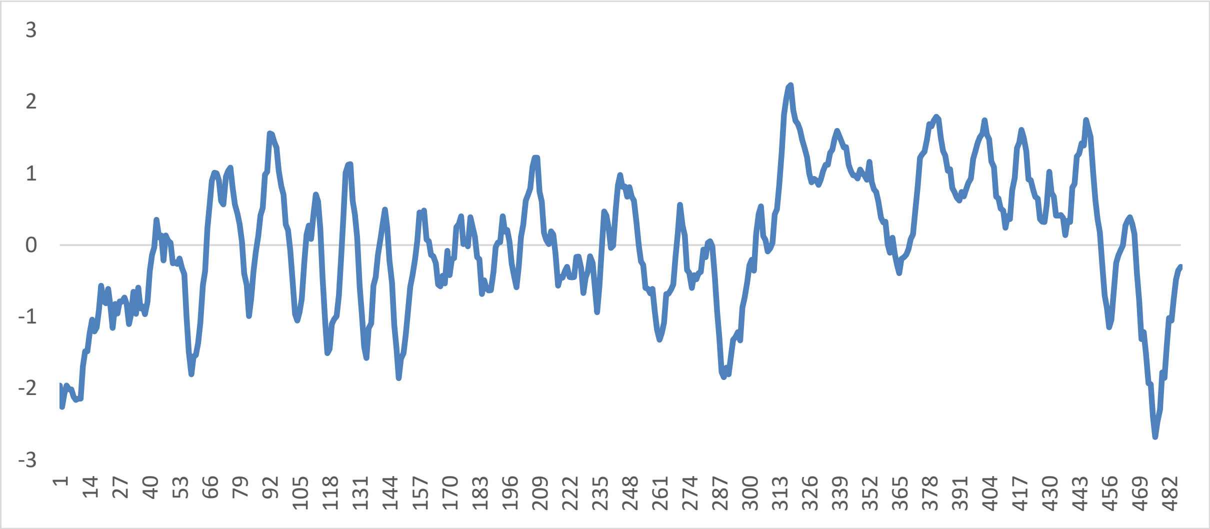

3
2
1
0
-1
-2
-3
1 14 27 40 53 66 79 92 105 118 131 144 157 170 183 196 209 222 235 248 261 274 287 300 313 326 339 352 365 378 391 404 417 430 443 456 469 482

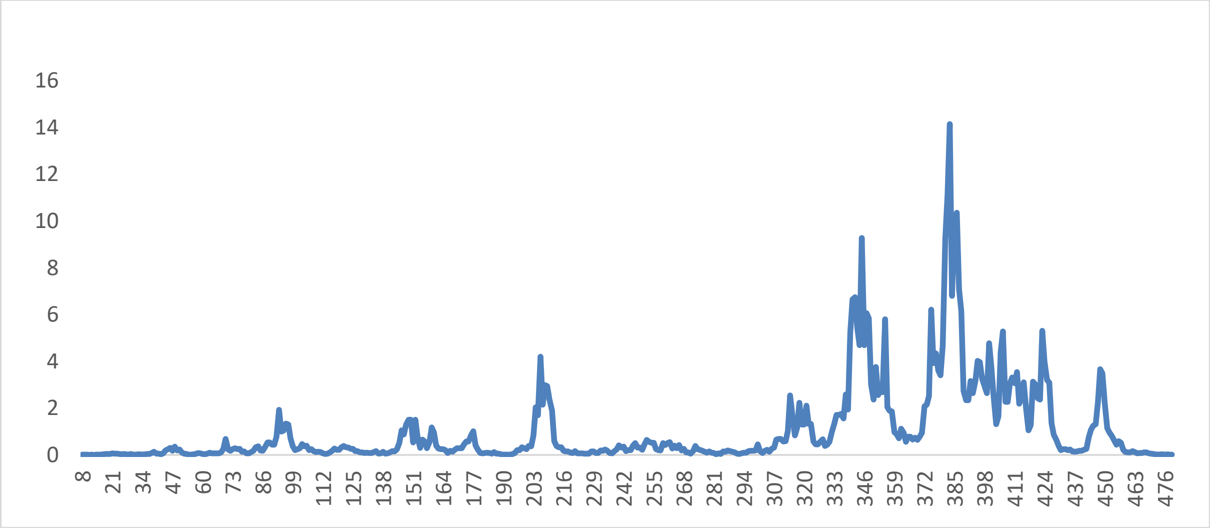

16
14
12
10
8
6
4
2
0
8 21 34 47 60 73 86 99 112 125 138 151 164 177 190 203 216 229 242 255 268 281 294 307 320 333 346 359 372 385 398 411 424 437 450 463 476

C

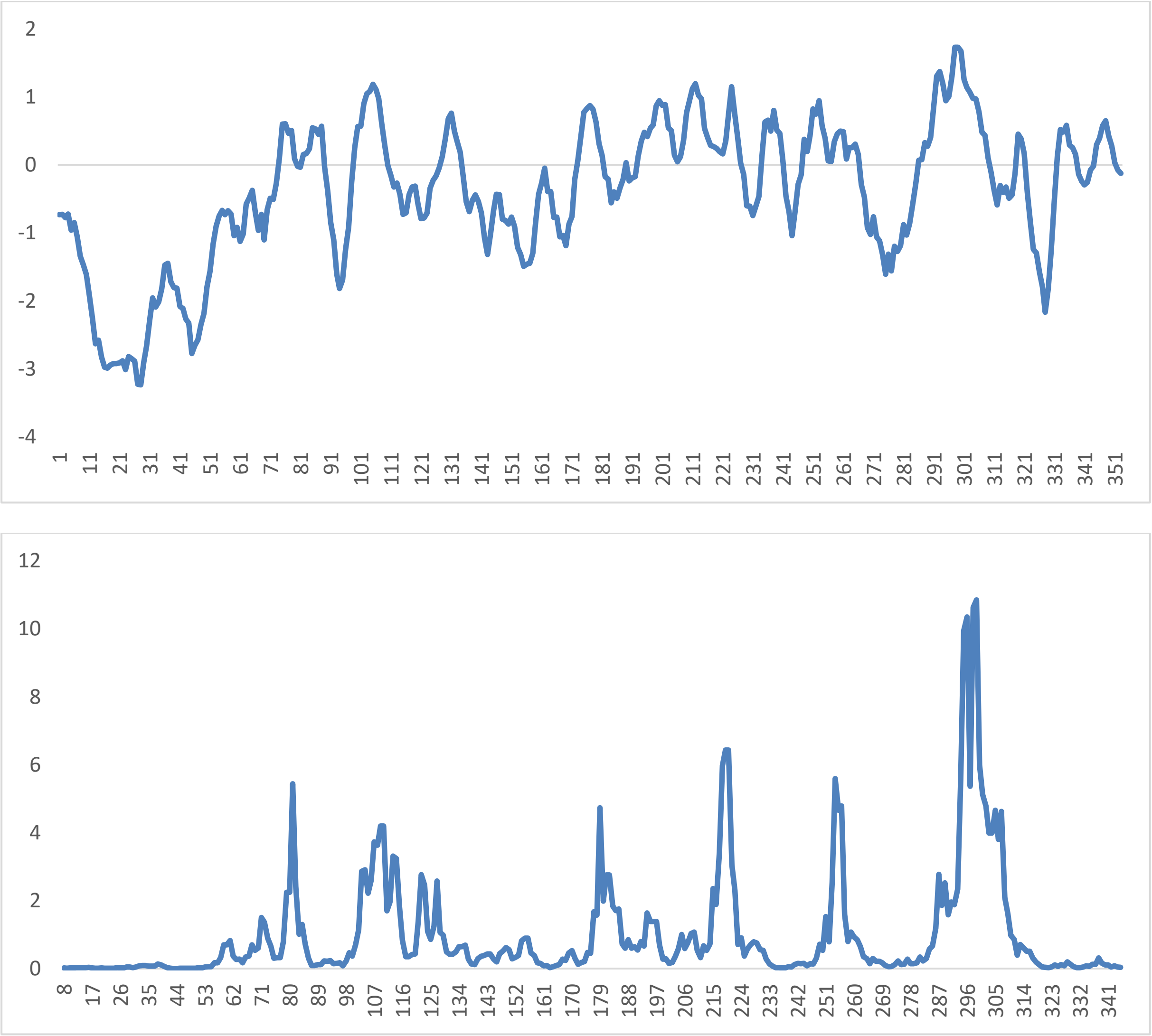
2
1
0
-1
-2
-3
-4
1 11 21 31 41 51 61 71 81 91 101 111 121 131 141 151 161 171 181 191 201 211 221 231 241 251 261 271 281 291 301 311 321 331 341 351
12
10
8
6
4
2
0
8 17 26 35 44 53 62 71 80 89 98 107 116 125 134 143 152 161 170 179 188 197 206 215 224 233 242 251 260 269 278 287 296 305 314 323 332 341

D

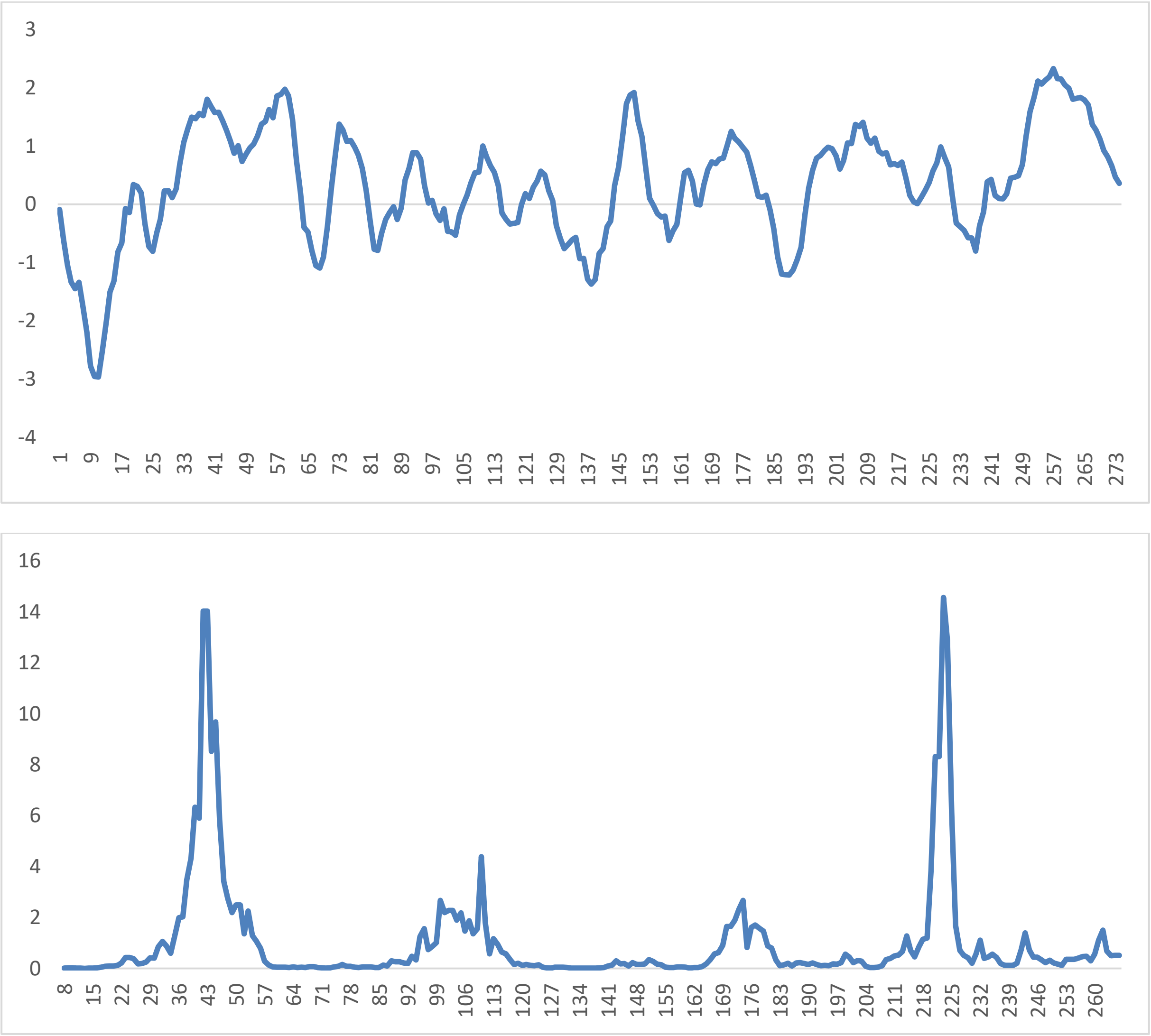
3
2
1
0
-1
-2
-3
-4
1 9 17 25 33 41 49 57 65 73 81 89 97 105 113 121 129 137 145 153 161 169 177 185 193 201 209 217 225 233 241 249 257 265 273
16
14
12
10
8
6
4
2
0
8 15 22 29 36 43 50 57 64 71 78 85 92 99 106 113 120 127 134 141 148 155 162 169 176 183 190 197 204 211 218 225 232 239 246 253 260

E

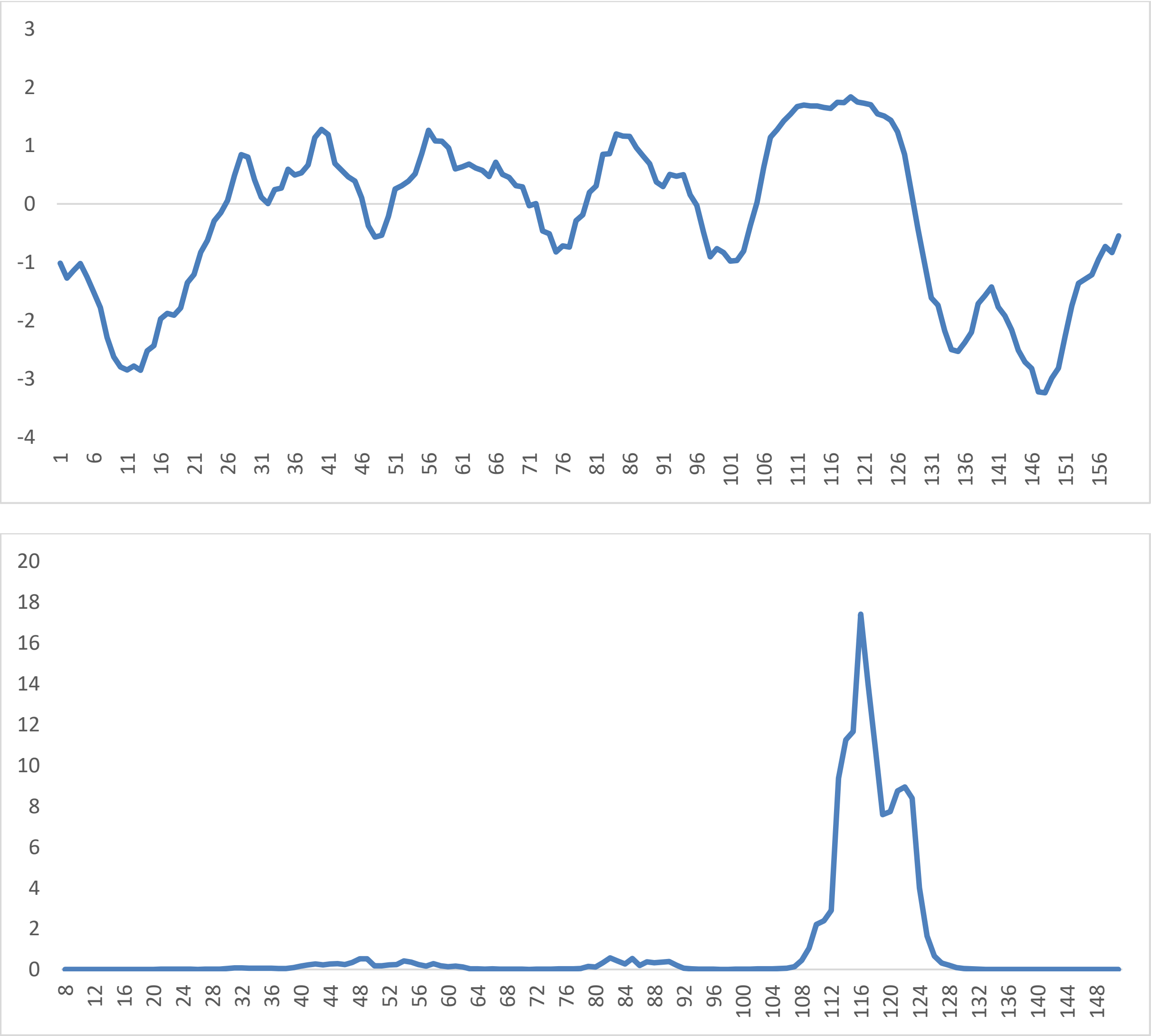
3
2
1
0
-1
-2
-3
-4
1 6 11 16 21 26 31 36 41 46 51 56 61 66 71 76 81 86 91 96 101 106 111 116 121 126 131 136 141 146 151 156
20
18
16
14
12
10
8
6
4
2
0
8 12 16 20 24 28 32 36 40 44 48 52 56 60 64 68 72 76 80 84 88 92 96 100 104 108 112 116 120 124 128 132 136 140 144 148

F

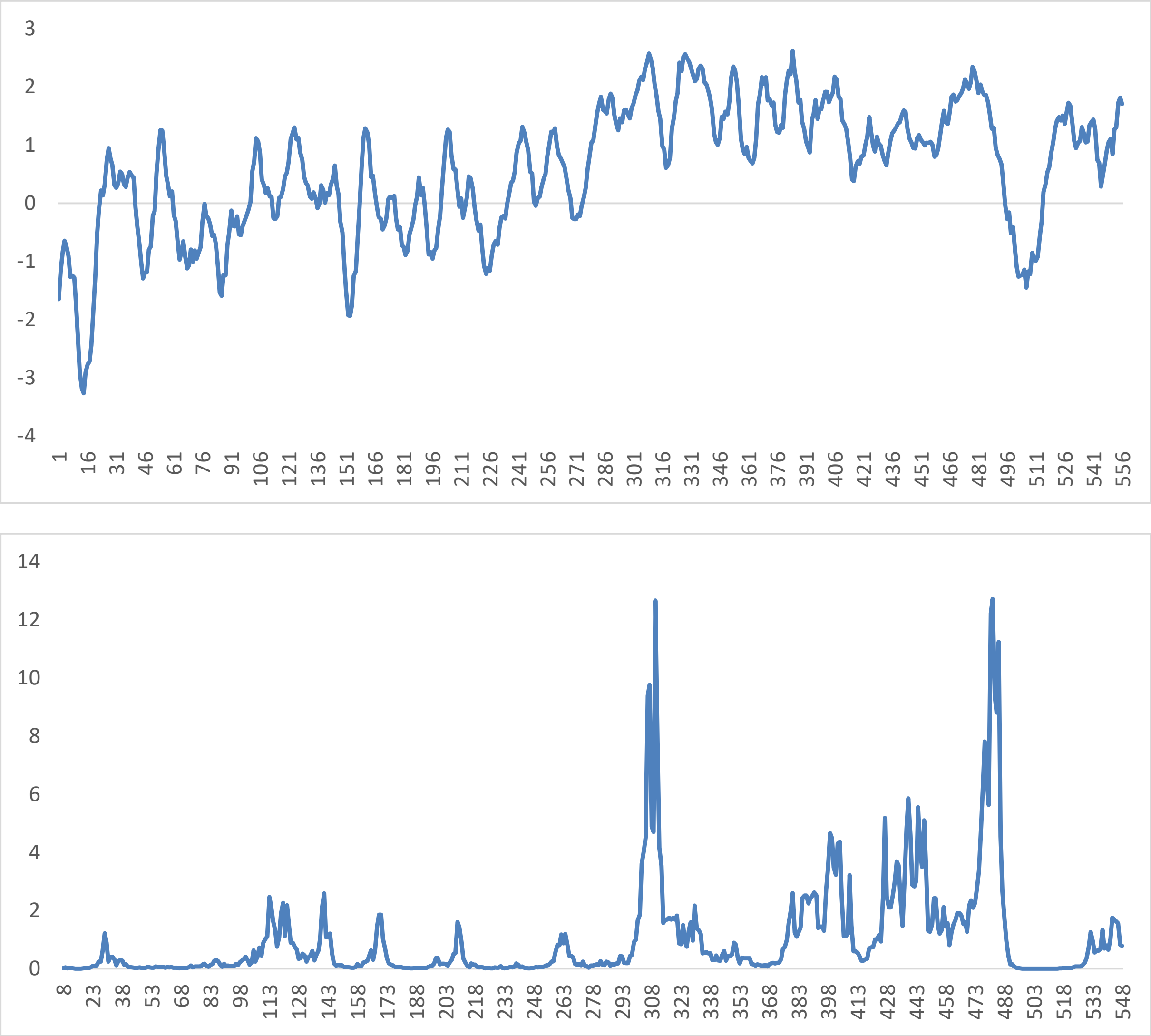
3
2
1
0
-1
-2
-3
-4
1 16 31 46 61 76 91 106 121 136 151 166 181 196 211 226 241 256 271 286 301 316 331 346 361 376 391 406 421 436 451 466 481 496 511 526 541 556
14
12
10
8
6
4
2
0
8 23 38 53 68 83 98 113 128 143 158 173 188 203 218 233 248 263 278 293 308 323 338 353 368 383 398 413 428 443 458 473 488 503 518 533 548

G

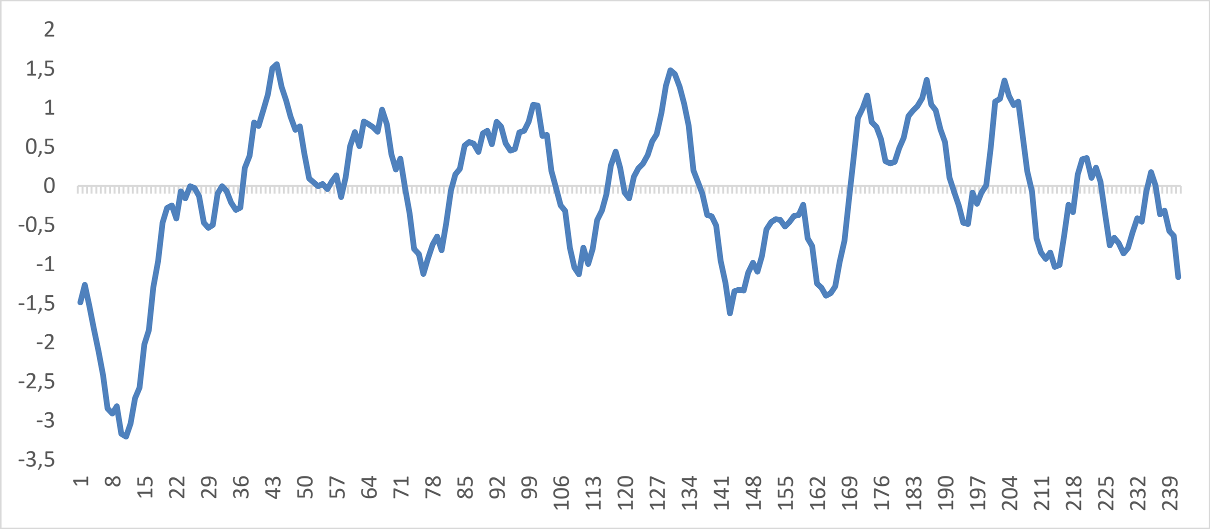
2
1,5
1
0,5
0
-0,5
-1
-1,5
-2
-2,5
-3
-3,5
1 8 15 22 29 36 43 50 57 64 71 78 85 92 99 106 113 120 127 134 141 148 155 162 169 176 183 190 197 204 211 218 225 232 239

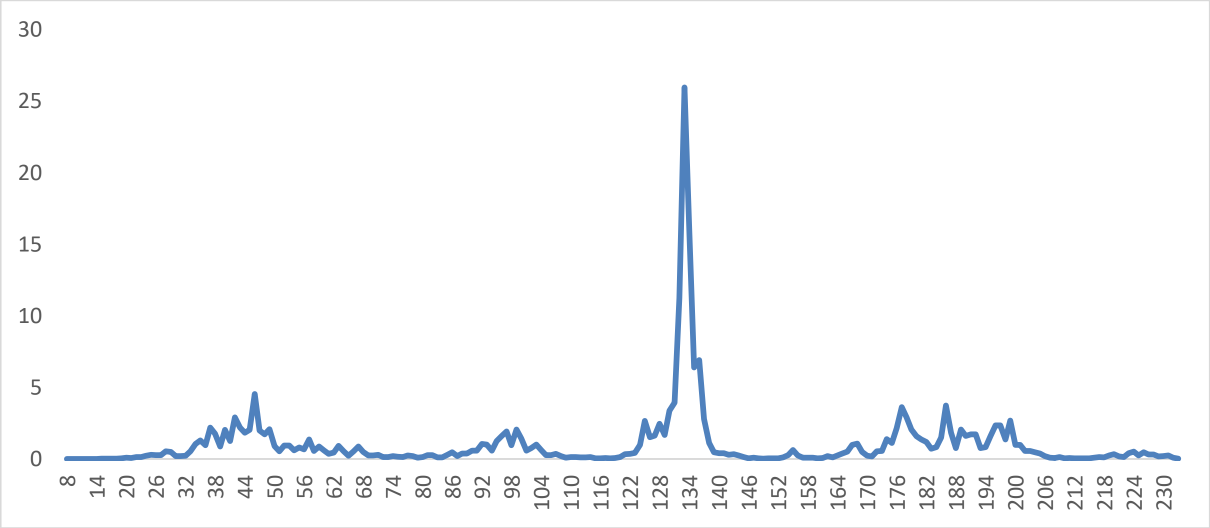
30
25
20
15
10
5
0
8 14 20 26 32 38 44 50 56 62 68 74 80 86 92 98 104 110 116 122 128 134 140 146 152 158 164 170 176 182 188 194 200 206 212 218 224 230

H

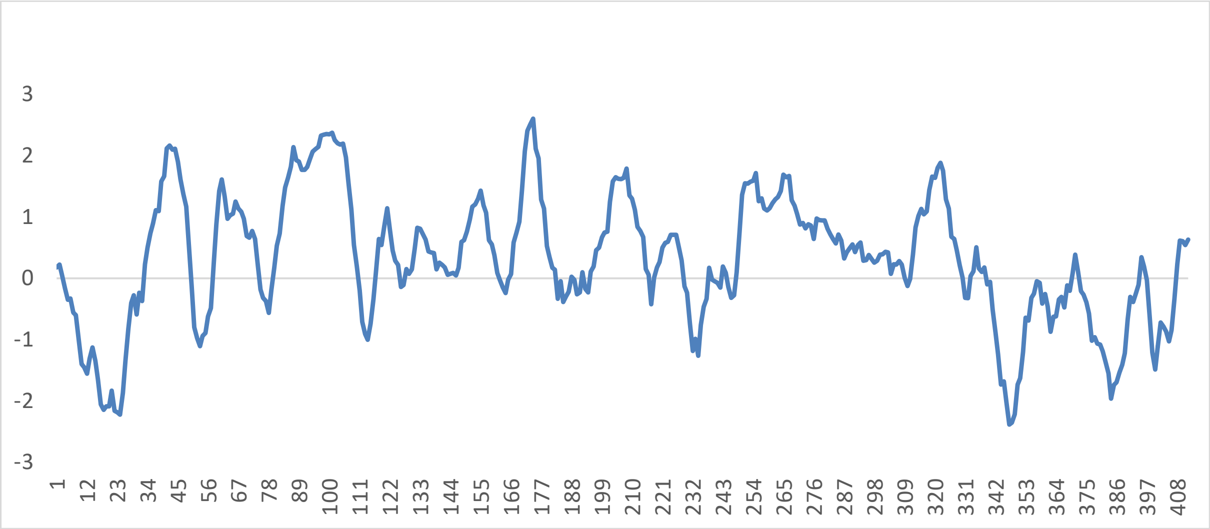
3
2
1
0
-1
-2
-3
1
12
23
34
45
56
67
78
89
100
111
122
133
144
155
166
177
188
199
210
221
232
243
254
265
276
287
298
309
320
331
342
353
364
375
386
397
408

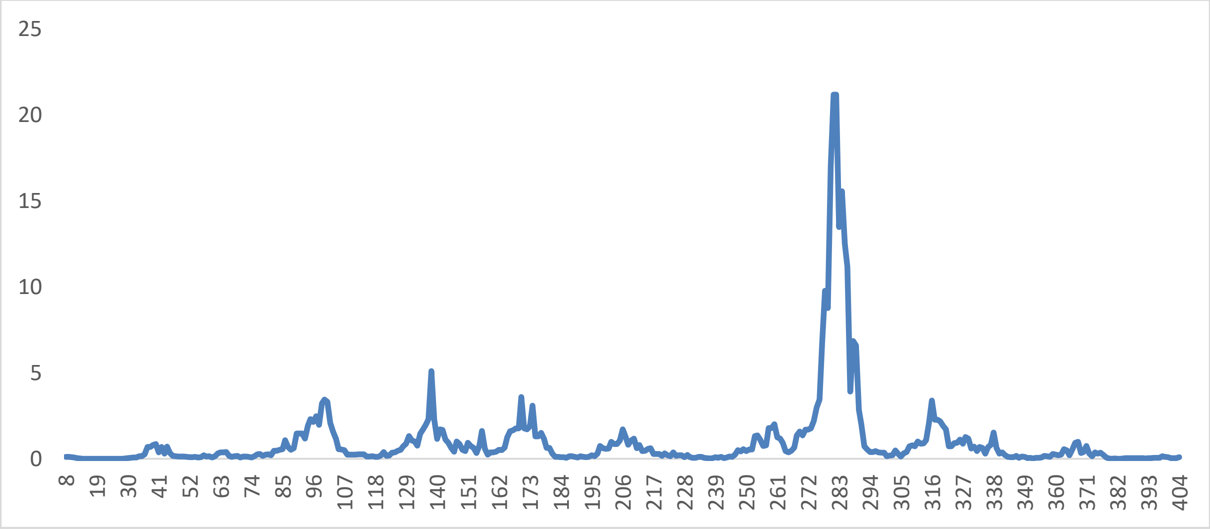
25
20
15
10
5
0
8
19
30
41
52
63
74
85
96
107
118
129
140
151
162
173
184
195
206
217
228
239
250
261
272
283
294
305
316
327
338
349
360
371
382
393
404

I

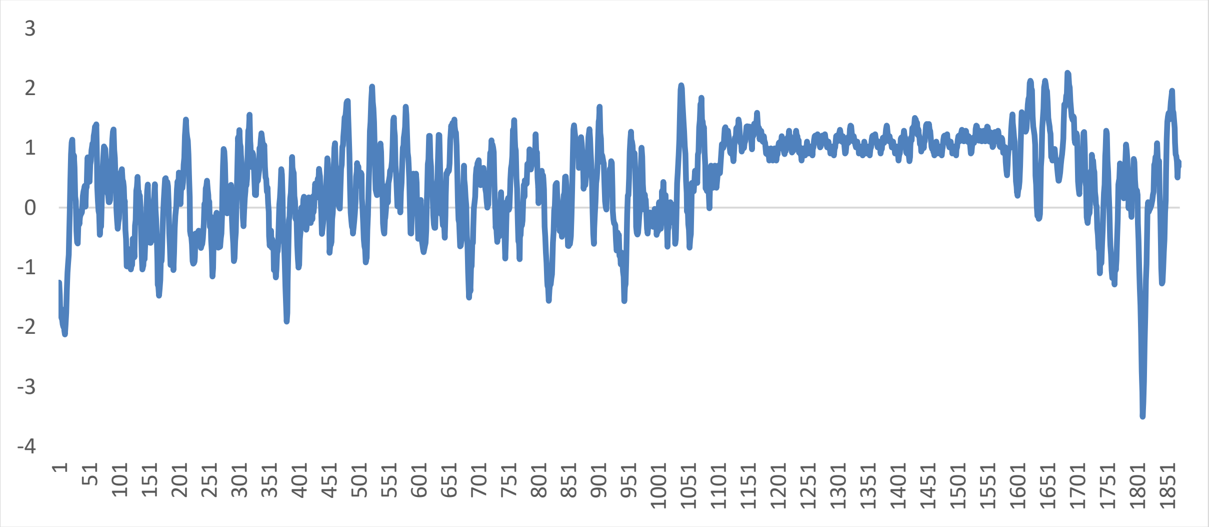
3
2
1
0
-1
-2
-3
-4
1 51 101 151 201 251 301 351 401 451 501 551 601 651 701 751 801 851 901 951 1001 1051 1101 1151 1201 1251 1301 1351 1401 1451 1501 1551 1601 1651 1701 1751 1801 1851

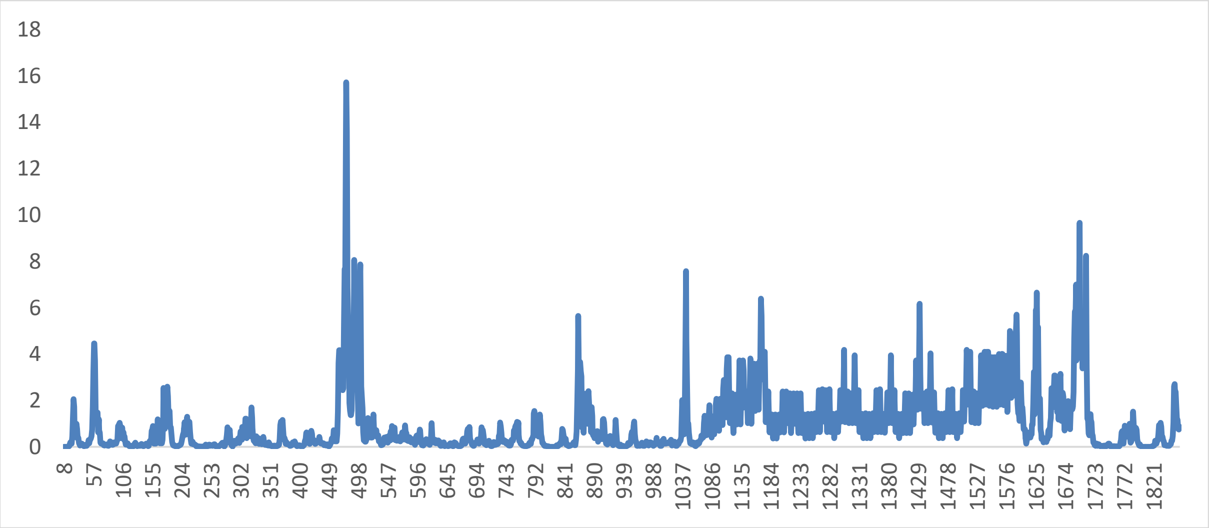
18
16
14
12
10
8
6
4
2
0
8 57 106 155 204 253 302 351 400 449 498 547 596 645 694 743 792 841 890 939 988 1037 1086 1135 1184 1233 1282 1331 1380 1429 1478 1527 1576 1625 1674 1723 1772 1821

J

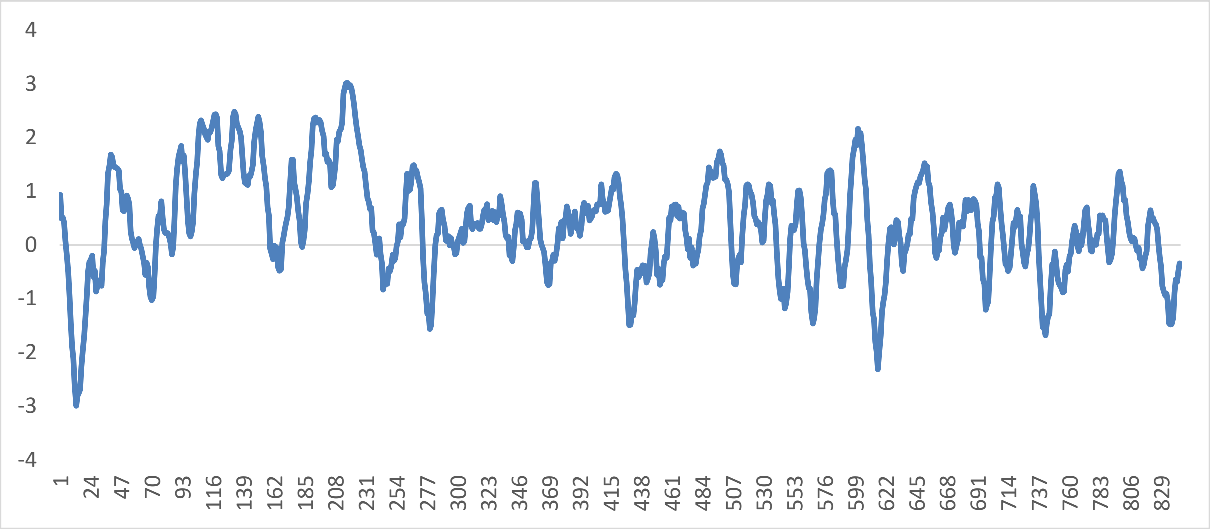
4
3
2
1
0
-1
-2
-3
-4
1
24
47
70
93
116
139
162
185
208
231
254
277
300
323
346
369
392
415
438
461
484
507
530
553
576
599
622
645
668
691
714
737
760
783
806
829

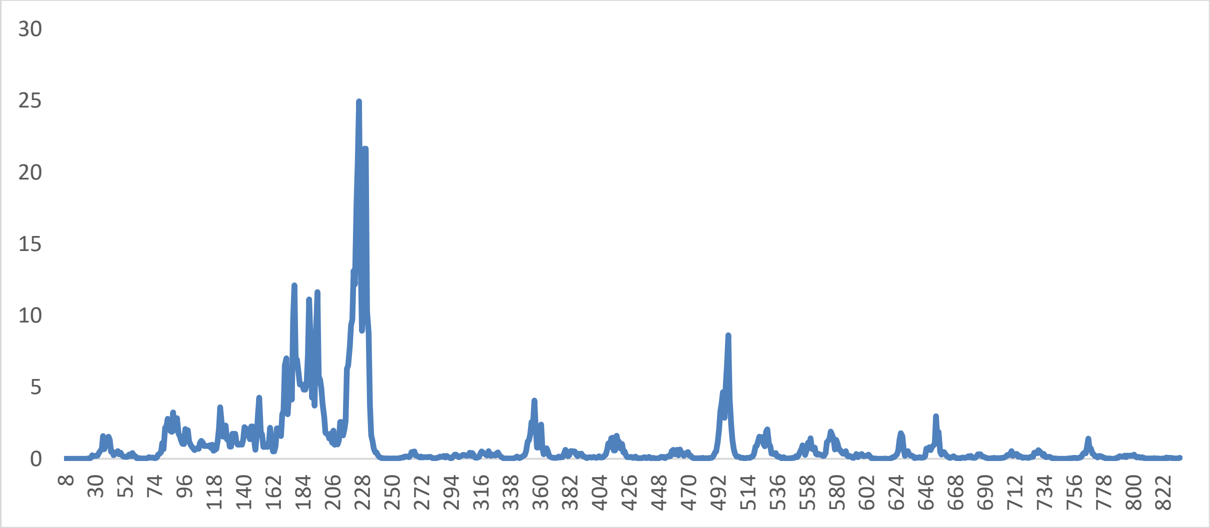
30
25
20
15
10
5
0
8
30
52
74
96
118
140
162
184
206
228
250
272
294
316
338
360
382
404
426
448
470
492
514
536
558
580
602
624
646
668
690
712
734
756
778
800
822

K

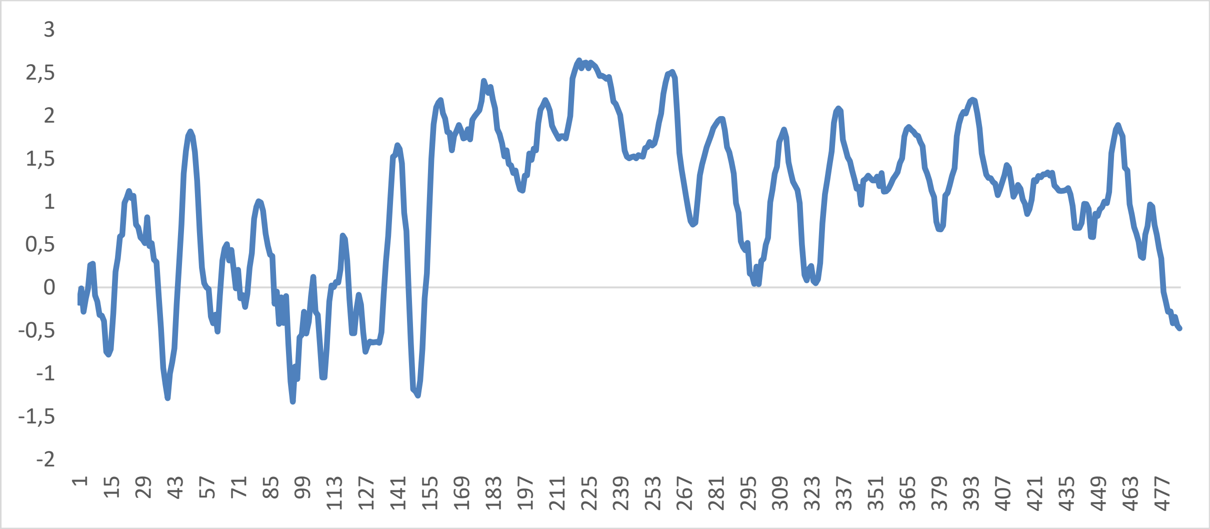
3
2,5
2
1,5
1
0,5
0
-0,5
-1
-1,5
-2
1
15
29
43
57
71
85
99
113
127
141
155
169
183
197
211
225
239
253
267
281
295
309
323
337
351
365
379
393
407
421
435
449
463
477

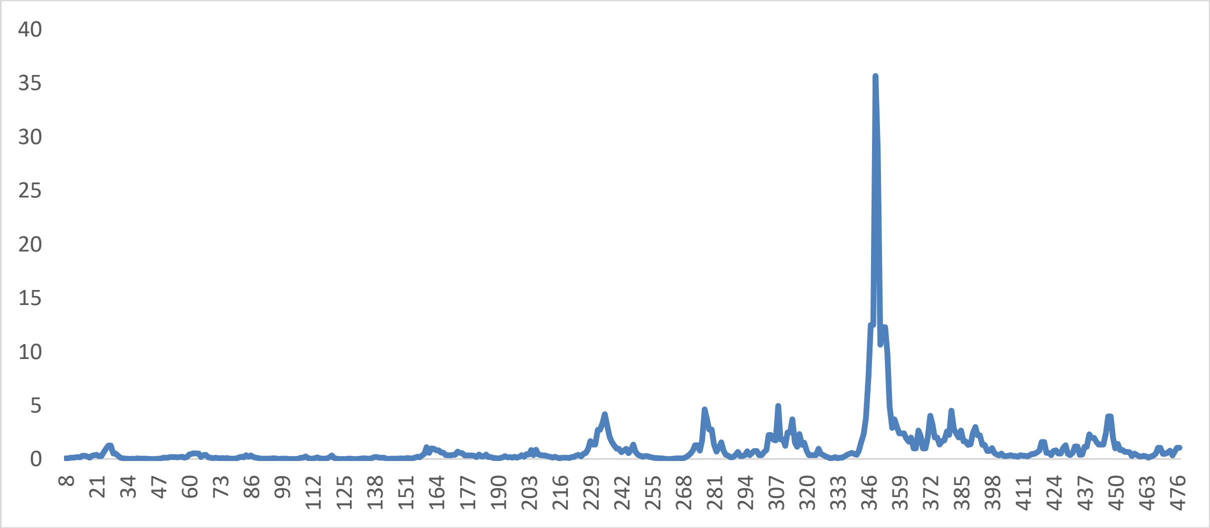
40
35
30
25
20
15
10
5
0
8
21
34
47
60
73
86
99
112
125
138
151
164
177
190
203
216
229
242
255
268
281
294
307
320
333
346
359
372
385
398
411
424
437
450
463
476

L

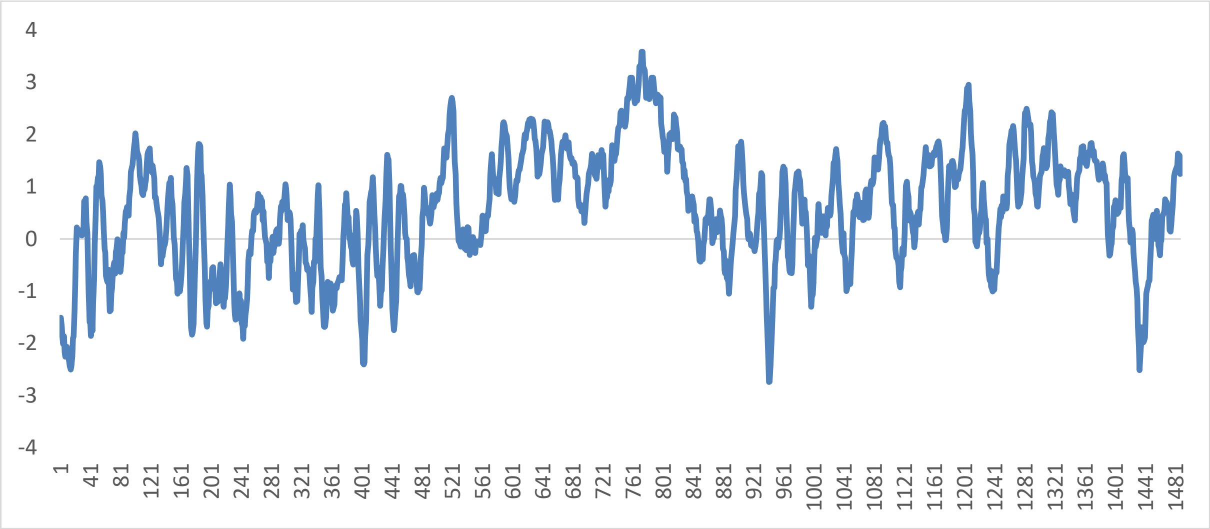
4
3
2
1
0
-1
-2
-3
-4
1
41
81
121
161
201
241
281
321
361
401
441
481
521
561
601
641
681
721
761
801
841
881
921
961
1001
1041
1081
1121
1161
1201
1241
1281
1321
1361
1401
1441
1481

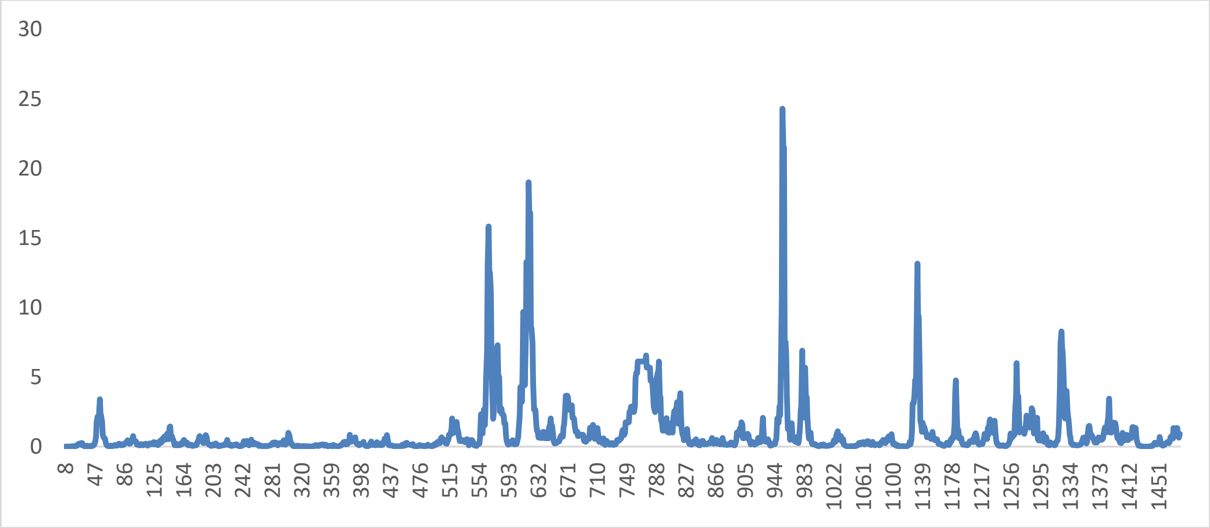
30
25
20
15
10
5
0
8
47
86
125
164
203
242
281
320
359
398
437
476
515
554
593
632
671
710
749
788
827
866
905
944
983
1022
1061
1100
1139
1178
1217
1256
1295
1334
1373
1412
1451

M

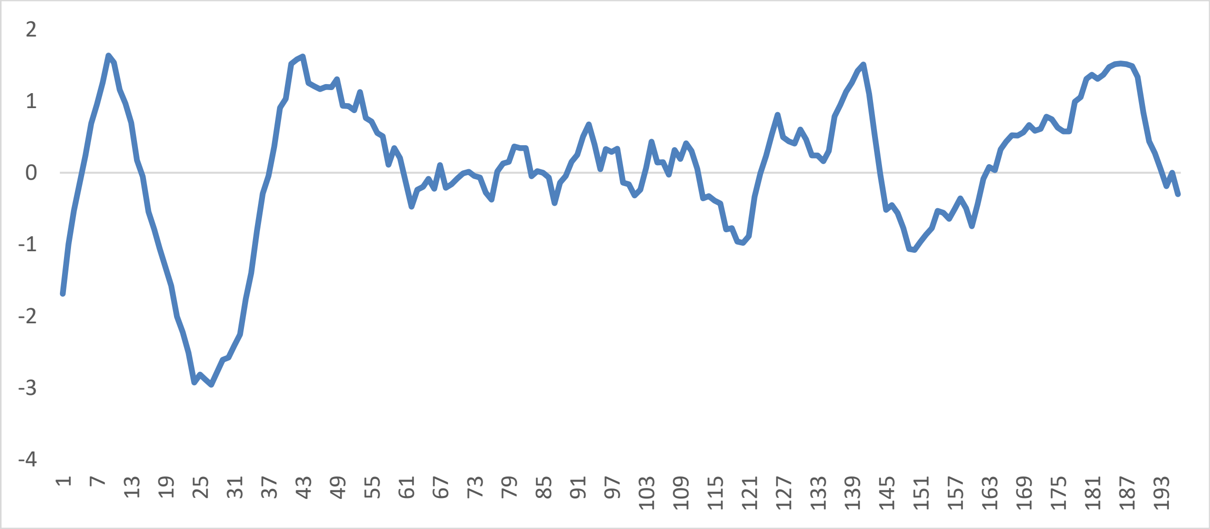
2
1
0
-1
-2
-3
-4
1 7 13 19 25 31 37 43 49 55 61 67 73 79 85 91 97 103 109 115 121 127 133 139 145 151 157 163 169 175 181 187 193

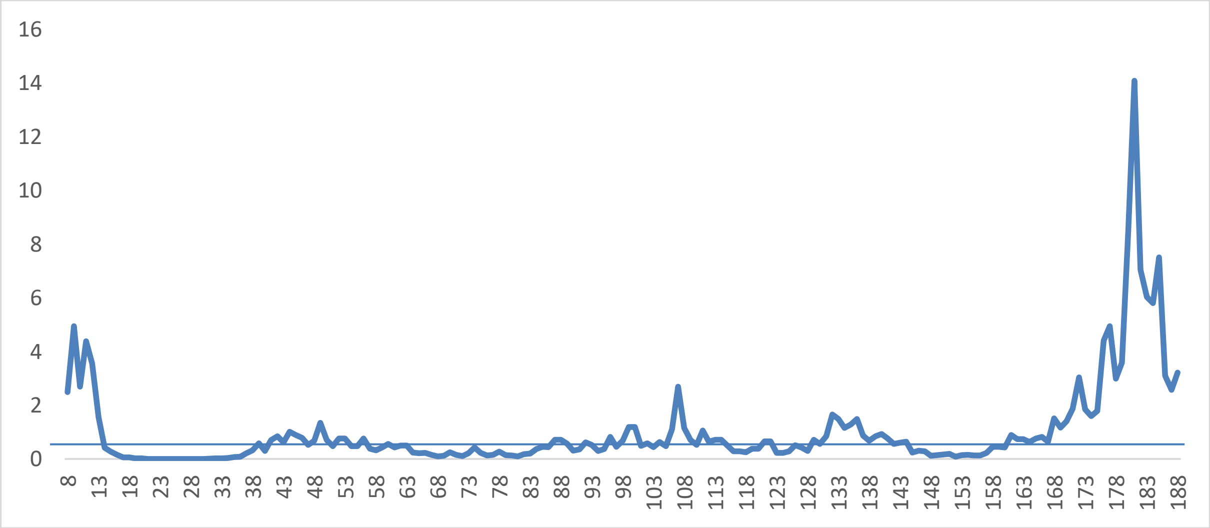
16
14
12
10
8
6
4
2
0
8 13 18 23 28 33 38 43 48 53 58 63 68 73 78 83 88 93 98 103 108 113 118 123 128 133 138 143 148 153 158 163 168 173 178 183 188

N

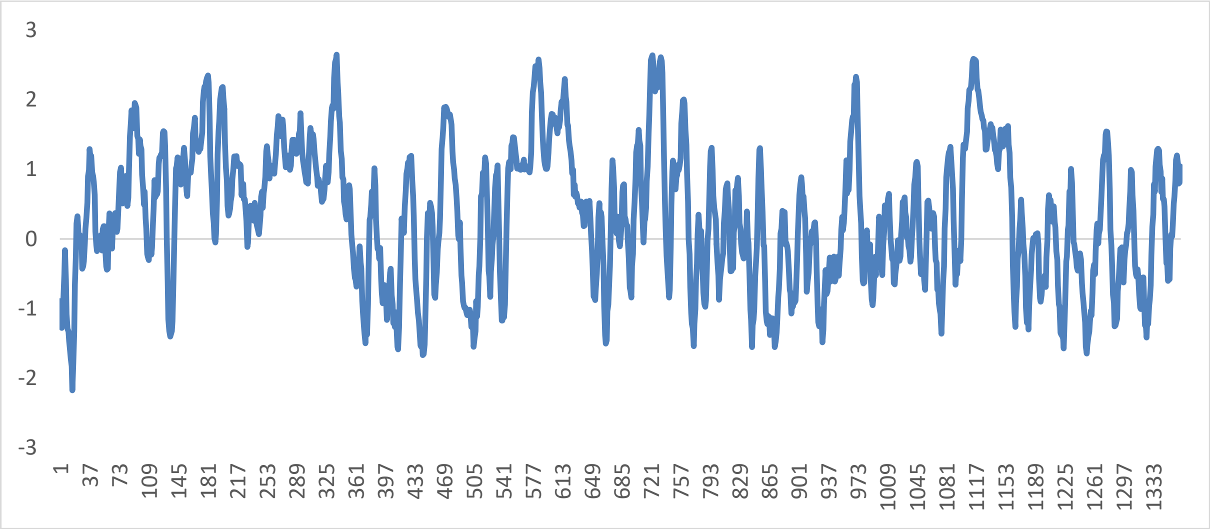
3
2
1
0
-1
-2
-3
1
37
73
109
145
181
217
253
289
325
361
397
433
469
505
541
577
613
649
685
721
757
793
829
865
901
937
973
1009
1045
1081
1117
1153
1189
1225
1261
1297
1333

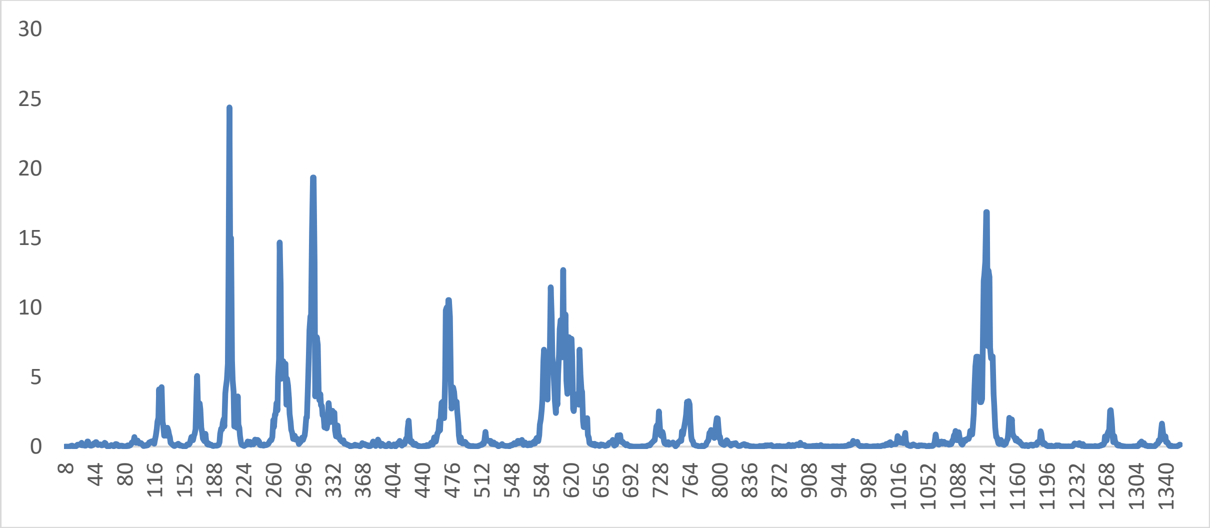
30
25
20
15
10
5
0
8
44
80
116
152
188
224
260
296
332
368
404
440
476
512
548
584
620
656
692
728
764
800
836
872
908
944
980
1016
1052
1088
1124
1160
1196
1232
1268
1304
1340

Supplementary Material 4. Linear B-epitopes and surface accessibility scores. A. *siap1* (PVX_000815), B. *p52* (PVX_001020), C. *p36* (PVX_001025), D. *spatr* (PVX_002900), E. *trsp* (PVX_081560), F. *trap* (PVX_082735), G. *spect1* (PVX_083025), H. *siap2* (PVX_088860), I. *maebl* (PVX_092975), J. *plp1* (PVX_000810), K. *mcp1* (PVX_111355), L. *tlp* (PVX_113965), M. *celtos* (PVX_123510), L. *mb2* (PVX_080420).
